# Structural Outlier Detection and Zernike–Canterakis Moments for Molecular Surface Meshes—Fast Implementation in Python

**DOI:** 10.3390/molecules29010052

**Published:** 2023-12-21

**Authors:** Mateusz Banach

**Affiliations:** Department of Bioinformatics and Telemedicine, Faculty of Medicine, Jagiellonian University Medical College, Medyczna 7, 30-688 Kraków, Poland; mateusz.banach@uj.edu.pl

**Keywords:** bioinformatics, computational geometry, molecular surface, Numba, principal component analysis, protein structure, Python, shape retrieval, Zernike moments

## Abstract

Object retrieval systems measure the degree of similarity of the shape of 3D models. They search for the elements of the 3D model databases that resemble the query model. In structural bioinformatics, the query model is a protein tertiary/quaternary structure and the objective is to find similarly shaped molecules in the Protein Data Bank. With the ever-growing size of the PDB, a direct atomic coordinate comparison with all its members is impractical. To overcome this problem, the shape of the molecules can be encoded by fixed-length feature vectors. The distance of a protein to the entire PDB can be measured in this low-dimensional domain in linear time. The state-of-the-art approaches utilize Zernike–Canterakis moments for the shape encoding and supply the retrieval process with geometric data of the input structures. The BioZernike descriptors are a standard utility of the PDB since 2020. However, when trying to calculate the ZC moments locally, the issue of the deficiency of libraries readily available for use in custom programs (i.e., without relying on external binaries) is encountered, in particular programs written in Python. Here, a fast and well-documented Python implementation of the Pozo–Koehl algorithm is presented. In contrast to the more popular algorithm by Novotni and Klein, which is based on the voxelized volume, the PK algorithm produces ZC moments directly from the triangular surface meshes of 3D models. In particular, it can accept the molecular surfaces of proteins as its input. In the presented PK-Zernike library, owing to Numba’s just-in-time compilation, a mesh with 50,000 facets is processed by a single thread in a second at the moment order 20. Since this is the first time the PK algorithm is used in structural bioinformatics, it is employed in a novel, simple, but efficient protein structure retrieval pipeline. The elimination of the outlying chain fragments via a fast PCA-based subroutine improves the discrimination ability, allowing for this pipeline to achieve an 0.961 area under the ROC curve in the BioZernike validation suite (0.997 for the assemblies). The correlation between the results of the proposed approach and of the 3D Surfer program attains values up to 0.99.

## 1. Introduction

The Protein Data Bank (PDB) [1,2] entered the year 2023 with a historical achievement of 200,000 molecular structures deposited in the central database. This number grew to 210,000 in September 2023 [3]. It is another milestone, because 210,000 constitutes 50.01% of the total number of possible PDB codes. Yet, it pales in comparison to over 250 million of known protein sequences available in the UniProt database [4]. This discrepancy comes from the fact that in order to obtain the 3D model of a protein, one needs to perform a crystallographic experiment, which requires time, funds, and expertise. AlphaFold2 [5,6] bridged this gap a bit by bringing to the PDB over a million (≈1,070,000 in October 2023) of the so-called Computed Structure Models (CSMs), which were predicted from sequence by this robust machine learning package (it outperformed its competition during CASP14 [7]). CSMs are auxiliary to the main, solved experimentally repository, and can be switched on and off during the search queries on the PDB website.

Anyone working in the field of structural bioinformatics eventually encounters the problem of measuring how two proteins are similar or dissimilar in terms of a chosen set of criteria. This task organically extends to the search for all molecules from a given database with properties within the range of the input molecule. Furthermore, the elements of this database can be clustered (or otherwise assigned to some categories) on the basis of their pairwise similarity metrics. The difficulty of these tasks comes not only from the number of items to process, but also the fact that the properties of proteins are often not directly comparable (e.g., sequences), requiring specialized algorithms that can be both difficult to program and have nonlinear complexity, translating to long run times.

The biological function of a protein can be inferred from its sequence or its structure. The structure also has higher evolutionary conservation than the sequence [8]. This degeneracy of the fold code gave rise to the idea that not all details of the sequence are important to reach the native conformation [9], and that the role of the environment in which the folding takes place must be considered [10]. The probability that two proteins fold alike increases with the percentage of the identity of their sequences. A 40% identity over a long alignment is sufficient to distinguish between similar and dissimilar structures [11]. Databases such as CATH [12] and SCOP [13] rely on this to organize the protein domains in forests depending on their structural, genetic and functional features. In turn, this information helps with the analysis and the classification of the proteins at the quaternary structure level. The 3D Complex database [14] clusters the biological assemblies from the PDB on the basis of the topology of graphs of their inter-chain contacts, domain codes and sequence identity.

In the so-called twilight zone, at 20–35% of identity, the link between the sequence and the structure becomes too weak for reliable sequence-based homology detection [8,11,15]. Likewise, a pair of biological assemblies with the same stoichiometry and domain composition but low sequence identity may exhibit vastly different complex symmetries. This phenomenon can be observed among the clusters at the QS (quaternary structure) Family level of 3D Complex hierarchy. The consequence is that accurate protein structure retrieval (i.e., recognition of molecules that folded in similar manner) requires structural comparison. With the ever-growing number and complexity of the models deposited in the PDB, this calls for the development of fast and scalable approaches.

Atomic superposition algorithms [16,17,18,19,20] are powerful tools for the structural comparison of proteins, but they can only gauge the difference between the two molecules they take as their input. Applying them in bulk (e.g., in an entire PDB vs. entire PDB scenario) is prohibitively time consuming. In an alternative approach, the proteins can be reduced to low-dimensional fingerprints of their shapes that can be compared in a massively reduced amount of time, possibly down to a constant number. This encoding organically scales to the database level, permitting rapid mass-scale molecule retrievals.

While the concept of shape is not well defined at the molecular level [20], it is typically (but also conveniently) taken as a function of the density of the atomic volumes. Van der Waals spheres provide this volume while bonds provide the connectivity graph. By rolling a sphere representing the water molecule (i.e., with a radius of 1.4 Å) over the vdW spheres, one produces the molecular surface of the protein, also known as its solvent-excluded or Conolly surface [21]. The computer model of this surface is a polygonal mesh. Alternatively, the volume of the protein can be approximated with an axis-aligned grid of voxels (e.g., 1 Å^3^ cubes) that intersect the vdW spheres. A relatively simple form of this voxelization is the central element of our Ellipsoid Profile (EP) algorithm for the measurement of the change in globularity in protein structures [22,23]. More advanced grid-based volumetric models can be obtained with EDTSurf (Euclidean Distance Transform) [24].

**Figure 1:**
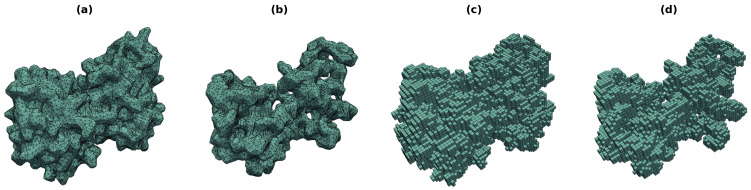
Common volumetric representations of a protein structure: (**a**) molecular surface mesh of all atoms, (**b**) molecular surface mesh of backbone atoms, (**c**) voxel grid of all atoms, and (**d**) voxel grid of backbone atoms. The protein has the PDB code 1KFV (chain A). The mesh on (**a**) has 13,670 vertices, 27,284 facets and a 33,504 Å^3^ volume. The mesh on (**b**) has 12,747 vertices, 25,578 facets and a 13,248 Å^3^ volume. The grids on (**c**,**d**) have 24,785 and 11,588 unit voxels, respectively.

The molecular surface and the cloud of voxels of chain A of Formamido-pyrimidine DNA Glycosylase from *Lactococcus lactis* (PDB code: 1KFV [25]) are shown in Figure 1. Both representations are typically calculated for all (heavy) atoms of the protein, but one may choose to obtain them only for the backbone atoms: N, C_α_, C, and O. The difference is that the all-atom surface mode encodes the overall shape of the molecule, whereas the backbone mode also captures the details of its interior by tracing the conformation of the chains.

Once the geometric representation of a protein is established, the generic shape retrieval methods [26] can be applied to it. The PDB website offers an option to search for the similarly shaped biological assemblies since 2020 [2]. It finds them using BioZernike [27], a protein structure retrieval system based on Zernike–Canterakis (ZC) moments.

The Zernike moments are noise-resistant projections of an arbitrary scalar field that describes the shape of a 2D object onto a set of orthogonal functions defined within the unit disk [28]. These functions, the Zernike polynomials, are named after Nobel Prize laureate Frits Zernike who delineated them in the 1930s [29]. At the turn of the century, Nikolaos Canterakis generalized them to the third dimension [30]. Three-dimensional Zernike moments, more accurately called Zernike–Canterakis moments [20,31], are defined inside a unit ball. In 2004, Marcin Novotni and Reinhard Klein [32] showed how to obtain them from a linear combination of the geometric moments of a voxelized volume. Geometric moments, used extensively in pattern recognition [33,34], are easy but relatively expensive to compute. The simplest shape-defining function that goes along them is binary, yielding 1 for the occupied and 0 for the unoccupied voxels. Novotni and Klein also showed how to base the 3D object retrieval on the ZC moments by compressing those moments into rotation-invariant feature vectors (i.e., the aforesaid low-dimensional fingerprints). They named the components of those vectors 3D Zernike descriptors (3DZDs). Once again, it is more accurate to call them Zernike–Canterakis descriptors (ZCDs) [20,31].

Since ZC moments form an infinite series of expansion of the original function of shape (i.e., they allow its reconstruction), a cutoff—the moment order—must be given by the user. Its de facto standard value in structural bioinformatics is *N* = 20 [20,27,35]. At *N* = 20, the number of geometric moments of a discretized 3D shape is 1771. The corresponding ZC moments capture most of the prominent features of the structure of a protein, akin to a low-resolution X-ray; see Figure 1 from [20] for comparison.

The number of ZC descriptors at *N* = 20 is 121. If two shapes are identical, their ZCDs are identical. The more those shapes differ, the more their ZCDs grow distant. Due to the fixed length of ZCD vectors (ZCDVs) at a given *N*, shape comparison in the ZC domain is very fast. The distance of one ZCDV to the ZCDVs of the entire database (e.g., the PDB) can be measured in linear time. It is the essence of the ZC-based protein shape matching. 3D Surfer [35] was the first widely available tool to implement it. Its creators recently employed the ZCDs in the mapping of the protein universe [36], in the classification of the CSMs [37] and during SHREC 2020/21 [38,39]. BioZernike [27] uses longer, less compressed ZC descriptors and feeds the retrieval with additional geometric data (e.g., the size of the molecule). Because the proteins must be scaled to fit inside the unit ball, these geometric features compensate for the loss of size-related information. ZC moments can also help with structural superposition. The ZEAL program [20] performs pairwise protein alignments by finding the transformation that maximizes correlation between ZC moments.

In 2011, José Pozo, Maria-Cruz Villa-Uriol and Alejandro Frangi proposed [31] an alternative approach to ZC moment calculation, directly from the polygonal surface of the mesh that determines the 3D shape, without voxelization. It is not only convenient, but it also helps with some of the issues of voxel-based solutions, namely the cubic complexity in respect to the width of the grid [31], and the “discretization error, which escalates as the order of the moments increases, and makes the scale and rotation invariants obtained from the moments only approximately invariant” [31]. In the algorithm of Pozo et al., the geometric moments are computed by integrating over the volumes of oriented tetrahedra spanned between the origin and the facets of the mesh. This step requires O($fN^{6}$) time, where *f* is the number of facets. In 2012, Patrice Koehl significantly reduced its complexity to O($fN^{3}$) [34], down to the level of practical application in mass-scale object retrieval. The optimized algorithm is thus called the Pozo–Koehl algorithm, PK for short [40].

One who wishes to calculate ZC moments for themselves encounters the issue of deficiency of reliable, configurable libraries readily available for inclusion in custom programs (i.e., not as external binaries), notably those written in Python. Novotni and Klein implemented their algorithm in C++, and BioZernike is written in Java [41]. Owing to the many useful libraries [42], Python is often found in scientific projects. For instance, AlphaFold is written in this language [43]. While there are packages in PyPi related to the “zernike” keyword [44], none of them work in 3D. A web search for “3D Zernike Python” leads to the question on StackExchange about ZC moments in molecular surface comparison [45]. It mentions the PK algorithm in Mindboggle [46], a Python brain morphometry platform, but cites its slowness. This is where our work enters the scene.

Here, we present a well-written and documented, fast Python implementation of the PK algorithm. It relies on Numba [47], a just-in-time Python-to-machine code compiler for a ≈200 times faster calculation at moment order 20 with respect to the code that only relies on NumPy’s vectorizations [48]. On a consumer-grade PC, our PK-Zernike library is capable of processing about 50,000 facets per second per thread. A mesh of this complexity corresponds to the high-density molecular surface of a protein composed of ≈600 residues, or a simplified surface of a protein with ≈1200 residues. This is fast enough for full-scale database analysis. Because the facets can be processed in any order, parallel execution is also possible. By relinquishing the GIL (Python’s global interpreter lock), the PK-Zernike library can use threads, which are lightweight and more convenient than the usual method of GIL circumvention in CPU-bound tasks (i.e., based on a pool of subprocesses).

The citations on the publisher’s website [31,34] suggest that the PK algorithm is less popular than the NK (Novotni–Klein) algorithm [32], and that it has not been used yet in structural bioinformatics. To assess its usefulness in this area, we made it the central element of a novel, simple, but effective protein structure retrieval pipeline that can be implemented using the “baseline” Python libraries for scientific computation [48,49]. This pipeline comprises the calculation of molecular surface meshes via an external program, and the comparison of their ZCDVs and additional geometric data: the distribution of the residues in the structure, its size and the volume of its mesh. Tests were carried out in the BioZernike validation repository [50] and on several example proteins. Lastly, the results of this retrieval were put against the results of retrieval based on ZCDs produced by the 3D Surfer program for an indirect comparison of PK and NK algorithms.

While ZC descriptors are invariant to the rotations of the input shape, they are susceptible to its scaling and translation. Aderinwale et al. [37] gave examples of structures in which protruding fragments (e.g., chain termini fully exposed to the solvent) can confuse the purely ZC-based retrieval systems, making it harder for them to recognize proteins that belong to the same fold. This issue can be worked around with neural networks [37]. Here, the proteins were passed through an outlier residue detection subroutine from the EP algorithm [23]. This subroutine, the principal component analysis (PCA [51]) and Kabsch’s superposition algorithm [52,53] are based on the same paradigm. This method provided a major boost to the sensitivity of the retrieval with only a small loss of specificity and a very low computational cost, thus warranting its inclusion in the title of this document.

### 1.1. Structure of the Manuscript

The Materials and Methods section describes the protein structure datasets (Section 3.1) and the algorithms, libraries and programs utilized in this research (Section 3.2 and Section 3.3). The Results section begins with the presentation of the capabilities of our fast PK-Zernike library (Section 2.1). Next is the description of the novel protein structure retrieval pipeline based on the PK algorithm (Section 2.2). The last three subsections of the Results assess the discrimination ability of this pipeline in the BioZernike validation repository (Section 2.3), in the set of 30 fragments of example proteins (Section 2.4), and in comparison with the results of the 3D Surfer program, also obtained for the example structures (Section 2.5).

### 1.2. Definitions and Conventions

The structures from the PDB are referred to in this paper by their PDB codes rather than names, for example, 1KFV instead of Formamido-pyrimidine DNA Glycosylase.

Individual chains are denoted by the XXXX:Y notation, in which XXXX is the PDB code and Y is the chain identifier (e.g., 1KFV:A). The two alternative notations for multi-chain selections are XXXX:Y+…+ Y (an explicit list, e.g., 1KFV:A+D+G) and XXXX:Y−Y (an implicit list, e.g., 1KFV:B−H). Sub-chain selections end with a residue range, for example, 1KFV:(A+B)(30–100) captures residues 30–100 from chains A and B from 1KFV.

A biological assembly is the largest, functionally-relevant fragment of a protein (i.e., its quaternary structure) that was obtained during the crystallographic experiment and is available in a given PDB file [54,55]. Each PDB file should have at least one such assembly defined in its REMARK 350 records. 1KFV has two: 1KFV_1 and 1KFV_2; they are instances of the same protein–nucleic complex. In this paper, the first manually assigned (i.e., not only matched by software) assembly was taken as the representative of each whole PDB file from Section 3.1.2. In this sense, 1KFV ≡ 1KFV_1. Some of those assemblies needed to be reconstructed via the symmetry operators from the REMARK 350 records. They are distinguished here by the *×n* notation. It informs that the selected group of chains had to be transformed *n* times to produce the final structure of the complex.

The term protein complex should be understood in the context of this work as the reference to two or more protein chains from a given biological assembly that have non-bonded contacts between them. Interactions between the proteins and the non-protein entities (e.g., nucleic acids, ligands or ions) were outside the scope of this research.

An effective atom of a residue is its representative pseudo-atom—a point in space at the average position of all heavy atoms from this residue (i.e., sans hydrogen and deuterium). This definition is shared with FOD [56,57] and EP algorithms [22,23].

An unstructured surface mesh is the surface of a 3D shape made up from vertices (i.e., points in space) and explicitly defined facets (i.e., polygons) that connect them. The meshes in this study had triangular facets, as required by the PK algorithm.

To decimate a mesh is to simplify its surface. It is achieved by algorithmically decreasing the number of vertices and facets by the reduction factor specified by the user (e.g., by 50%) while trying to retain the shape and volume of the original model.

Lastly, a Zernike–Canterakis descriptor vector (ZCDV) is a feature vector composed of rotation-invariant Zernike–Canterakis descriptors (ZCDs) [58]. It is the low-dimensional representation of Zernike–Canterakis (ZC) moments of given 3D shape. Here, ZCDs were calculated following the “3DZD” formula popularized by Novotni and Klein [32].

## 2. Results and Discussion

### 2.1. The Fast Python Implementation of the PK Algorithm

This section presents our time-efficient Python implementation of the Pozo–Koehl (PK) algorithm [31,34] for the calculation of Zernike–Canterakis moments from the exact volume-like geometric moments of an unstructured surface mesh (Section 3.2.4).

A note on vocabulary: PK-Zernike is the name of the library, PKZernike is the name of the main calculation class, and pkzernike is the name of the Python package. The source code is available at https://codeberg.org/mbanach/pkzernike since 22 October 2023.

A reference Python implementation of the PK algorithm was obtained from the Mindboggle library [46]. It is based on NumPy [48] and SciPy [49]. Its shapes.zernike module [59] is a 2013 port of the Matlab code by Arthur Mikhno [60]. Brian Rossa and Arno Klein are credited for this conversion. There are several PK classes (a.k.a. pipelines) in Mindboggle; the fastest two are called KoehlOptimizations and KoehlMultiproc. The latter is a parallel version of KoehlOptimizations; it utilizes Python’s built-in multiprocessing module.

#### 2.1.1. Geometric Moments

The most time-consuming portion of the ZC pipeline is the calculation of geometric moments. Its complexity in the PK algorithm is O($fN^{3}$), where *f* is the number of facets of the mesh and *N* is the moment order. It is optimal [34], but doing it in Python, even with the fast NumPy vectorizations (i.e., optimized functions that can operate on whole arrays) is too slow for a practical application in structural bioinformatics. Because the facets can be processed in any order, Mindboggle reduces the calculation time by managing a pool of parallel workers. However, this engages the entire CPU while still taking too long for the molecular surface content. The parallel execution helps with the O(*f*) component but not with the O($N^{3}$) component of algorithmic complexity. On the flip side, the memory utilization of the PK algorithm is very efficient, only O($N^{3}$).

Our approach takes on the cubic time complexity with Numba, a just-in-time (JIT) compiler capable of automatic translation of math-heavy functions and loops to optimized machine code [47]. Owing to Numba, programs stay written in Python, but at runtime they achieve the CPU performance of programs written in C or Fortran. To reach this speed, one must carefully organize the Python code (possibly rewriting most of it) to avoid any unsupported features. For instance, the numpy.compress function is unavailable in Numba and its own version of the numpy.roll function does not accept the axis parameter.

While working around the aforesaid code restrictions, we found that the shortest run times happen when the facets of the mesh are passed to the geometric moment function in bulk. It produces a (*N*+1) × (*N*+1) × (*N*+1) array using two loops. The outer loop iterates the facets while the inner loop populates the *C*, *D* and *S* arrays for each facet. The elements of *S* are multiplied by the volume of the oriented tetrahedron and added to the output array. Speed is gained by exploiting the fact that the inner loop does not need to undergo all 0≤i,j,k≤N indices of the geometric moments, but only those for which i+j+k≤N [32]. At *N* = 20, there are 1771 (22.1% of 8000) of such i,j,k tuples.

#### 2.1.2. The Coefficient Cache

The PKZernike class populates and caches two arrays when it is instantiated. The maximum moment order must be given by the user. The first array holds binomial coefficients, nk, whereas the second array holds trinomial coefficients, which are given by the (a|b|c)=(a+b+c)!/(a!b!c!) equation. This procedure is also accelerated with Numba. But because Numba has no support for factorial functions (e.g., scipy.special.factorial), the binomials and trinomials are calculated iteratively using Pascal’s triangle and Pascal’s pyramid, respectively. Overall, the cache is generated in O($N^{3}$) time and requires O($N^{3}$) space. It can also be copied from another PKZernike object to avoid recalculation.

#### 2.1.3. ZC Moments

Cached binomial and trinomial coefficients accelerate the next stage of the PK algorithm that produces ZC moments from geometric moments. Its complexity is O($N^{4}$) due to the four nested loops for each of the *V*, *W*, *X*, *Y* and *Z* arrays. The trinomial cache also helps with geometric moments as each i,j,k element of their array must be multiplied by the (i!j!k!)/(i+j+k+3)! coefficient. We again avoid the factorials by noticing that this coefficient is equal to ${\left[(i|j|k)\left(n+1\right)\left(n+2\right)\left(n+3\right)\right]}^{-1}$, where n=i+j+k. We also locally cache all temporary variables, so that the innermost loops (i.e., at depth 4) never call any math-heavy NumPy function (e.g., numpy.sqrt) on individual numbers. It significantly speeds up this part of the algorithm at *N* ≤ 20, even without Numba’s JIT.

#### 2.1.4. ZC Descriptors

The last portion of the PKZernike class that is accelerated by Numba calculates the ZC descriptors in the sense of Novotni and Klein [32]. Its algorithmic complexity is O($N^{3}$). Once compiled to a machine code, its running time, like in the case of the functions of the coefficient cache and of ZC moments, becomes negligible, measured in milliseconds.

#### 2.1.5. The Compilation

While Numba delivers an outstanding computational speed boost with no need for changing the programming languages or interpreters (and it is multi-platform), it does not come without its own cost. Both just-in-time and ahead-of-time compilation modes incur an overhead that increases with the complexity of the Python code and of the NumPy functions this code calls. The compilation occurs only once per interpreter process, but its delay is noticeable enough to warrant additional optimizations. Our PC needs this amount of time to compile the four calculation functions in the PKZernike class:

1. ≈1.4 s for the geometric moment function;
2. ≈0.7 s for the coefficient cache function;
3. ≈1.4 s for the ZC moments function;
4. ≈1.15 s for the ZC descriptors function.

Once everything is compiled (in over 4.5 s), points 2–4 together finish in less than 0.001 s at *N* = 20 and in ≈0.005 at *N* = 35. However, the cache is generated without the acceleration in ≈0.01 s at *N* = 20 and in ≈0.05 s at *N* = 35. Likewise, if only the geometric moment function is compiled, ZC moments are obtained from geometric moments in ≈0.05 s at *N* = 20 and in ≈0.3 s at *N* = 35. A total of ≈0.002 s is then needed to calculate their ZCDs at *N* = 20 (≈0.004 s at *N* = 35). This benchmark indicates the existence of two use cases: a single mesh and multiple meshes. When *N* ≤ 25 and the application processes a single mesh and exits, it is more time-efficient to only compile the geometric moment function. But when there are multiple meshes to process, or when the moment order is high, at least the ZC moment function should be compiled, too. This can save a lot of time during mass-scale object retrieval. For example, the processing of all structures from the PDB on our PC in the single mesh mode would incur almost 3 h of surplus CPU time (210,000 × 0.05 s). With a compiled ZC moments function, this overhead drops to 3 min. The best times, however, are achieved with Numba’s compilation cache. The cache directory can be specified via the NUMBA_CACHE_DIR environment variable, or by overwriting numba.config.CACHE_DIR. The fully compiled PKZernike class is then instantiated in less than 0.001 s. Loading the cache has a one-time delay of ≈0.175 s.

#### 2.1.6. The Comparison

**Figure 2:**
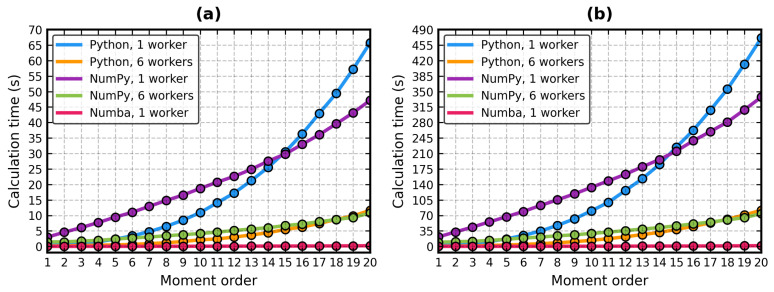
Time needed by the proposed and reference implementations of the PK algorithm to return the ZC descriptors of (**a**) 1SZT (5988 vertices, 11,944 facets) and (**b**) 1AVO (39,123 vertices, 78,043 facets). The surface meshes were calculated in the all-atom mode and decimated by 50%.

Figure 2 shows the amount of time needed by the serial and parallel run modes of the implementations of the PK algorithm to calculate the ZC descriptors for the all-atom surface meshes of 1SZT (a representative of small proteins) and 1AVO (a representative of medium-sized proteins) at moment orders 1–20. The primary factor contributing to this time was the geometric moments function. In the figure, “Python” means PKZernike without the acceleration (i.e., code employing pure Python loops), “NumPy” means KoehlOptimizations and KoehlMultiproc from Mindboggle (i.e., code relying on NumPy’s vectorizations), and “Numba” means PKZernike with full acceleration (i.e., all functions compiled).

At *N* ≤ 10, there was a noticeable overhead in KoehlOptimizations. The time needed by the plain, serial PKZernike class then rapidly increased, eventually surpassing KoehlOptimizations at *N* = 15. A similar situation, albeit at a smaller scale, happened in the parallel mode. As one may expect, the machine code vastly outperformed the Python loops and the NumPy-only classes. At *N* = 20, the single-core, Numba-powered PKZernike was over 45 times faster than KoehlMultiproc and over 200 times faster than KoehlOptimizations. Its speed gain versus itself in the “Python” mode was ×50 with six workers and ×300 with one worker. This shows the amount of time that can be saved by Numba in math-heavy tasks with nested loops, marking PK-Zernike a cost-efficient tool for pythonic protein structure analysis. There was only the matter of checking its accuracy and numerical stability. Mindboggle’s classes were taken as the gold standard for this purpose.

In this paper, if *A* and *B* are two shapes (e.g., two proteins), the difference between their ZC descriptor vectors (ZCDVs, both of length *l*) is symbolized with $\Delta_{z}$(*A*, *B*):

(1) $$\Delta_{z}\left(A,B\right)=\sum_{i=1}^{l}|\mathrm{zcd}\left(A,i\right)-\mathrm{zcd}\left(B,i\right)|.$$

$\Delta_{z}$ is the Manhattan distance between the two ZCDVs, and “zcd” is a hypothetical subroutine of the program that returns the value of the *i*th ZCD of a given shape. Equation (1) is used throughout the manuscript as the baseline shape distance function.

The $\Delta_{z}$ of the ZCDVs of the same protein produced by PK-Zernike and by the reference implementation in Mindboggle was in the $10^{-17}$ range at *N* = 1. It grew to $10^{-10}$ at *N* = 20, and to ≈0.002 at *N* = 35. We suspect the cause of this discrepancy to be the different order of additions and multiplications in the internal equations. An error of the same magnitude was observed between the KoehlOptimizations and KoehlMultiproc classes, and between the serial and parallel runs of PKZernike. Thus, it can be considered a normal behavior, with no significant impact on the proposed protein structure retrieval (Section 2.2).

All tested approaches exhibited numerical instabilities above order 35, as expected for PK and NK algorithms with the standard double precision arithmetic [40]. While it is a limiting factor, it did not pose an obstacle for the presented retrieval, since the (small) benefit of the more detailed protein shape encoding at *N* > 35 is offset by a considerably longer calculation time, approximately ×3 at *N* = 30 and ×5 at *N* = 35.

As it was demonstrated in Figure 2, akin to the Mindboggle library, the run time of the PKZernike class can be further reduced by dispatching a pool of workers to calculate the geometric moments in parallel. The facet array is split into chunks of equal size. The partial results are collected from the pool and added in an out-of-order manner to the final geometric moment array. But because Numba can release Python’s GIL (if nogil = True is passed to numba.jit), PKZernike objects can employ threads instead of processes. This avoids the need to fork the Python interpreter for each worker and reduces the inter-process communication overhead. The speed gain is proportional to the number of threads running on the physical CPU cores (e.g., six threads means roughly calculation time/6).

### 2.2. The PK Algorithm in Protein Structure Retrieval

This section describes the novel protein structure retrieval pipeline centered around the fast implementation of the PK algorithm in the PK-Zernike library (Section 2.1).

The primary purpose of this task was to assess the usefulness of the PK algorithm in structural bioinformatics as the provider of the Zernike–Canterakis moments (as opposed to the NK algorithm in a voxel-based workflow), with the calculation of those moments done in a fully pythonic environment (n.b., we consider the use of Numba to conform to this view, since it is a proper, multi-platform Python library, albeit requiring compilation of its C++ code when installed from the source). An external program had to be called only to generate the molecular surface meshes of the proteins. Its contribution to the run time of the pipeline was 1–2× the time of the single-threaded run of PK-Zernike after the meshes were decimated by 50% (the parsing of the PDB files was the third large time factor).

The second goal was to demonstrate that this pipeline, despite being relatively uncomplicated to make with the popular Python libraries and with PK-Zernike, can achieve the discrimination ability comparable with that of the state-of-the-art retrieval systems.

**Figure 3:**
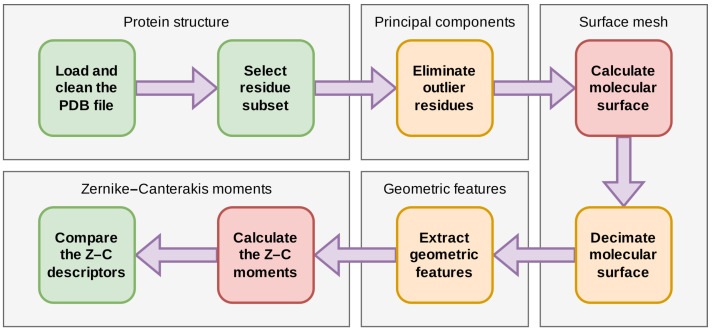
Flowchart of the proposed protein retrieval pipeline. This image was authored with draw.io v. 21.6.8, (https://github.com/jgraph/drawio-desktop, accessed on 31 August 2023).

The flowchart of the proposed pipeline is given in Figure 3. It has eight steps. Steps in green are rudimentary—they involve basic text parsing or numerical comparisons. Steps in orange require advanced programming, but can be performed with just a few calls to functions from dedicated libraries (a numerical package may provide a fast SVD subroutine, e.g., numpy.linalg.svd). Steps in red are challenging to code and need more time to complete in comparison to the other instructions on this flowchart.

#### 2.2.1. Preparation of the Protein Structure

After the PDB file with the structure is loaded, it must undergo the usual cleaning. Water molecules (HOH) are not needed, and the alternative atom locations are normalized by keeping only the first rotamer with the highest occupancy (typically A).

The application should also parse the MODRES records from the PDB header to tell the modified polymer residues and proper ligands apart. The link between the MODRESes and their standard parents is necessary for an accurate molecular surface calculation as the van der Waals radii are typically not provided for the first group.

#### 2.2.2. Selection of the Input Fragment

The user may now choose a specific fragment of the structure to be passed to the next steps of the pipeline. It may be the entire molecule (i.e., all chains), a single chain, a group of chains (not necessarily in non-bonded contact with each other), or a more sophisticated selection of residues, such as a domain or a beta sheet. The only limiting factor is whether the molecular surface package will be able to converge on this selection.

Here, all non-protein entities (nucleic acids, etc.) were removed from the input structures at this stage as they were out of scope of this research. Hydrogen and deuterium atoms were removed, too. They are ignored during the outlier residue detection and the molecular surface calculation, so there was no need for keeping them in memory.

#### 2.2.3. Detection of the Outlier Residues

The selected portion of the structure undergoes the PCA-based outlier residue detection procedure described in Section 3.2.1. Its default settings are recommended, namely *p* = 0.9 (i.e., the confidence level) and *r* = 3 (i.e., the number of detection rounds). Residues classified as outliers are excluded from the subsequent stages of the pipeline.

**Figure 4:**
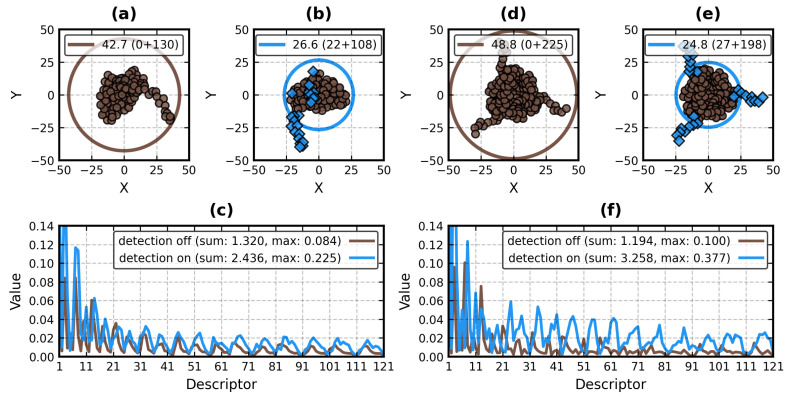
Change in the unit ball scale factor and in the ZC descriptors caused by the elimination of the outlier residues in 4B0H:A (**a**–**c**) and 1IIE:A–C (**d**–**f**). The values in the legends on (**a**,**b**,**d**,**e**) are the scale factor and the numbers of outlier (blue diamonds) and guide (brown circles) residues. The range of the Y axis on (**c**,**f**) is limited for visibility—“max” is the value of the tallest descriptor.

The main purpose of this step is the same as in the EP algorithm [22,23]—to eliminate the exposed, often disordered fragments of the structure that contribute little to its volume but artificially inflate its bounding shape (MVEE [61] in EP, sphere in ZC), skewing the metrics that rely on this shape. Figure 4 shows how strongly the presence of such fragments can affect the ZCDs of 4B0H:A (at P120–G131) and 1IIE:A−C (at Q184–K192).

This procedure may cause some of the external but not so strongly protruding residues to be discarded. It allows for the EP algorithm to attain more compact (i.e., tight) ellipsoidal representations of the proteins, counter-balancing the dependence of the MVEE algorithm on the shape and size of the convex hull of its input. This also benefits the protein structure retrieval, in particular when similarly-shaped molecules are considered. Such molecules are expected to share a common structural motif, but may differ in the details of their surface: a bent loop here, an extra helix there, etc. A controlled (i.e., at *r* = 3) removal of some of those details diminishes the differences and emphasizes the main similarities. This, in turn, increases the overall sensitivity of the retrieval more than it decreases its specificity. The evidence in favor of this conjecture is presented in Section 2.3.

#### 2.2.4. Calculation of Molecular Surfaces

The molecular surface of the remaining (i.e., guide) portion of the molecule is now calculated. Here, this task was carried out with Michel Sanner’s program [62] (details in Section 3.2.2). MSMS outputs text files with the vertex and facet arrays of unstructured triangle meshes. These meshes are passed as the input to the PK algorithm.

Two kinds of meshes were generated for the proteins in this experiment. The first mesh was based on all heavy atoms (i.e., in the all-atom mode, e.g., Figure 1a), while the second used only the backbone atoms (i.e., in the backbone atom mode, e.g., Figure 1b). In the backbone mode, the input protein was stripped down to its N, C_α_, C, O atoms and split into individual chains. Each chain was separately sent to MSMS, and the resulting set of meshes was merged into a single mesh. No special mesh merging method was needed, owing to the distance between the molecular surfaces of the backbones.

#### 2.2.5. Decimation of Molecular Surfaces

The resolution of the triangulated molecular surfaces produced by MSMS is controlled by –density and –hdensity parameters. Their default values deliver detailed meshes with a high uniformity of the triangles (Figure 1a,b). While the number of those facets is not overly high, even this level of detail was unnecessary for the current study. Thus, the resolution of all molecular surfaces was reduced by half (Section 3.2.3); 50% fewer facets in the mesh means a 50% shorter geometric moment calculation time.

Pozo et al. [31] showed that the adaptive mesh decimation has a lower error rate than the uniform decimation in conjunction with the exact, volume-like geometric moments. However, their tests involved smooth 3D models, whereas the molecular surface meshes are rugged and noisy. We found that when the shape difference between the original and the decimated molecular surface was measured with $\Delta_{z}$, depending on the reduction factor, the uniform algorithm delivered a 2–4 times lower value at *N* = 20 (and with overall lower standard deviation across different structures) than the adaptive algorithm.

**Figure 5:**
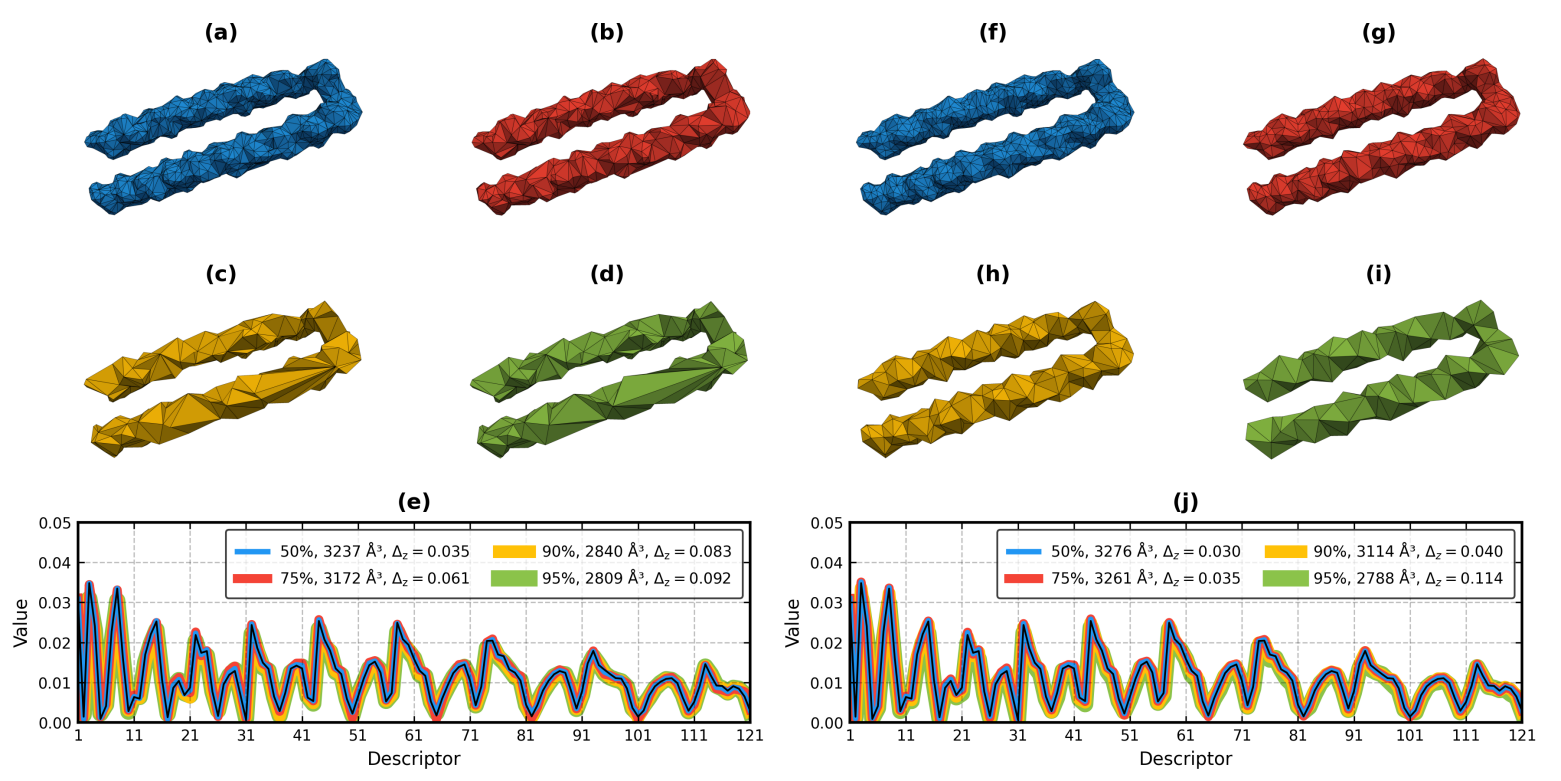
The impact of adaptive (**a**–**e**) and uniform (**f**–**j**) decimation of the backbone atom molecular surface of 1SZT:A on its ZC descriptors. The legends on (**e**,**j**) denote the reduction factor, the volume of the decimated mesh, and the $\Delta_{z}$ versus the undecimated MSMS output, represented by the black lines on (**e**,**j**). The volume of the original mesh was 3275 Å^3^.

The impact of the various reduction factors (50, 75, 90, and 95%) on the $\Delta_{z}$ of the molecular surface of 1SZT:A calculated for the backbone atoms is shown in Figure 5. The uniform decimation filter [63] stayed longer near the original volume of the mesh than the adaptive filter [64]. It was also able to keep the surface intact at the highest of the four factors, while the other algorithm disintegrated it when its topology preservation setting was not enabled (note that a broken mesh does not break the PK algorithm).

For the above reasons, we decided to rely on the uniform decimation in this experiment. While a conservative 50% mesh reduction was applied to all molecules, as exemplified by Figure 5, factors up to 90% are also viable options (90% equals a speed-up by an order of magnitude). In fact, the results from Section 2.5 show that the uniform decimation of the output of MSMS by 90% was fine for the retrieval of the example proteins.

Figure 5 is also a good example of the resistance of the PK algorithm (and of the ZC moments in general) to the noise caused by the simplification of the mesh—the descriptors drift apart from the original, but do not undergo sudden, spectacular alterations.

#### 2.2.6. Extraction of Geometric Features

ZC moments require all points of the shape (i.e., mesh vertices or voxel centers) to reside within the unit ball. The easiest way to fulfill this requirement is to shift the average position of these points to the origin, find their largest distance to the origin, $r_{\max}$, and divide their coordinates by $r_{\max}$. All unit ball scale factors discussed in this paper should be understood like this—as coordinate divisors. Unlike voxel-based methods, the meshes do not need to be pre-scaled for the PK algorithm to fill the grid of a specific size (e.g., $64^{3}$). They may, however, have more facets that the number of occupied voxels.

Citing poor resolution of ZC functions near the unit sphere [58], Ljung and André [20] set the scale factor in their ZEAL program to $r_{\max}/0.7$ (i.e., they increase $r_{\max}$ by ≈1.43). In BioZernike, Guzenko et al. [27] scale the structures by $1.8r_{g}$, where $r_{g}$ is the radius of gyration, the root mean square of the distance of C_α_ atoms to the center of the mass of the molecule. Ljung and André also point out that in their C++ library, Novotni and Klein [32] use $2r_{g}$ as the scale factor, which does not guarantee that the entire shape will fit inside the unit ball. $1.8r_{g}$ means that an even greater portion of the molecule may be left outside. There are two ways to address this: increase the scale factor to at least $r_{\max}$ or remove the outliers. The removal is less damaging to the voxelized representation of the proteins as it permits partial atomic volumes to remain inside the unit ball. Since it only affects the external voxels, it acts akin to the outlier detection procedure from Section 2.2.3, exchanging some of the details of the molecular surface for the increased sensitivity of the retrieval. It cannot, however, eliminate any strongly protruding fragments like those in 4B0H and 1IIE (Figure 4). On the other hand, removing a vertex of a mesh removes all facets it comprises. The volume-like variant of the PK algorithm expects the surface to be closed (i.e., hole-free), so its unit ball scale factors should not be lower than $r_{\max}$.

ZC moments encode the shape of a protein, but the necessary scaling diminishes the information coming from its size. To compensate, Guzenko et al. employ additional geometric features in their retrieval process, such as the distribution of atomic distances to the origin, the radius of gyration and the molecular weight. The exact PK algorithm based on the volume-like geometric moments is naturally predisposed to the accurate description of the interior of the proteins (within the limits of ZC formalism). But at the same time it relies on their molecular surface to delimit that interior. In the all-atom mode, only the overall shape of the structure is captured. Guzenko et al. stress the need for the encoding of its entire volume. This is why we calculated both all-atom and backbone atom molecular surfaces (Section 2.2.4). It gave us the possibility to observe the change in the specificity of the retrieval following the inclusion of polypeptide conformation data. Following Guzenko et al., we also collected three geometric features of each protein: its size, volume and the histogram of the distribution of effective atoms. The benefit of effective atoms is that they are invariant to the shape and complexity of the surface mesh of the molecule.

1. The distribution of residues inside the protein is approximated by the distribution of the distances of guide effective atoms (Section 2.2.3) to the origin after translation to origin. The necessary transformation is produced by the outlier detection algorithm from Section 2.2.3. The default distance histogram spans the 0–1000 Å range at 2 Å intervals. Its upper limit can be as high as needed, but we found 2 Å to be the optimal bin size. There is one problem: the locations of effective atom centroids may vary even between similar structures, leading to the overestimation of discrepancies between them. To counter-balance it, the histograms are smoothed with the Savitzky–Golay convolution [65] using a third-order polynomial and a window length of five. Any negative values it may produce must be clipped to zero. The smoothing diminishes some of the local zigzags while retaining the overall trend of distribution.
2. The size of a protein is encoded by standard deviations of its guide effective atoms in each dimension. The whole structure participates in this measurement (sans the outliers). The atoms need to be centered at the origin and rotated in alignment with the principal axes of the coordinate system to maximize their variance. Once again, this alignment can be easily achieved with the algorithm from Section 2.2.3.
3. The volume of a protein is simply the volume of its mesh prior to the unit ball scaling. It can be quickly calculated with PyVista/VTK [66,67], even if the surface is not continuous. Unlike the other two geometric features, it depends on the mesh rather than on the effective atoms. To approximate the volume of the effective atoms, one needs to voxelize the protein, which is something we wanted to avoid here.

If the atoms of the protein are not provided (only its mesh), the effective atoms can be replaced with the vertices of the mesh for the purpose of these geometric features.

Lastly, there is the question of the unit ball scale factor. Four candidate solutions were already mentioned: $r_{\max}$, $r_{\max}/0.7$, $1.8r_{g}$ and $2r_{g}$. Obviously, $r_{\max}$ must be calculated for the vertices of the mesh to ensure that they all stay inside the unit ball, but the other three factors can be based on (guide) effective atoms to make them mesh-invariant (this means that $r_{\max}$ in $r_{\max}/0.7$ was here the maximum distance of an effective atom, not of a vertex). Because the effective atoms are shifted away from the molecular surface, their $1.8r_{g}$ is too short, essentially turning it into $r_{\max}$. This leaves $r_{\max}$, $r_{\max}/0.7$ and $2r_{g}$.

Here, we present another unit ball scale factor: $r_{\mathrm{PCA}}$, defined as the largest of the three radii of a confidence ellipsoid of the guide effective atoms at p=0.95 ($\sqrt{s}$ ≈ 2.795483, Section 2.2.3). $r_{\mathrm{PCA}}$ is higly variable, and depending on the protein, it can reach values close to $r_{\max}$ (e.g., in 5AHE) or as high as $r_{\max}/0.7$ (e.g., in 1YAR). The reliance on the confidence level is both an advantage and a drawback—it allows and requires tuning of the algorithm, although *p* = 0.95 appears to be a safe default value.

**Figure 6:**
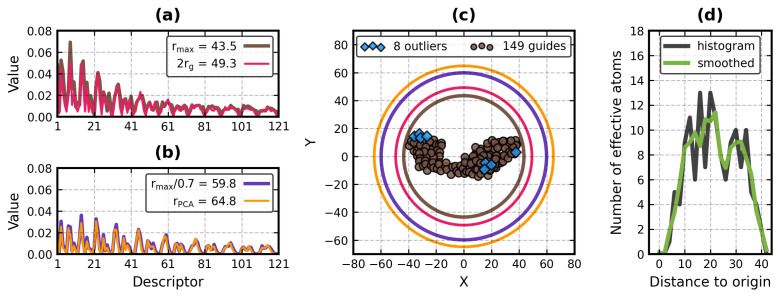
The impact of the unit ball scale factor on ZC descriptors of 1DIV:A: (**a**) $r_{\max}$ and $2r_{g}$, (**b**) $r_{\max}/0.7$ and $r_{\mathrm{PCA}}$. The corresponding balls before scaling down to the unit size are shown on (**c**). The distance histogram on (**d**) is independent of the scale factor. Its bin size is 2 Å.

Figure 6 shows how the different scale factors affect the ZCDs of 1DIV:A. It also shows an example effective atom distance histogram before and after smoothing.

#### 2.2.7. Calculation of ZC Moments

The vertices of the mesh are centered at the origin and their coordinates are divided by the chosen unit ball scale factor. If somehow this factor is smaller than $r_{\max}$ (Section 2.2.6), it is increased to $r_{\max}$. The entire mesh now resides inside the unit ball.

The fast Python implementation of the PK algorithm from Section 2.1 is called on the vertex and facet arrays of the mesh, producing an order 20 Zernike–Canterakis descriptor vector (ZCDV) in the sense of Novotni and Klein (i.e., with 121 ZCDs).

#### 2.2.8. Comparison of ZC Descriptors

If *A* and *B* are two shapes, here—molecular surface meshes of two proteins structures, the descriptors of their ZC moments produced by the PK algorithm should range from 0 to 1. In this experiment, before the difference between them was measured with Equation (1), each ZCDV was normalized by dividing it by the sum of all its ZCDs. This caused $\Delta_{z}$ to have a maximum value of 2. $\Delta_{z}$(*A*, *B*) = 2 happens when all ZCDs of *A* and *B* are 0 except for zcd(*A*, *i*) = 1 = zcd(*B*, *j*) and *i* ≠ *j*. It is an unlikely situation. However, the normalization made it easier to define universal $\Delta_{z}$-based structural similarity thresholds.

After the experimentation with example proteins (Section 3.1.2), we devised a similarity scale based on $\Delta_{z}$ with steps at 0.2 intervals between 0 and 1, plus a final step at 2. It is shown in Table 1. *A* and *B* had the same or nearly identical shape when $\Delta_{z}$(*A*, *B*) < 0.2. No structural similarity should be expected between them when $\Delta_{z}$(*A*, *B*) ≥ 1.

1YAR:(A−U)×2 was the only structure that the scale from Table 1 marked as vastly different from the other example proteins in Figure S31 in Supplemental File S3. It achieved $\Delta_{z}$≥ 0.6 with all of them and $\Delta_{z}$≥ 0.8 with most of them. The other uniquely shaped protein in this set, 1AV1:A−D, had a medium similarity score (i.e., Tier 3) versus the third of the examples. With the exception of 1AIK:(N+C) and 1SZT:A, Tier 1 results were accurate—the nearly identical shapes were paired without false positives. But there were too many false positive results in the second and third tiers, expected to only capture the rest of the similar structures with the same domain superfamily classification. This confirms that the shape encoding via ZC moments alone is insufficient for a robust protein structure retrieval system and must be supplied with additional geometric features to improve its discrimination power. Three such features were described in Section 2.2.6: the smoothed histogram of the effective atom distances to the origin, the size of the molecule and the volume of its surface mesh. Here, we show how they can be incorporated to Equation (1) while staying compatible with the interpretation in Table 1.

$\Delta_{d}$ gauges the difference between the first smoothed and then normalized effective atom distance histograms of *A* and *B*:

(2) $$\Delta_{d}\left(A,B\right)=\sqrt{\sum_{b=0}^{b_{\max}}{\left[\mathrm{hist}\left(A,b\right)-\mathrm{hist}\left(B,b\right)\right]}^{2}}.$$

“Hist” is a hypothetical subroutine of the program that returns the smoothed and normalized number of guide effective atoms with distance to the origin in the [2b, 2b+2) range, where *b* is the bin index and 2 is the bin width. $b_{\max}$ should be high enough (e.g., 500) to contain the largest of the input structures. Alternatively, if the histograms have different lengths, the shorter one can be padded with trailing zeros for the same effect.

$\Delta_{\mathrm{zd}}$ is simply the sum of values of $\Delta_{z}$ and $\Delta_{d}$:

(3) $$\Delta_{\mathrm{zd}}\left(A,B\right)=\Delta_{z}\left(A,B\right)+\Delta_{d}\left(A,B\right).$$

Normalization of histograms and the Euclidean distance in Equation (2) prevents $\Delta_{d}$ from dominating $\Delta_{z}$ while allowing for $\Delta_{\mathrm{zd}}$ to push apart proteins that have a similar shape in the sense of ZC descriptors but different distributions of residues inside them.

$\Delta_{s}$ gauges the difference between the sizes of *A* and *B*:

(4) $$\Delta_{s}\left(A,B,\gamma_{s}\right)=\prod_{d=1}^{3}\left[\frac{\max\left(\mathrm{size}\left(A,d\right),\mathrm{size}\left(B,d\right)\right)}{\min\left(\mathrm{size}\left(A,d\right),\mathrm{size}\left(B,d\right)\right)}+\gamma_{s}\right].$$

“Size” is a hypothetical subroutine of the program that measures the distribution of atomic density in a given protein in the *d*th dimension. Here, it was the standard deviation of the guide effective atoms after they were rotated in alignment with the principal axes of the coordinate system. It does not matter that this coefficient is smaller than the actual length of the structure; it could also be the radius of the confidence ellipsoid.

Larger size coefficients in Equation (4) are divided by smaller size coefficients. The three fractions are multiplied, but before that, $\gamma_{s}$ is added to each of them. It is a tune parameter controlled by the user. Its suggested default value is −0.05.

$\Delta_{v}$ gauges the difference between the volumes of *A* and *B*:

(5) $$\Delta_{v}\left(A,B,\gamma_{v}\right)=\frac{\max\left(\mathrm{vol}\left(A\right),\mathrm{vol}\left(B\right)\right)}{\min\left(\mathrm{vol}\left(A\right),\mathrm{vol}\left(B\right)\right)}+\gamma_{v}.$$

“Vol” is a hypothetical subroutine of the program that returns the volume of a given mesh before its placement inside the unit ball. The idea is the same as in Equation (4): the larger volume is divided by the smaller volume, and the ratio is added to $\gamma_{v}$, which shifts it up or down. The suggested default value of $\gamma_{v}$ is −0.1.

$\Delta_{s}$ and $\Delta_{v}$ are combined with $\Delta_{\mathrm{zd}}$ via multiplication, yielding $\Delta_{\mathrm{zds}}$ and $\Delta_{\mathrm{zdv}}$:

(6) $$\Delta_{\mathrm{zds}}\left(A,B,\gamma_{s}\right)=\Delta_{\mathrm{zd}}\left(A,B\right)\cdot \Delta_{s}\left(A,B,\gamma_{s}\right),$$

(7) $$\Delta_{\mathrm{zdv}}\left(A,B,\gamma_{v}\right)=\Delta_{\mathrm{zd}}\left(A,B\right)\cdot \Delta_{v}\left(A,B,\gamma_{v}\right).$$

$\Delta_{s}$ and $\Delta_{v}$ act as penalty coefficients for $\Delta_{\mathrm{zd}}$ when $\gamma_{s}$ ≥ 0 and $\gamma_{v}$ ≥ 0. Setting $\gamma_{s}$ or $\gamma_{v}$ to a negative value allows the shape distance between similar structures measured with $\Delta_{\mathrm{zds}}$ or $\Delta_{\mathrm{zdv}}$ to move towards the upper rows of Table 1.

Finally, $\Delta_{\mathrm{zdsv}}$ is the combination of $\Delta_{\mathrm{zd}}$ with both $\Delta_{s}$ and $\Delta_{v}$:

(8) $$\Delta_{\mathrm{zdsv}}\left(A,B,\gamma_{s},\gamma_{v}\right)=\Delta_{\mathrm{zd}}\left(A,B\right)\cdot \Delta_{s}\left(A,B,\gamma_{s}\right)\cdot \Delta_{v}\left(A,B,\gamma_{v}\right).$$

$\Delta_{s}$ and $\Delta_{v}$ have the lowest discrimination ability alone, which is why they are not combined with $\Delta_{z}$ directly, but through $\Delta_{\mathrm{zd}}$. $\Delta_{d}$ is also weak on its own, but becomes an important factor in $\Delta_{\mathrm{zd}}$. This leaves $\Delta_{z}$, $\Delta_{\mathrm{zd}}$, $\Delta_{\mathrm{zds}}$, $\Delta_{\mathrm{zdv}}$ and $\Delta_{\mathrm{zdsv}}$ as the useful shape distance functions. It should also be noted that all of the presented equations are trivially vectorizable, facilitating quick searches in the protein databases. If the value of any of those equations surpasses 2 (which is expected for dissimilar proteins when $\Delta_{s}$ and $\Delta_{v}$ are in effect), it should be clipped to 2, to keep it synchronized with Table 1.

### 2.3. Results of the Retrieval—The BioZernike Repository

The protein structure retrieval pipeline presented in Section 2.2 has several combinations of settings: (1) the molecular surface mode: all atoms, backbone atoms; (2) the surface mesh decimation factor: fixed here at 50%; (3) the outlier residue detection: enabled (3 rounds), disabled (0 rounds); (4) the unit ball scale factor: $r_{\max}$, $r_{\max}/0.7$, $2r_{g}$, $r_{\mathrm{PCA}}$; and (5) the shape distance function: $\Delta_{z}$, $\Delta_{\mathrm{zd}}$, $\Delta_{\mathrm{zds}}$, $\Delta_{\mathrm{zdv}}$, $\Delta_{\mathrm{zdsv}}$. While one may surmise that the backbone atoms and outlier detection enabled are the right choices, the choice of the unit ball scale factor and the distance function is non-trivial. Hence, to determine the optimal settings of this pipeline, and to compare its discrimination ability with the state-of-the-art algorithms, we turned to the BioZernike validation repository [50] and its three test suites: “CATH”, “ECOD” and “assemblies”. The database and the testing protocol are described in Section 3.1.1. Guzenko et al. [27] provided the ROC curves and the areas underneath them (AUROCs) for the retrievals they performed with BioZernike, 3D Surfer [35] and Omokage [68] algorithms (the Omokage search is a non-Zernike biomolecular structure retrieval system based on histograms of interatomic distances).

The 240 ROC curves (2 × 1 × 2 × 4 × 5 settings per each of the three test suites) are shown in Supplemental File S2, in Figures S15–S30. Figure 7 is the copy of Figure S28.

**Figure 7:**
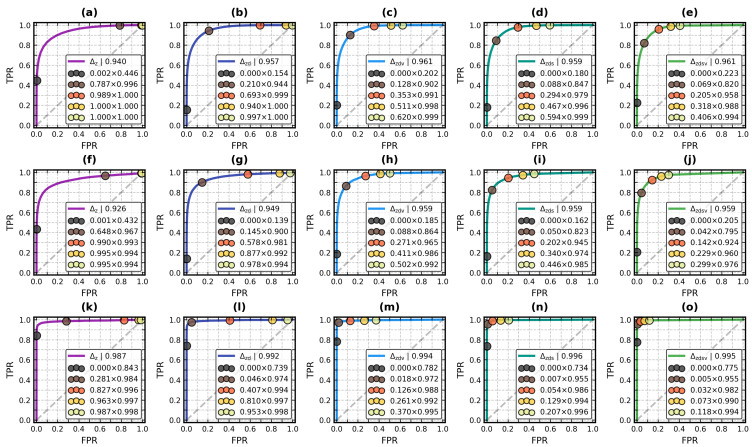
ROC curves for the proposed protein structure retrieval pipeline with the highest sum of the $\Delta_{\mathrm{zdsv}}$ AUROCs in CATH (**a**–**e**), ECOD (**f**–**j**) and assembly (**k**–**o**) BioZernike test suites. The settings were as follows: backbone atom mesh, outlier detection on, and $2r_{g}$ as the unit ball scale factor. The legends denote the shape distance function, the AUROC and the coordinates of the markers. Colors of those markers correspond to the upper bounds in Table 1 below which the structures were considered similar. A scalable version of this figure is available as Figure S28 in Supplemental File S2.

Figure 7 presents the ROC curves of the retrieval performed with the following settings: molecular surface of backbone atoms, outlier detection enabled, and $2r_{g}$ as the unit ball scale factor. This combination of settings achieved the highest sum of AUROCs using $\Delta_{\mathrm{zdsv}}$ as the shape distance metric: 0.961 in the CATH, 0.959 in the ECOD and 0.995 in the assembly suite. AUROCs for the other scale factors were 0.957, 0.959 and 0.995 for $r_{\mathrm{PCA}}$ (Figure S30e,j,o), 0.954, 0.958 and 0.994 for $r_{\max}/0.7$ (Figure S26e,j,o), and 0.952, 0.954 and 0.996 for $r_{\max}$ (Figure S24e,j,o). While the best of these numbers are lower than the AUROCs reported for BioZernike (0.97 for ECOD and 1.0 for the assemblies [27]), they were reached in an easily programmable and modifiable ab initio experiment (Guzenko et al. also reported AUROCs of 0.93 for ECOD and 0.95 for assemblies with 3D Surfer, and 0.84 and 0.99 with Omokage). We believe it is a promising result that may be optimized further, for example, by introducing another geometric feature. Overall, $2r_{g}$ had a higher TPR and FPR of the similarity thresholds than $r_{\mathrm{PCA}}$. With $\Delta_{\mathrm{zdsv}}$, the FPR difference between these two scale factors was up to ≈0.03 and the TPR difference was up to ≈0.05.

Molecular surfaces based on backbone atoms indeed permit more accurate domain assignment prediction than all-atom surfaces. AUROCs in Figure 7a,f (i.e., for $\Delta_{z}$) are 0.940 and 0.926, while the corresponding values in Figure S20a,f are only 0.869 and 0.871. While the overall shape of domains from different superfamilies may be similar, the backbone traces unveil their internal structure, allowing for the ROC curves to stay closer to the left boundary of the ROC space. The opposite happened in the assembly suite, although with a much smaller magnitude; 0.987 in Figure 7k and 0.992 in Figure S20k are an example of it. $\Delta_{\mathrm{zdsv}}$ AUROCs for all-atom surfaces were also a bit higher, at 0.997 for all scale factors (Figures S16o, S18o, S20o and S22o). It rounds to 1.0, the same as for BioZernike.

Turning on the outlier detection subroutine moved all curves toward the top of the ROC space. Without it, but with $2r_{g}$ and the backbone atom surface, AUROCs dropped to 0.874, 0.877 and 0.967 ($\Delta_{z}$, Figure S27a,f,k). This means that the outlier detection is as important as the type of molecular surface. The small loss of specificity it incurred (up to ≈0.02 for $\Delta_{\mathrm{zdsv}}$) was followed by a massive gain of sensitivity (up to ≈0.1 at Tier 2). It is a worthy trade-off with a linear time cost with respect to the number of residues.

It was confirmed that $\Delta_{z}$ has the lowest discrimination ability of the five tested shape distance functions. When combined with outlier detection turned off, all-atom surface and $r_{\max}$ (i.e., the baseline settings, Figure S15a,f,k), it performed the worst, with AUROCs of 0.746, 0.777 and 0.947. Switching to the backbone atoms changed these values to 0.789, 0.828 and 0.945, respectively (Figure S23a,f,k). Turning on the outlier detection caused $\Delta_{z}$ AUROCs to rise to 0.811, 0.833 and 0.988 with all-atom surfaces (Figure S16a,f,k), and to 0.874, 0.885 and 0.983 with backbone atom surfaces (Figure S24a,f,k).

Another significant improvement was brought with the introduction of $\Delta_{d}$—by the augmentation of the shape distance function from $\Delta_{z}$ to $\Delta_{\mathrm{zd}}$. Once again, it confirms the need for the inclusion of the geometric data of the proteins for accurate retrieval. AUROCs for baseline settings increased a lot with $\Delta_{\mathrm{zd}}$, to 0.870, 0.889 and 0.972 (Figure S15b,g,l). The switch to backbone atoms set these numbers to 0.871, 0.900 and 0.971 (Figure S23b,g,l). Outlier detection moved them even further up, to 0.923, 0.934 and 0.995 (all atoms, Figure S16b,g,l), and to 0.929, 0.935, and 0.992 (backbone atoms, Figure S24b,g,l). Discounting $\Delta_{z}$, all shape distance functions at the Tier 1 threshold of structural similarity (i.e., the black markers) had the lowest TPR but also an FPR below 0.0005.

Introduction of $\Delta_{s}$ and $\Delta_{v}$ to the retrieval brought an increase in its specificity at the expense of its sensitivity, an effect similar to when the outlier detection was enabled, only with loss rather than gain of TPR and FPR. However, the net outcome was positive, with a higher area under the ROC curve with respect to $\Delta_{\mathrm{zd}}$. Also, in comparison to $\Delta_{\mathrm{zd}}$, all structural similarity threshold markers were shifted towards the beginning of the $\Delta_{\mathrm{zdv}}$ and $\Delta_{\mathrm{zds}}$ curves (Figure 7c,d,h,i,m,n), capturing far fewer false positive results and somewhat fewer true positive results. This phenomenon was independent of the surface mode and of the unit ball scale factor. It was stronger with $\Delta_{\mathrm{zds}}$ than with $\Delta_{\mathrm{zdv}}$. The reason for this is obvious: two meshes may have similar volumes but different sizes.

The markers of Tier 2 threshold on $\Delta_{\mathrm{zd}}$ curves were closest to the top left corner of the ROC space (e.g., the brown marker in Figure 7b,g,l). This threshold observed the highest TPR loss on $\Delta_{\mathrm{zdv}}$ and $\Delta_{\mathrm{zds}}$ curves for the domains, up to ≈0.1, but only up to ≈0.025 for the assemblies. It was compensated by the strong movement of higher-tier markers to the left (i.e., FPR reduction). They formed the least spread clusters near point [0, 1] when $\Delta_{s}$ and $\Delta_{v}$ were combined with $\Delta_{\mathrm{zd}}$ to $\Delta_{\mathrm{zdsv}}$ (Figure 7e,j,o), especially in the case of assemblies. It seems that with the surface mesh calculated for backbone atoms, and with outlier detection enabled, regardless of the unit ball scale factor, the point on the $\Delta_{\mathrm{zdsv}}$ curve closest to the “best” corner of the ROC space lay somewhere in the Tier 3 range, from 0.4 to 0.6.

### 2.4. Results of Retrieval—Example Proteins

An optimal combination of settings of the protein structure retrieval pipeline proposed in Section 2.2 was determined in Section 2.3: the surface mesh of backbone atoms, outlier residue detection enabled, either $r_{\mathrm{PCA}}$ (lower TPR and FPR) or $2r_{g}$ (higher TPR and FPR) as the unit ball scale factor, and $\Delta_{\mathrm{zdsv}}$ as the primary shape distance metric. For an in-depth analysis of the results this pipeline can deliver, it was applied to a collection of 30 fragments of example proteins from Section 3.1.2. These fragments represent biological assemblies, chains and chain complexes with various shapes, sizes, domain classifications and levels of structural similarity. They are listed in Table 2 with additional experimental data.

**Figure 8:**
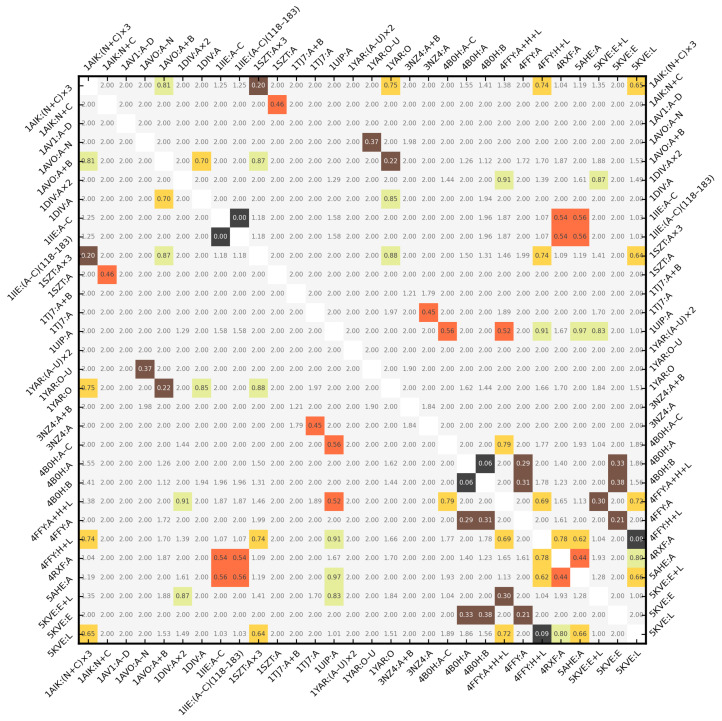
$\Delta_{\mathrm{zdsv}}$ heatmap for the 30 inputs from Table 2 (435 pairs). The data are mirrored on both sides of the diagonal. Color scale of the shape distance: black—[0,0.2), brown—[0.2,0.4), orange—[0.4,0.6), yellow—[0.6,0.8), green—[0.8,1.0), gray—[1.0,2.0], the same ranges as in Table 1. A scalable version of this figure is available as Figure S35 in Supplemental File S3.

$r_{\mathrm{PCA}}$ was chosen as the unit ball scale factor in this experiment. ZCDVs of the 30 inputs from Table 2 were compared with each other, yielding 435 values of $\Delta_{z}$, $\Delta_{\mathrm{zd}}$, $\Delta_{\mathrm{zds}}$, $\Delta_{\mathrm{zdv}}$, and $\Delta_{\mathrm{zdsv}}$. These values are presented in form of symmetric heatmaps in Supplemental File S3, in Figures S31–S35. Figure 8 is the copy of Figure S35.

As expected, $\Delta_{\mathrm{zdsv}}$ exhibited the strongest discrimination ability—Figure 8 is sparse (i.e., mostly gray, $\Delta_{\mathrm{zdsv}}$ ≥ 1). If one considers $\Delta_{\mathrm{zdsv}}$ = 0.5 to be the similarity threshold, all but 4 pairs of input structures were scored in accordance with their domain classification in the sense of the SCOP/CATH superfamily. The increment of this threshold to 0.6 added 6 more false positives. However, 4 of those 10 incorrect results were technically duplicates, since they involved 4B0H:A/4B0H:B and 1IIE:A−C/1IIE:(A−C)(118–183), which are parts of same proteins (e.g., 4B0H:A ≈ 4B0H:B(2-131)).

Using one worker thread, our PC took 17.8 s to calculate all 30 ZCDVs (9.6 without 1YAR). With six threads, these times were reduced to 3.1 and to 1.7 s, respectively (note: those are only runtimes of the PKZernike class). The average vertex-to-facet ratio in the decimated surface meshes was 0.5 (σ = 0.002), and the correlation coefficient between the number of facets and the volume of the mesh was 0.999 (0.994 sans 1YAR:(A−U)×2).

The outlier subroutine procedure removed the C-termini from 1IIE:(A−C), reducing it to 1IIE:(A−C)(118–183), and causing their $\Delta_{\mathrm{zdsv}}$ to become 0. The removal of C-termini in 4B0H:A and 4B0H:B overcame the issue of truncation of chain A, leading to a $\Delta_{\mathrm{zdsv}}$ of 0.06 (otherwise it would be 0.36). 4FFY:H+L and 5KVE:L (i.e., the mouse antibodies) received $\Delta_{\mathrm{zdsv}}$ = 0.09. This confirms that the two chains in 4FFY structurally match the one chain in 5KVE. Outlier detection can be disabled for an increased specificity at the cost of a lowered sensitivity of the retrieval. It may be beneficial when one is seeking exact or near-exact matches, for example, to find 4B0H:C but not 4FFY:A when 4B0H:B is the query structure. In this case, the important shape information is kept in the outlier region.

The high similarity score (0.2 ≤ $\Delta_{\mathrm{zdsv}}$ < 0.4) was achieved by 1AIK:(N+C)×3 and 1SZT:A×3 (their $\Delta_{\mathrm{zdsv}}$ = 0.201 was close to Tier 1), 1AVO:A−N and 1YAR:O−U, 1AVO:A+B and 1YAR:O, 4FFY:A+H+L and 5KVE:E+L, 4FFY:A and 5KVE:E, and by 4B0H:A/4B0H:B and 4FFY:A and 5KVE:E. The last group comprised the first four (two non-redundant) false positive results due to mismatched domain classification. It happened because the chains of 4B0H without C-termini are similar to the viral proteins, albeit larger, as corroborated by the CE RMSD of 5.42 Å over 80 residues. They are, however, CATH distorted sandwiches (2.70), whereas 4FFY:A and 5KVE:E are “regular” sandwiches (2.60). Any trace of similarity between them is lost ($\Delta_{\mathrm{zdsv}}$ ≥ 1) when the outliers are not removed.

The medium similarity score (0.4 ≤ $\Delta_{\mathrm{zdsv}}$ < 0.6) was assigned to 1AIK:N+C and 1SZT:A, 1IIE:A−C/1IIE:(A−C)(118–183) and 4RXF:A and 5AHE (two non-redundant false positives), 1TJ7:A and 3NZ4:A, 1UIP and 4B0H:A−C and 4FFY:A+H+L (two false positives), and 4RXF:A and 5AHE. $\Delta_{s}$ = 1.41 was a significant factor in $\Delta_{\mathrm{zdsv}}$ = 0.46 for 1AIK:N+C and 1SZT:A. 1TJ7:A and 3NZ4:A have three CATH domains, two of them with matching superfamilies. SCOP puts those proteins as a whole in one superfamily by an automated match. Despite their differences, they received a $\Delta_{\mathrm{zdsv}}$ = 0.45. Conversely, 4RXF:A and 5AHE belong to different SCOP superfamilies, but CATH assigns them to the same superfamily. 1UIP is their cousin in SCOP fold c.1 and in CATH topology 3.20.20. Yet, it exhibited incorrect closeness to 4B0H:A−C and 4FFY:A+H+L, although both times $\Delta_{\mathrm{zdsv}}$ was above 0.5. The medium structural similarity between 1IIE:(A−C)(118–183) and 4RXF:A and 5AHE was easier to point out, both under the visual inspection and via the CE RMSD of 5.86 Å over 72 residues. Like them, 1IIE:(A−C)(118–183) features a central “tunnel” in the surface of its backbone atoms, only surrounded by helices rather than sheets. Its $\Delta_{\mathrm{zdsv}}$ versus each of these proteins was also above 0.5. The PDB website reports the intact molecule of 1IIE:A−C (i.e., with its disordered C-termini present) to have a unique shape in the entire database. Likewise, the outlying C-terminal helix makes 4RXF:A appear much different from 5AHE ($\Delta_{\mathrm{zdsv}}$ ≥ 1). After it was removed, their $\Delta_{\mathrm{zdsv}}$ dropped to 0.44.

All results with $\Delta_{\mathrm{zdsv}}$ ≥ 0.6 were true negative. This analysis suggests that the structurally identical or nearly identical proteins have $\Delta_{\mathrm{zdsv}}$ < 0.2. High similarity (i.e, the high probability for the molecules to possess the same or closely related domain composition) is signaled by $\Delta_{\mathrm{zdsv}}$ < 0.4, and it is worth investigating pairs with $\Delta_{\mathrm{zdsv}}$ < 0.5, possibly extending this search to pairs that exhibit $\Delta_{\mathrm{zdsv}}$ < 0.6.

### 2.5. Results of Retrieval—The 3D Surfer

Lastly, the 30 structures from Section 2.4 were submitted to the original 3D Surfer program [35]. It was particularly interesting since our pipeline employs the PK algorithm, while 3D Surfer relies on the voxel-based Novotni–Klein (NK) method to produce the ZC moments. Both approaches calculate ZC descriptors at moment order 20. This means that their output can be passed to Equation (1) and the other equations, analyzed in the sense of Table 1, and presented for comparison in the form of Figure 8.

At first, 3D Surfer generates the molecular surface mesh of the input structure using EDTSurf [24] (the PDB files are parsed with BioPython [69]). The mesh is then scaled down or up to fill a 64 × 64 × 64 Å grid, and discretized into its nearby voxels. ZC moments and descriptors are calculated for those voxels. The unit ball scale factor is the same as in the C++ library of Novotni and Klein, $2r_{g}$.

ZCDVs of the structures from the PDB can be downloaded from the 3D Surfer website [70,71]. Descriptors of custom models can be also produced with the offline version of 3D Surfer [72] (the EDTSurf binary must be downloaded separately from its website [73]). We calculated them in the backbone atoms (“main chain”) mode of the program.

**Figure 9:**
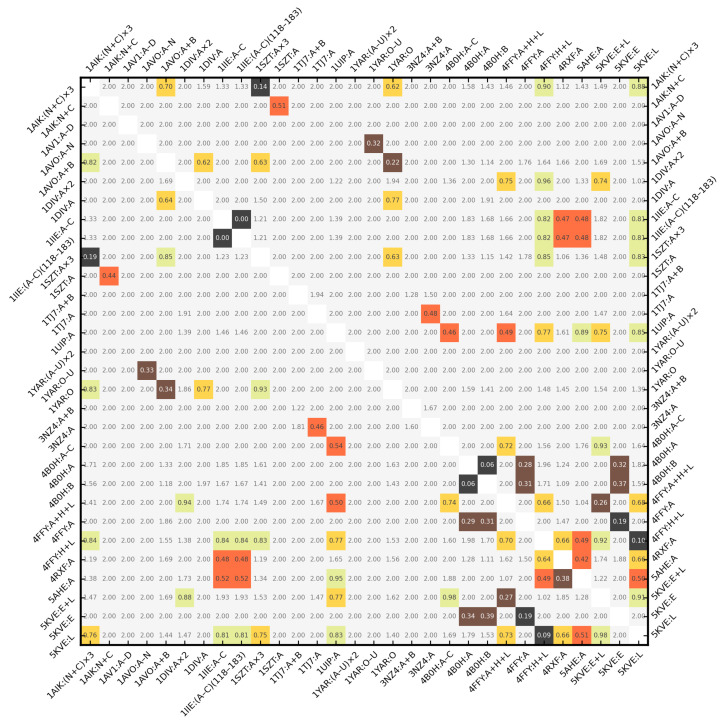
$\Delta_{\mathrm{zdsv}}$ heatmap for the 30 inputs from Table 2 (435 pairs). The results of the proposed protein structure retrieval pipeline are in the lower diagonal matrix. The results from the 3D Surfer program are in the upper diagonal matrix. See Figure 8 and Table 1 for the color scale. A scalable version of this figure is available as Figure S40 in Supplemental File S4.

Because the original 3D Surfer does not engage in structural outlier detection, to make this comparison meaningful, the outlier residues were removed a priori from all input files. After normalization, ZCDVs calculated by 3D Surfer for the 30 example structures were compared with each other using $\Delta_{z}$, $\Delta_{\mathrm{zd}}$, $\Delta_{\mathrm{zds}}$, $\Delta_{\mathrm{zdv}}$ and $\Delta_{\mathrm{zdsv}}$. Only this time the lower diagonal matrix of each heatmap was filled with the output of the PK-Zernike-based pipeline using $2r_{g}$ as the unit ball scale factor. These heatmaps are given in Supplemental File S4, in Figures S36–S40. Figure 9 is the copy of Figure S40.

The accordance of the two methods was measured with correlation coefficients (CCs). They are listed in Table 3 for the five shape distance functions. Due to sparseness of Figure 9, the comparisons were made in three value ranges: 0–2 (i.e., all heatmap cells), 0–1 (i.e., by clipping the gray cell values to 1) and below 1 (i.e., by ignoring the gray cells).

One may immediately notice the striking similarity between the two sides of Figure 9, confirmed by the very high CCs in Table 3. This means that the PK and NK algorithms lead to ZC descriptors of the molecular structures with similar interpretation and should be interchangeable within the ZC-based protein retrieval pipelines. They only need the information from the geometric features of the molecules to improve the discrimination ability of those pipelines. Because the geometric features are independent of ZC moments, and because ZC descriptors were normalized in this experiment (n.b., the ZCDs returned by 3D Surfer had values between 0 and ≈13), both pipelines were able to benefit from their combination through Equation (8) and the other equations.

With $2r_{g}$ as the unit ball scale factor, there were only a few differences between our pipeline and 3D Surfer. Most notably, 3D Surfer assigned $\Delta_{\mathrm{zdsv}}$ = 0.51 to 1AIK:(N+C) and 1SZT:A (up from 0.44), $\Delta_{\mathrm{zdsv}}$ = 0.48 to 1IIE:A–C/1IIE:(A–C)(118–183) and 5AHE (down from 0.52), $\Delta_{\mathrm{zdsv}}$ = 0.46 to 1UIP and 4B0H:A–C (down from 0.54), and $\Delta_{\mathrm{zdsv}}$ = 0.49 to 1UIP and 4FFY:A+H+L (down from 0.50). Taking $\Delta_{\mathrm{zdsv}}$ = 0.5 as the shape similarity threshold, the first pair was a false negative, the other three pairs were false positives. Both pipelines also offered $\Delta_{\mathrm{zdsv}}$ below 0.5 to 1IIE:A–C/1IIE:(A–C)(118–183) and 4RXF:A, and to 4FFY:H+L and 5AHE, resulting in two more false positives. 5AHE and 5KVE:L were close to the same status with $\Delta_{\mathrm{zdsv}}$ ≈ 0.51. It was the consequence of the higher FPR of $2r_{g}$ versus $r_{\mathrm{PCA}}$. It was most noticeable for 5AHE and 5KVE:L, for which the $\Delta_{\mathrm{zdsv}}$ difference reached 0.15 (Figure 8 vs. Figure 9). All other results in Figure 9 were correct.

We also performed a ZCD calculation time benchmark. MSMS needed 25.2 s to produce the molecular surfaces of backbone atoms of the 30 structures, and the single-threaded PKZernike class needed further 17.8 s to produce their ZC descriptors. EDTSurf took 156.2 s to generate the surfaces, while the subsequent voxelization and ZCD calculation was done by 3D Surfer in 26.2 s. The test was performed on a single CPU core.

Finally, we checked the effect of a 90% mesh decimation. The average $\Delta_{\mathrm{zdsv}}$ versus the undecimated surfaces of the example proteins was 0.031 (σ = 0.017, normalized ZCDVs). The total ZCD calculation time dropped to 3.6 s with one thread, and to only 0.7 s with six threads. The $\Delta_{\mathrm{zdsv}}$ values from Figure 8 shifted by at most ±0.02, but there was no change in their interpretation. This suggests that it is safe to gain even more speed by decimating the meshes generated by MSMS by over 50% for the PK algorithm.

## 3. Materials and Methods

### 3.1. Data

#### 3.1.1. BioZernike Validation Repository

The BioZernike validation repository [27,50] was the main benchmark for the protein structure retrieval pipeline proposed in Section 2.2. It helped with the choice of the optimal settings of this pipeline and facilitated a comparison with state-of-the-art algorithms (Section 2.3). The repository contains three test suites: “CATH”, “ECOD” and “assemblies”. Each suite is a tar.xz archive of PDB files without PDB headers.

The CATH suite was used by Guzenko et al. [27] for the training of the weights for the BioZernike shape distance function. Here, it acted as a normal test target. Its archive contains structures of 2685 domains with at most 40% of sequence identity, distributed between 151 directories named after CATH [12] superfamily codes. The number of domains per directory varies between 1 and 91, with a median of 13.

The ECOD suite acted here as another, independent benchmark. It was the primary evaluation target in Guzenko et al. The 761 domains from its archive also have at most 40% of sequence identity. They are distributed among 34 ECOD (Evolutionary Classification of Protein Domains [74]) family directories, with 2–76 files per directory (median 18). Some of the structures in this set are very small, composed of only 20 residues.

The assembly suite contains 500 biological assemblies (monomers and complexes). Guzenko et al. left a Python script [50] to generate additional four random conformations for each base structure using the ProDy package [75] with a target RMSD of 1.5 Å. The 500 assemblies are supposed to have distinct shapes, but we found that some of them, such as 1MC0_1 and 1MC0_2 [76], are identical. Hence, we cross-referenced 3D Complex v. 6.0 [14,77] to ensure that no two structures belonged to the same QS Family cluster. The deduplication of assemblies not present in the latest 3D Complex was performed with the CE algorithm [17]. The few models without sidechains were also removed. Overall, the number of base assemblies was reduced to 450, yielding a total of 2250 conformations (note: the ProDy script is very slow—it needs several hours to perturb the largest complexes). Their PDB codes and numbers are listed in Supplemental File S1, in Section S1.

The objective in the BioZernike benchmark is to (1) calculate the value of the shape distance function for all pairs of structures from a given suite, (2) select a threshold of the shape distance function below which the structures are considered similar, (3) verify the correctness of this assessment. The discrimination ability of the retrieval is measured in the receiver operating characteristic (ROC) space: false positive rate (FPR, 1–specificity) versus true positive rate (TPR, sensitivity). The true positive results are those pairs of structures that are considered similar by the retrieval system and are located in the same directory of the suite (i.e., in the same domain superfamily). Likewise, pairs of structures comprising the true negative results must belong to different directories. There are 46,572 positive and 3,556,698 negative pairs of domains in the CATH suite. The smaller ECOD domain suite has 13,603 positive and 275,577 negative pairs. With redundant models removed, the assembly suite had 4500 positive and 2,525,625 negative pairs of structures.

#### 3.1.2. The Example Protein Database

Table 4 presents the basic data of 15 structures which acted as the in-depth examples of the input and output of the protein structure retrieval pipeline from Section 2.2.

The relevant features of the example proteins are more thoroughly discussed in Supplemental File S1, in Section S2. The total number of inputs to the retrieval pipeline was actually 30 (435 pairs to compare), as some of the biological assemblies from Table 4 were split into smaller fragments. They are listed in Table 2.

1IIE, the only element of this database obtained via solution NMR, was represented by its first conformer. Multiple sequence alignment performed with Clustal Omega 1.2.4 [78] showed that there were only three pairs of structures here that exhibited identity above 20%: 1AIK and 1SZT (86.8%, HIV-1 molecule), 4FFY:A and 5KVE:E (44.5%, viral proteins), and 4FFY:H+L and 5KVE:L (34.3%, mouse antibodies).

**Table 4:** The 15 proteins used as the in-depth examples in this work.

PDB Code	Molecule	Source Organism	Chain Length	SCOP Domain(s)	Quaternary Structure	X-ray Res.	Ref.
1AIK	HIV-1 GP41 subdomain	HIV-1	37, 35	h.3.2.1	A6	2.00 Å	[79]
1AV1	Apolipoprotein A-I	*Homo sapiens*	201	h.5.1.1	A4	4.00 Å	[80]
1AVO	Proteasome PA28 activator	*Homo sapiens*	60, 140	a.24.8.1	A7B7	2.80 Å	[81]
1DIV	Ribosomal protein L9	*Bacillus* *stearothermophilus*	149	d.99.1.1, d.100.1.1	A2	2.60 Å	[82]
1IIE	HLA-DR invariant chain	*Homo sapiens*	75	a.109.1.1	A3	NMR	[83]
1SZT	HIV-1 GP41 subdomain	HIV-1	68	h.3.2.1	A6	2.40 Å	[84]
1TJ7	Argininosuccinate lyase	*Escherichia coli*	457	a.127.1.1	A2	2.44 Å	[85]
1UIP	Adenosine deaminase	*Mus musculus*	349	c.1.9.1	A1	2.40 Å	[86]
1YAR	Proteasome PA26 activator, Proteasome 20S core chamber	*Thermoplasma* *acidophilum*	237, 233, 217	a.24.8.1, d.153.1.4	A28B14	1.90 Å	[87]
3NZ4	Phenylalanine aminomutase	*Taxus canadensis*	696	a.127.1.0	A2	2.38 Å	[88]
4B0H	dUTPase YncF	*Bacillus subtilis*	144	b.85.4.0	A3	1.18 Å	[89]
4FFY	Dengue virus specific antibody E111	DENV-1, *Mus musculus*	111, 130, 126	b.1.18.4, b.1.1.1	A1B1C1	2.50 Å	[90]
4RXF	Fructose-6-phosphate aldolase	*Escherichia coli*	226	c.1.10.1	A10	2.40 Å	[91]
5AHE	HisA enzyme	*Salmonella enterica*	253	c.1.2.0	A1	1.70 Å	[92]
5KVE	Zika virus specific antibody ZV-48	ZIKV, *Mus musculus*	110, 245	b.1.18.4, b.1.1.0	A1B1	1.70 Å	[93]

The Quaternary structure column denotes the stoichiometry of the representative (i.e., first) biological assembly.

### 3.2. Algorithms

#### 3.2.1. The Detection of Outlier Residues

Our structural outlier detection algorithm follows the principles established in [22] and optimized in [23,94]. Its purpose is to find residues that significantly protrude from the main body of the molecule, such as the chain termini in 1IIE, 1YAR and 4B0H. The input is a cloud of points, here—effective atoms. Effective atoms provide a simple, binary variable: a residue is or is not a structural outlier as a whole. We call the non-outliers the guides—they “guide” the EP algorithm [22,23] towards more compact representations of the proteins via minimum volume enclosing ellipsoids [61]. The details are laid out in Supplemental File S1, in Section S3. A ready-to-use Python code is provided there.

This algorithm is said to be PCA-based because its first steps are identical to the first steps of principal component analysis [51] (it also works in any number of dimensions, although no reduction in dimensionality is performed here). Briefly, the effective atoms are centered at the origin and aligned with the principal axes of the coordinate system. An optimal rotation matrix is determined via the singular value decomposition (SVD). This factorization is the basis of the PCA and of Kabsch’s algorithm [52,53]. It maximizes the variance of the effective atoms in each dimension and zeroes their cross-covariance. An axis-aligned confidence ellipsoid is then drawn around the rotated effective atoms. The multiplication factor of its radii, *s*, is taken from the χ^2^ distribution with probability *p* = 0.9 (a user-controlled parameter). Effective atoms outside the confidence ellipsoid are outliers. The internal, guide effective atoms are then realigned with SVD and split again into guides and outliers by another confidence ellipsoid. This tandem loops at most *r* times (default: 3). The loop terminates early if no new outliers are captured or when there are no guides left. With a fixed, low number of dimensions (e.g., 3 in case of the proteins), the complexity of this algorithm is O(rn), where *n* is the number of input points.

#### 3.2.2. Calculation of Molecular Surfaces

Molecular surfaces of the proteins were solved with MSMS v. 2.6.1 [62] invoked with –free_vertices, –all_components and –probe_radius 1.4 arguments. The first two switches are essential. Without them, MSMS may produce the surface only for a portion of the input (e.g., for one chain in the complex). The program also has a hard limit on the number of components, so if it failed to converge with –all_components (which happened to 1YAR); it was automatically re-run without this argument.

The values of van der Waals radii were taken from the NACCESS standard residue library [95]. Their definition file was copied from the repository of dr-sasa [96,97]. Modified residues inherited the radii of their standard parent residues; for instance, the C_α_ atom from selenomethionine had the same vdW radius as the C_α_ atom from methionine.

#### 3.2.3. Decimation of Molecular Surfaces

The meshes of molecular surfaces produced by MSMS in this experiment were decimated (i.e., simplified, with a reduced number of vertices and facets) through PyVista [66], a streamlined Python interface to the Visualization Toolkit (VTK) [67].

Two mesh decimation filters are provided by the VTK library: vtkQuadricDecimation and vtkDecimatePro. The first algorithm is based on the quadric error metrics by Garland and Heckbert [63], the other algorithm follows the work of Schroeder and colleagues [64]. vtkQuadricDecimation aims to produce equilateral facets of similar area (i.e., uniform), while vtkDecimatePro allows the facets to adapt their shape and size to the curvature of the mesh. If the reduction factor is high (e.g., 90% and above), the PreserveTopology parameter of vtkDecimatePro should be enabled to prevent holes from appearing in the surface.

Here, the vtkQuadricDecimation filter was applied with a reduction factor of 0.5 (i.e., 50%). A ready-to-use Python code is provided in Supplemental File S1 in Section S4.

#### 3.2.4. The Pozo–Koehl (PK) Algorithm

The algorithm of Pozo et al. [31] calculates Zernike–Canterakis moments directly from the geometric moments of unstructured triangular surface meshes scaled down to fit inside the unit ball. Computer models of molecular surfaces of proteins, such as those produced by MSMS, naturally lend themselves as its input.

There are a few variants of the algorithm—one is exact, the other is approximated. The geometric moments can also be volume-like or surface-like. Here, we implemented and utilized the variant that most accurately represents the input mesh: exact and volume-like. Its steps and necessary equations are given in Supplemental File S1, in Section S5.

“Exact” means that “given an object defined exactly by a particular triangle mesh, the algorithm provides the exact geometric moments of this object” [31]. “Volume-like” means that the geometric moments are obtained from the sum of integrals over the volume of the oriented tetrahedra spanned between the origin and the vertices that constitute each facet of the mesh. This sum expresses the integral over the whole object. The tetrahedra are said to be oriented, because depending on the orientation of the facets, their volume can be positive or negative. To make it work, the three vertices of each facet need to be listed in the counter-clockwise direction when looking at the mesh from its exterior. The molecular surfaces produced by MSMS and decimated by VTK conform to this requirement.

The complexity of the geometric moment calculation step is O($fN^{6}$) in the original algorithm and O($fN^{3}$) with Koehl’s optimizations [34], where *f* is the number of facets and *N* is the moment order. The next, final step involves the calculation of ZC moments from geometric moments. Pozo et al. offered a time-efficient solution to this problem that involves loops of depth 4 over 5 arrays (four intermediates plus one). This is faster than the single loop of depth 6 in the paper of Novotni and Klein [32] (n.b., the NK algorithm obtains geometric moments in O($vN^{3}$) time, where *v* is the number of voxels in the grid). Because the number of ZC moments depends only on the order (i.e., *N*), the complexity of this step is independent of the shape and size of the input object. Both algorithms spend nearly all of their time on geometric moment calculation.

ZC moments are not invariant to the rotations of the input shape. To attain the rotation invariance needed for fast, quantitative analysis of the similarity of objects they encode, Novotni and Klein popularized the formula of the so-called 3D Zernike descriptors, 3DZDs [32]. The (complex) ZC moments are collected in vectors. The (real) norms of those vectors are 3DZDs. They act as the low-resolution representation of ZC moments. Here, following Pozo et al. and Ljung and André [20], we call them Zernike–Canterakis descriptors (ZCDs). We also call the vector of the ZCDs the Zernike–Canterakis descriptor vector [58] (ZCDV). It is calculated in O($N^{3}$) time. Again, this time only depends on the moment order. The difference (i.e., delta) between the ZCDVs is the basis of our shape distance functions, hence the choice of the Δ symbol in Equation (1) and the other equations.

### 3.3. Tools

The multiple sequence alignment was facilitated by Clustal Omega [78]. Molecular surface meshes were solved with MSMS [62]. Images of the proteins were rendered with PyMOL [98] (cartoon style) and PyVista [66] (surface style). Charts were plotted with the Matplotlib library [99]. The experiments were performed with the help of NumPy [48], SciPy [49], Numba [47], ProDy [75] and BioPython [69] packages. BioPython was the dependency of 3D Surfer [35] and ProDy; we used our in-house PDB file parser.

The code of fast Python implementation of the PK algorithm in PK-Zernike library is available at https://codeberg.org/mbanach/pkzernike. Some portions of this code were derived from the shapes.zernike package from Mindboggle library [46,59], most notably the optimal indexing that avoids the iteration through the entire arrays.

All time benchmarks were carried out on a PC with a 6 core/12 thread 4.2 GHz AMD Ryzen CPU, 16 GB of dual-channel DDR4, running Linux 5 and Python 3.9.

## 4. Conclusions

In this paper, we report on the progress toward the increased availability of fast and well-documented calculation of Zernike–Canterakis moments and descriptors in Python. We created the small PK-Zernike library which, to the best of our knowledge, is currently the fastest Python implementation of the Pozo–Koehl algorithm (PK) [31,34]. In contrast to the popular approach by Novotni and Klein [32], the PK algorithm produces ZC moments from the geometric moments obtained directly from the unstructured surface meshes of 3D models. The shapes are represented exactly, no voxelization is needed.

The PK-Zernike library relies on Numba’s just-in-time compiler to optimized machine code. Thus, it contains only Python code (with calls to NumPy’s math and array functions) that is easy to understand and apply, but, once compiled, can process the same mesh over 200–300 times faster at moment order 20. Owing to PK-Zernike, a consumer-grade PC can process about 50,000 facets per second. Calculation time can be further reduced via the decimation of the mesh (by up to 90%) and with parallel, thread-based execution. A typical protein (≈500 aa) is then processed in less than a few dozen of milliseconds.

One should be aware that the PK-Zernike library is generic. It accepts its input in the form of vertex and facet NumPy arrays. These arrays can be passed by the application, or loaded from the mesh file specified in the command line: python3 –m pkzernike path/to/the/mesh.obj. A rudimentary Wavefront OBJ format reader/writer is built in the library. Other mesh formats, such as PLY or STL, are supported through PyVista (a soft dependency). PyVista also exposes VTK’s mesh decimation filters to Python.

ZC moments lie at the foundation of state-of-the-art protein structure retrieval systems [27,35]. The shape of a molecule is encoded by a feature vector with a fixed length that can be quickly compared with feature vectors of the other molecules. This facilitates real-time (e.g., sub-second) searches for similarly shaped proteins in the entire database. Such functionality is offered by the web interface of the PDB since 2020 [2].

Because the molecular surfaces of proteins produced by programs like MSMS are proper 3D meshes, they can readily become input data for the PK algorithm. However, it seems that nobody has utilized this fact in a fully-featured protein structure retrieval. Hence, to assess the usefulness of the PK algorithm in this area, we made it the pivotal element of a novel protein structure retrieval pipeline. This relatively simple workflow, based only on MSMS, NumPy, SciPy, Numba, PyVista/VTK and PK-Zernike (and our in-house PDB format parser), utilizes ZC descriptors and, following the footsteps of BioZernike [27], three geometric features: the distribution of distances of residues to the origin, the size of the molecule and the volume of its mesh. We also devised several shape distance functions capable of joining these geometric features with ZC descriptors.

The discrimination ability of the proposed pipeline was checked by three suites from the BioZernike validation repository [50]. There, it achieved the following areas under the ROC curves: ≈0.96 for the domains (both CATH and ECOD), and up 0.997 for the biological assemblies. These values are close to the AUROCs reported for BioZernike: 0.97 and 1.0, respectively. This experiment also unveiled the optimal default parameters of our method, namely the calculation of molecular surfaces for the backbones of the proteins (N, C_α_, C and O atoms), either $r_{\mathrm{PCA}}$ (lower TPR and FPR) or $2r_{g}$ (higher TPR and FPR) as the unit ball scale factor, and $\Delta_{\mathrm{zdsv}}$ as the primary shape distance metric.

The high discrimination ability of the proposed retrieval pipeline would not be reached without the PCA-based outlier residue detection subroutine borrowed from our other algorithm, the Ellipsoid Profile (EP) [22,23]. The purpose of this subroutine is to eliminate the exposed fragments of the molecules (e.g., disordered chain termini) that contribute little to its volume but can skew the metrics that rely on its shape or position. Here, it allowed the TPR of the retrieval to increase a lot at the expense of only a small increase in the FPR. The detection is fast and simple. It may be disabled to reduce the FPR, for example, to find similar structures in which the crucial shape information in stored in the outliers.

The analysis of the example protein set showed that the proposed pipeline is capable of returning correct scores of their structural similarity, additionally reaching $\Delta_{\mathrm{zdsv}}$ correlation coefficients over 0.95 with the 3D Surfer program [35]. This confirms that ZC descriptors of the molecular surface meshes obtained via the exact, volume-like PK algorithm are at least as useful as ZC descriptors of the voxelized protein representations. Thus, it confirms the usefulness of the PK algorithm in structural bioinformatics.

The algorithms of Pozo et al. and of Novotni and Klein begin to suffer from numerical instabilities above moment order 35 when the calculation is performed with the standard double precision arithmetic. This phenomenon, caused by the large cancellations in the geometric-to-Zernike moment conversion, is explained in depth by Houdayer and Koehl [40]. The same authors proposed [40] an alternative three-dimensional Zernike algorithm based on quadrature rules that circumvents the main issue by calculating the Zernike moments directly from the facets of the mesh, without the geometric moments intermediate. The finite-precision variant of their approach is time-wise on par with the PK algorithm, but stable at moment order 300 and beyond. The HK algorithm is a mesh-based counterpart of the work of Deng and Gwo [100], who earlier adopted the direct calculation idea to the voxel-based shape representation with similar success. While moment orders up to 35 may be sufficient for structural bioinformatics, we believe these new algorithms are going to make an impact on the general field of 3D shape retrieval and reconstruction.

It should also be mentioned that Zernike moments are not the only noise-resistant method of encoding the shape of molecular structures. In particular, van Kempen and colleagues recently published an article about Foldseek [101]. This robust superposition algorithm performs an “inverse translation”; it takes the geometric features of the proteins and discretizes them into sequences of a custom structural alphabet. The local alignment of those sequences results in a local alignment of the structures. In this sense, Foldseek falls into the same category as CE and the other superposition methods [16,17,18,19,20]. However, its sequence-based protocol organically extends to the search for similarly shaped proteins in the supported databases (e.g., in the PDB). While these searches are slower than the comparisons of ZC descriptors, they can deliver accurate partial matches in a matter of seconds [102], unlike ZCDs, which essentially facilitate a global comparison of the shapes inside the unit ball, and can be skewed by the structural outliers. Conversely, ZCDs are free from the constraints of the logical organization of the molecules (e.g., sequence order or chain IDs). Foldseek does global alignment with an accelerated TM-align [19].

The reader may also be interested in a recent paper by Koehl and Orland [103] on the subject of partial 3D shape matching. To cite its abstract, “A new algorithm is presented to compute nonrigid, possibly partial comparisons of shapes defined by unstructured triangulations of their surfaces. (…) Its goal is to define a possibly partial correspondence between the vertices of the two triangulations, with a cost associated with this correspondence that can serve as a measure of the similarity of the two shapes. To find this correspondence, the vertices in each triangulation are characterized by a signature vector of features.” It would be very interesting to see if this method can be applied to protein content.

## Figures and Tables

**Table 1:** The proposed $\Delta_{z}$-based shape similarity scale for normalized ZC descriptors.

Tier	Lower Bound (Included)	Upper Bound (Excluded)	Shape Similarity Interpretation	Color in the Figures
1	0.0	0.2	very high similarity	black
2	0.2	0.4	high similarity	brown
3	0.4	0.6	medium similarity	orange
4	0.6	0.8	low similarity	yellow
5	0.8	1.0	very low similarity	green
6	1.0	2.0	no similarity	gray

Color names in the last column refer to the colors of the circle markers and the backgrounds of the heatmap cells in figures below this table and in the Supplemental Files S2–S4.

**Table 2:** 30 fragments of example proteins used for the in-depth analysis of the proposed protein structure retrieval pipeline and for its comparison with the 3D Surfer program in Section 2.5.

Input Structure		Effective Atoms		Molecular Surface Mesh			Eff. Atom. St. Dev.			Scale Factor		Runtime (s)	
PDB Code	Selection	Total	Guide	Vertices	Facets	Vol.	σ_x_	σ_y_	σ_z_	$r_{\max}$	$r_{\mathrm{PCA}}$	**×1**	**×6**
1AIK	(N+C)×3	210	195	3589	7154	9.5	14.3	6.9	6.9	30.9	39.9	0.140	0.026
1AIK	N+C	70	60	1124	2240	2.9	13.8	4.6	3.0	32.2	38.5	0.044	0.011
1AV1	A−D	804	804	15,221	30,426	38.2	39.1	27.5	14.1	68.9	109.4	0.598	0.104
1AVO	A−N	1400	1400	27,236	54,501	66.7	21.0	21.0	17.2	51.0	58.7	1.064	0.184
1AVO	A+B	200	167	3298	6600	8.0	18.0	7.0	5.1	38.3	50.3	0.129	0.025
1DIV	A×2	298	244	5736	11,520	12.0	17.5	12.7	9.0	46.4	49.0	0.227	0.041
1DIV	A	149	141	3371	6774	7.0	23.2	7.2	4.3	44.9	64.8	0.133	0.026
1IIE	A−C	225	198	3989	7966	9.4	9.8	9.8	7.2	23.3	27.5	0.158	0.028
1IIE	(A−C)(118−183)	198	198	3989	7966	9.4	9.8	9.8	7.2	23.3	27.5	0.157	0.029
1SZT	A×3	204	186	3586	7148	9.1	14.1	7.0	7.0	29.9	39.3	0.142	0.027
1SZT	A	68	61	1207	2410	3.0	14.3	5.8	2.4	30.5	40.0	0.048	0.011
1TJ7	A+B	906	696	15,288	30,727	34.6	20.5	16.4	10.2	47.2	57.4	0.605	0.104
1TJ7	A	455	412	9137	18,378	20.5	27.1	8.8	7.8	57.0	75.6	0.364	0.064
1UIP	A	349	342	8057	16,247	17.2	12.4	10.3	9.1	30.7	34.7	0.318	0.057
1YAR	(A−U)×2	8988	8988	210,135	422,129	445.5	73.0	25.4	25.4	149.3	203.9	8.256	1.404
1YAR	O−U	1526	1477	30,753	61,790	72.6	20.1	20.1	17.3	49.0	56.2	1.222	0.208
1YAR	O	218	192	4008	8050	9.4	18.8	7.1	5.2	46.4	52.4	0.158	0.030
3NZ4	A+B	1310	1182	27,253	55,082	58.5	27.4	18.7	9.0	71.4	76.5	1.084	0.185
3NZ4	A	654	606	14,039	28,345	30.3	29.6	9.1	8.3	70.6	82.8	0.556	0.097
4B0H	A−C	416	407	10,590	21,272	19.9	12.9	12.0	10.6	32.8	35.9	0.420	0.073
4B0H	A	130	108	2842	5717	5.4	10.8	6.7	5.2	23.0	30.1	0.113	0.021
4B0H	B	143	111	2927	5883	5.5	10.8	7.0	5.3	24.6	30.3	0.117	0.023
4FFY	A+H+L	328	312	8557	17,181	15.6	14.6	10.9	8.0	38.1	40.9	0.341	0.060
4FFY	A	97	96	2663	5363	4.7	9.9	6.5	5.2	23.9	27.7	0.105	0.021
4FFY	H+L	231	220	5959	11,956	11.0	12.8	8.4	7.9	29.8	35.7	0.238	0.041
4RXF	A	220	190	4576	9219	9.6	10.5	9.8	5.9	30.3	29.3	0.183	0.033
5AHE	A	231	227	5636	11,335	11.4	11.0	10.1	7.2	26.3	30.6	0.226	0.040
5KVE	E+L	331	314	8530	17,137	15.8	16.1	11.4	7.1	38.1	44.9	0.337	0.061
5KVE	E	101	94	2514	5049	4.7	10.1	5.8	5.1	22.7	28.1	0.100	0.020
5KVE	L	230	215	5844	11,742	10.9	12.9	8.4	7.6	29.3	36.0	0.231	0.043

Underline in the Selection column means that the selected fragment corresponds to the entirety of the first (i.e., representative) biological assembly from a given PDB structure. The *×n* suffix informs that the chains were copied and transformed *n* times to recreate this biological assembly (REMARK 350). The Effective atoms column contains the number of effective atoms in the selection and in its guide subset (i.e., after the removal of the outlier residues, Section 2.2.3). The Molecular surface mesh column shows the number of vertices, facets and the volume (divided by 1000) of the backbone atom molecular surface mesh (Section 2.2.4) after its decimation by 50% (Section 2.2.5). $\sigma_{x}$, $\sigma_{y}$ and $\sigma_{z}$ are post-alignment effective atom standard deviations in each dimension (Section 2.2.6), needed for Equation (4). The $r_{\mathrm{PCA}}$ value in the Scale factor column placed the vertices of the mesh inside the unit sphere; $r_{\max}$—the longest vertex distance to the origin—is provided for reference (it was used for 4RXF:A, Section 2.2.7). The numbers in the last column are the approximate mesh processing times by the fully compiled PKZernike class (Section 2.1); they do not include the time needed to parse the PDB file, or to calculate the molecular surface. The ×1 and ×6 labels in this column refer to the number of worker threads.

**Table 3:** Correlation coefficients of the values of the shape distance functions based on the ZCDs calculated by the PK-Zernike library and by the 3D Surfer program for the structures from Table 2.

Distance Function	[0…2]	[0…1]	[0…1)	Figure
$\Delta_{z}$	0.907	0.907	0.905	Figure S36
$\Delta_{\mathrm{zd}}$	0.957	0.947	0.926	Figure S37
$\Delta_{\mathrm{zdv}}$	0.981	0.978	0.952	Figure S38
$\Delta_{\mathrm{zds}}$	0.975	0.982	0.965	Figure S39
$\Delta_{\mathrm{zdsv}}$	0.990	0.985	0.957	Figure S40

[0…2] means that the correlation was calculated with no changes to the values of the shape distance function. [0…1] means that values above 1 were clipped to 1. [0…1) means that values equal or higher than 1 were ignored. The figures referenced in the last column can be found in Supplemental File S4.

## Data Availability

The PK-Zernike library is available at https://codeberg.org/mbanach/pkzernike. Online access to the Ellipsoid Profile algorithm, the Fuzzy Oil Drop algorithm and related bioinformatics tools is possible at the http://fod.cm-uj.krakow.pl web server.

## Supplementary Materials

The following supporting information can be downloaded at: https://www.mdpi.com/article/10.3390/molecules29010052/s1. Supplemental File S1 (Figures S1–S14)—3D renders and superpositions of the proteins from Table 4; Supplemental File S2 (Figures S15–S30)—ROC curves measuring the discrimination ability of the proposed protein structure retrieval pipeline in the BioZernike validation suites (see Figure 7 for description); Supplemental File S3 (Figures S31–S35)—$\Delta_{z}$, $\Delta_{\mathrm{zd}}$, $\Delta_{\mathrm{zds}}$, $\Delta_{\mathrm{zdv}}$ and $\Delta_{\mathrm{zdsv}}$ heatmaps for the structures from Table 2 (see Figure 8 for description); Supplemental File S4 (Figures S36–S40)—$\Delta_{z}$, $\Delta_{\mathrm{zd}}$, $\Delta_{\mathrm{zds}}$, $\Delta_{\mathrm{zdv}}$ and $\Delta_{\mathrm{zdsv}}$ heatmaps for the structures from Table 2 (the comparison with 3D Surfer, see Figure 9 for description).

## Abbreviations

The following abbreviations are used in this manuscript:

$\Delta_{z}$	Equation (1)
$\Delta_{z[\mathrm{dsv}]}$	Equations (2)–(8)
3DZD	3D Zernike Descriptor (→ZCD)
AUROC	Area Under the ROC curve
EP	Ellipsoid Profile algorithm
FPR	False Positive Rate
JIT	Just-In-Time compilation
MODRES	MODified RESidues records in a PDB file
MSMS	Michel Sanner’s Molecular Surface program
NK	Novotni–Klein algorithm
PCA	Principal Component Analysis
PDB	Protein Data Bank
PK	Pozo–Koehl algorithm
RMSD	Root Mean Square Deviation
ROC	Receiver Operating Characteristic
TPR	True Positive Rate
ZC	Zernike–Canterakis
ZCD	Zernike–Canterakis Descriptor
ZCDV	Zernike–Canterakis Descriptor Vector

## References

[1] Berman H.M., Westbrook J., Feng Z., Gilliland G., Bhat T.N., Weissig H., Shindyalov I.N., Bourne P.E. (2000). The Protein Data Bank. Nucleic Acids Res..

[2] Burley S.K., Bhikadiya C., Bi C., Bittrich S., Chen L., Crichlow G.V., Christie C.H., Dalenberg K., Di Costanzo L., Duarte J.M. (2020). RCSB Protein Data Bank: Powerful new tools for exploring 3D structures of biological macromolecules for basic and applied research and education in fundamental biology, biomedicine, biotechnology, bioengineering and energy sciences. Nucleic Acids Res..

[3] PDB Statistics. https://www.rcsb.org/stats/summary.

[4] Bateman A., Martin M.J., Orchard S., Magrane M., Ahmad S., Alpi E., Bowler-Barnett E.H., Britto R., Bye-A-Jee H., Cukura A. (2022). UniProt: The Universal Protein Knowledgebase in 2023. Nucleic Acids Res..

[5] Senior A.W., Evans R., Jumper J., Kirkpatrick J., Sifre L., Green T., Qin C., Žídek A., Nelson A.W.R., Bridgland A. (2020). Improved protein structure prediction using potentials from deep learning. Nature.

[6] Jumper J., Evans R., Pritzel A., Green T., Figurnov M., Ronneberger O., Tunyasuvunakool K., Bates R., Žídek A., Potapenko A. (2021). Highly accurate protein structure prediction with AlphaFold. Nature.

[7] Pereira J., Simpkin A.J., Hartmann M.D., Rigden D.J., Keegan R.M., Lupas A.N. (2021). High-accuracy protein structure prediction in CASP14. Proteins Struct. Funct. Bioinform..

[8] Hou J., Jun S.R., Zhang C., Kim S.H. (2005). Global mapping of the protein structure space and application in structure-based inference of protein function. Proc. Natl. Acad. Sci. USA.

[9] Ptitsyn O. (1991). How does protein synthesis give rise to the 3D-structure?. FEBS Lett..

[10] Banach M., Prudhomme N., Carpentier M., Duprat E., Papandreou N., Kalinowska B., Chomilier J., Roterman I. (2015). Contribution to the Prediction of the Fold Code: Application to Immunoglobulin and Flavodoxin Cases. PLoS ONE.

[11] Rost B. (1999). Twilight zone of protein sequence alignments. Protein Eng. Des. Sel..

[12] Sillitoe I., Bordin N., Dawson N., Waman V.P., Ashford P., Scholes H.M., Pang C.S.M., Woodridge L., Rauer C., Sen N. (2020). CATH: Increased structural coverage of functional space. Nucleic Acids Res..

[13] Chandonia J.M., Guan L., Lin S., Yu C., Fox N., Brenner S. (2021). SCOPe: Improvements to the structural classification of proteins—Extended database to facilitate variant interpretation and machine learning. Nucleic Acids Res..

[14] Levy E.D., Pereira-Leal J.B., Chothia C., Teichmann S.A. (2006). 3D Complex: A Structural Classification of Protein Complexes. PLoS Comput. Biol..

[15] Chen J., Guo M., Wang X., Liu B. (2016). A comprehensive review and comparison of different computational methods for protein remote homology detection. Brief. Bioinform..

[16] Holm L., Sander C. (1993). Protein Structure Comparison by Alignment of Distance Matrices. J. Mol. Biol..

[17] Shindyalov I.N., Bourne P.E. (1998). Protein structure alignment by incremental combinatorial extension (CE) of the optimal path. Protein Eng. Des. Sel..

[18] Ye Y., Godzik A. (2003). Flexible structure alignment by chaining aligned fragment pairs allowing twists. Bioinformatics.

[19] Zhang Y. (2005). TM-align: A protein structure alignment algorithm based on the TM-score. Nucleic Acids Res..

[20] Ljung F., André I. (2021). ZEAL: Protein structure alignment based on shape similarity. Bioinformatics.

[21] Connolly M.L. (1983). Analytical molecular surface calculation. J. Appl. Crystallogr..

[22] Banach M. (2021). Assessment of Globularity of Protein Structures via Minimum Volume Ellipsoids and Voxel-Based Atom Representation. Crystals.

[23] Banach M. (2023). Improved Assessment of Globularity of Protein Structures and the Ellipsoid Profile of the Biological Assemblies from the PDB. Biomolecules.

[24] Xu D., Zhang Y. (2009). Generating Triangulated Macromolecular Surfaces by Euclidean Distance Transform. PLoS ONE.

[25] Serre L. (2002). Crystal structure of the Lactococcus lactis formamidopyrimidine-DNA glycosylase bound to an abasic site analogue-containing DNA. EMBO J..

[26] Tangelder J., Veltkamp R. A survey of content based 3D shape retrieval methods. Proceedings of the Shape Modeling Applications.

[27] Guzenko D., Burley S.K., Duarte J.M. (2020). Real time structural search of the Protein Data Bank. PLoS Comput. Biol..

[28] Niu K., Tian C. (2022). Zernike polynomials and their applications. J. Opt..

[29] Zernike v.F. (1934). Beugungstheorie des schneidenver-fahrens und seiner verbesserten form, der phasenkontrastmethode. Physica.

[30] Canterakis N. 3D Zernike moments and Zernike affine invariants for 3D image analysis and recognition. Proceedings of the 11th Scandinavian Conference on Image Analysis.

[31] Pozo J.M., Villa-Uriol M.C., Frangi A.F. (2011). Efficient 3D Geometric and Zernike Moments Computation from Unstructured Surface Meshes. IEEE Trans. Pattern Anal. Mach. Intell..

[32] Novotni M., Klein R. (2004). Shape retrieval using 3D Zernike descriptors. Comput. Aided Des..

[33] Prokop R.J., Reeves A.P. (1992). A survey of moment-based techniques for unoccluded object representation and recognition. CVGIP Graph. Model. Image Process..

[34] Koehl P. (2012). Fast Recursive Computation of 3D Geometric Moments from Surface Meshes. IEEE Trans. Pattern Anal. Mach. Intell..

[35] Sael L., Li B., La D., Fang Y., Ramani K., Rustamov R., Kihara D. (2008). Fast protein tertiary structure retrieval based on global surface shape similarity. Proteins Struct. Funct. Bioinform..

[36] Han X., Sit A., Christoffer C., Chen S., Kihara D. (2019). A global map of the protein shape universe. PLoS Comput. Biol..

[37] Aderinwale T., Bharadwaj V., Christoffer C., Terashi G., Zhang Z., Jahandideh R., Kagaya Y., Kihara D. (2022). Real-time structure search and structure classification for AlphaFold protein models. Commun. Biol..

[38] Langenfeld F., Peng Y., Lai Y.K., Rosin P.L., Aderinwale T., Terashi G., Christoffer C., Kihara D., Benhabiles H., Hammoudi K. (2020). SHREC 2020: Multi-domain protein shape retrieval challenge. Comput. Graph..

[39] Langenfeld F., Aderinwale T., Christoffer C., Shin W.H., Terashi G., Wang X., Kihara D., Benhabiles H., Hammoudi K., Cabani A. (2022). Surface-based protein domains retrieval methods from a SHREC2021 challenge. J. Mol. Graph. Model..

[40] Houdayer J., Koehl P. (2022). Stable Evaluation of 3D Zernike Moments for Surface Meshes. Algorithms.

[41] BioZernike Repository. https://github.com/biocryst/biozernike.

[42] Oliphant T.E. (2007). Python for Scientific Computing. Comput. Sci. Eng..

[43] AlphaFold Repository. https://github.com/google-deepmind/alphafold.

[44] Python Package Index Website. https://pypi.org.

[45] Stack Exchange: Computing 3D Zernike Moments on 3D Point Clouds. https://math.stackexchange.com/questions/3940296/computing-3d-zernike-moments-on-3d-point-clouds.

[46] Klein A., Ghosh S.S., Bao F.S., Giard J., Häme Y., Stavsky E., Lee N., Rossa B., Reuter M., Chaibub Neto E. (2017). Mindboggling morphometry of human brains. PLoS Comput. Biol..

[47] Lam S.K., Pitrou A., Seibert S. (2015). Numba: A LLVM-based Python JIT compiler. Proceedings of the Second Workshop on the LLVM Compiler Infrastructure in HPC.

[48] Harris C.R., Millman K.J., van der Walt S.J., Gommers R., Virtanen P., Cournapeau D., Wieser E., Taylor J., Berg S., Smith N.J. (2020). Array programming with NumPy. Nature.

[49] Virtanen P., Gommers R., Oliphant T.E., Haberland M., Reddy T., Cournapeau D., Burovski E., Peterson P., Weckesser W., Bright J. (2020). SciPy 1.0: Fundamental algorithms for scientific computing in Python. Nat. Methods.

[50] BioZernike Validation Repository. https://github.com/rcsb/biozernike-validation.

[51] Jolliffe I. (2002). Principal Component Analysis.

[52] Kabsch W. (1976). A solution for the best rotation to relate two sets of vectors. Acta Crystallogr. Sect. A.

[53] Kabsch W. (1978). A discussion of the solution for the best rotation to relate two sets of vectors. Acta Crystallogr. Sect. A.

[54] Xu Q., Dunbrack R.L. (2019). Principles and characteristics of biological assemblies in experimentally determined protein structures. Curr. Opin. Struct. Biol..

[55] Elez K., Bonvin A.M.J.J., Vangone A. (2020). Biological vs. Crystallographic Protein Interfaces: An Overview of Computational Approaches for Their Classification. Crystals.

[56] Konieczny L., Roterman I. (2020). Description of the fuzzy oil drop model. From Globular Proteins to Amyloids.

[57] Banach M., Chomilier J., Roterman I. (2021). Contribution to the Understanding of Protein-Protein Interface and Ligand Binding Site Based on Hydrophobicity Distribution—Application to Ferredoxin I and II Cases. Appl. Sci..

[58] Callahan P.G., De Graef M. (2011). Precipitate shape fitting and reconstruction by means of 3D Zernike functions. Model. Simul. Mater. Sci. Eng..

[59] Mindboggle Repository. https://github.com/nipy/mindboggle.

[60] Mikhno A., Nuevo P.M., Devanand D.P., Parsey R.V., Laine A.F. Multimodal classification of Dementia using functional data, anatomical features and 3D invariant shape descriptors. Proceedings of the 9th IEEE International Symposium on Biomedical Imaging (ISBI).

[61] Khachiyan L.G. (1996). Rounding of Polytopes in the Real Number Model of Computation. Math. Oper. Res..

[62] Sanner M.F., Olson A.J., Spehner J.C. (1996). Reduced surface: An efficient way to compute molecular surfaces. Biopolymers.

[63] Garland M., Heckbert P.S. (1997). Surface simplification using quadric error metrics. Proceedings of the 24th Annual Conference on Computer Graphics and Interactive Techniques.

[64] Schroeder W.J., Zarge J.A., Lorensen W.E. (1992). Decimation of triangle meshes. Proceedings of the 19th Annual Conference on Computer Graphics and Interactive Techniques.

[65] Savitzky A., Golay M.J.E. (1964). Smoothing and Differentiation of Data by Simplified Least Squares Procedures. Anal. Chem..

[66] Sullivan C., Kaszynski A. (2019). PyVista: 3D plotting and mesh analysis through a streamlined interface for the Visualization Toolkit (VTK). J. Open Source Softw..

[67] Schroeder W.J., Martin K.M. (2005). The Visualization Toolkit. Visualization Handbook.

[68] Suzuki H., Kawabata T., Nakamura H. (2015). Omokage search: Shape similarity search service for biomolecular structures in both the PDB and EMDB. Bioinformatics.

[69] Cock P.J.A., Antao T., Chang J.T., Chapman B.A., Cox C.J., Dalke A., Friedberg I., Hamelryck T., Kauff F., Wilczynski B. (2009). Biopython: Freely available Python tools for computational molecular biology and bioinformatics. Bioinformatics.

[70] La D., Esquivel-Rodríguez J., Venkatraman V., Li B., Sael L., Ueng S., Ahrendt S., Kihara D. (2009). 3D-SURFER: Software for high-throughput protein surface comparison and analysis. Bioinformatics.

[71] 3D Surfer Website. https://kiharalab.org/3d-surfer.

[72] 3D Surfer Repository. https://github.com/kiharalab/3d-af_surfer.

[73] EDTSurf Website. https://zhanggroup.org/EDTSurf.

[74] Cheng H., Schaeffer R.D., Liao Y., Kinch L.N., Pei J., Shi S., Kim B.H., Grishin N.V. (2014). ECOD: An Evolutionary Classification of Protein Domains. PLoS Comput. Biol..

[75] Bakan A., Meireles L.M., Bahar I. (2011). ProDy: Protein Dynamics Inferred from Theory and Experiments. Bioinformatics.

[76] Martinez S.E., Wu A.Y., Glavas N.A., Tang X.B., Turley S., Hol W.G.J., Beavo J.A. (2002). The two GAF domains in phosphodiesterase 2A have distinct roles in dimerization and in cGMP binding. Proc. Natl. Acad. Sci. USA.

[77] 3D Complex Website. https://shmoo.weizmann.ac.il/elevy/3dcomplexV6/Home.cgi.

[78] Sievers F., Wilm A., Dineen D., Gibson T.J., Karplus K., Li W., Lopez R., McWilliam H., Remmert M., Söding J. (2011). Fast, scalable generation of high-quality protein multiple sequence alignments using Clustal Omega. Mol. Syst. Biol..

[79] Chan D.C., Fass D., Berger J.M., Kim P.S. (1997). Core Structure of gp41 from the HIV Envelope Glycoprotein. Cell.

[80] Borhani D.W., Rogers D.P., Engler J.A., Brouillette C.G. (1997). Crystal structure of truncated human apolipoprotein A-I suggests a lipid-bound conformation. Proc. Natl. Acad. Sci. USA.

[81] Knowlton J.R., Johnston S.C., Whitby F.G., Realini C., Zhang Z., Rechsteiner M., Hill C.P. (1997). Structure of the proteasome activator REG_α_(PA28_α_). Nature.

[82] Hoffman D., Davies C., Gerchman S., Kycia J., Porter S., White S., Ramakrishnan V. (1994). Crystal structure of prokaryotic ribosomal protein L9: A bi-lobed RNA-binding protein. EMBO J..

[83] Jasanoff A., Wagner G., Wiley D.C. (1998). Structure of a trimeric domain of the MHC class II-associated chaperonin and targeting protein Ii. EMBO J..

[84] Tan K., Liu J.H., Wang J.H., Shen S., Lu M. (1997). Atomic structure of a thermostable subdomain of HIV-1 gp41. Proc. Natl. Acad. Sci. USA.

[85] Bhaumik P., Koski M.K., Bergmann U., Wierenga R.K. (2004). Structure determination and refinement at 2.44 AA resolution of argininosuccinate lyase from *Escherichia coli*. Acta Crystallogr. Sect. D Biol. Crystallogr..

[86] Sideraki V., Wilson D.K., Kurz L.C., Quiocho F.A., Rudolph F.B. (1996). Site-Directed Mutagenesis of Histidine 238 in Mouse Adenosine Deaminase: Substitution of Histidine 238 Does Not Impede Hydroxylate Formation. Biochemistry.

[87] Förster A., Masters E.I., Whitby F.G., Robinson H., Hill C.P. (2005). The 1.9 AA Structure of a Proteasome-11S Activator Complex and Implications for Proteasome-PAN/PA700 Interactions. Mol. Cell.

[88] Feng L., Wanninayake U., Strom S., Geiger J., Walker K.D. (2011). Mechanistic, Mutational, and Structural Evaluation of a Taxus Phenylalanine Aminomutase. Biochemistry.

[89] García-Nafría J., Timm J., Harrison C., Turkenburg J.P., Wilson K.S. (2013). Tying down the arm inBacillus dUTPase: Structure and mechanism. Acta Crystallogr. Sect. D Biol. Crystallogr..

[90] Austin S.K., Dowd K.A., Shrestha B., Nelson C.A., Edeling M.A., Johnson S., Pierson T.C., Diamond M.S., Fremont D.H. (2012). Structural Basis of Differential Neutralization of DENV-1 Genotypes by an Antibody that Recognizes a Cryptic Epitope. PLoS Pathog..

[91] Stellmacher L., Sandalova T., Leptihn S., Schneider G., Sprenger G.A., Samland A.K. (2015). Acid-Base Catalyst Discriminates between a Fructose 6-Phosphate Aldolase and a Transaldolase. ChemCatChem.

[92] Söderholm A., Guo X., Newton M.S., Evans G.B., Näsvall J., Patrick W.M., Selmer M. (2015). Two-step Ligand Binding in a (βα)8 Barrel Enzyme. J. Biol. Chem..

[93] Zhao H., Fernandez E., Dowd K.A., Speer S.D., Platt D.J., Gorman M.J., Govero J., Nelson C.A., Pierson T.C., Diamond M.S. (2016). Structural Basis of Zika Virus-Specific Antibody Protection. Cell.

[94] Banach M. (2022). Symmetrization in the Calculation Pipeline of Gauss Function-Based Modeling of Hydrophobicity in Protein Structures. Symmetry.

[95] Hubbard S., Thornton J. (1993). NACCESS, Computer Program.

[96] Ribeiro J., Ríos-Vera C., Melo F., Schüller A. (2019). Calculation of accurate interatomic contact surface areas for the quantitative analysis of non-bonded molecular interactions. Bioinformatics.

[97] dr-sasa Repository. https://github.com/nioroso-x3/dr_sasa_n.

[98] (2023). The PyMOL Molecular Graphics System.

[99] Hunter J.D. (2007). Matplotlib: A 2D Graphics Environment. Comput. Sci. Eng..

[100] Deng A.W., Gwo C.Y. (2020). A Stable Algorithm computing high-order 3D Zernike Moments and Shape Reconstructions. Proceedings of the 4th International Conference on Digital Signal Processing.

[101] van Kempen M., Kim S.S., Tumescheit C., Mirdita M., Lee J., Gilchrist C.L.M., Söding J., Steinegger M. (2023). Fast and accurate protein structure search with Foldseek. Nat. Biotechnol..

[102] Foldseek Website. https://search.foldseek.com.

[103] Koehl P., Orland H. (2023). A Physicist’s View on Partial 3D Shape Matching. Algorithms.

