# Supplementary material for: Structural Outlier Detection and Zernike–Canterakis Moments for Molecular Surface Meshes—Fast Implementation in Python
Source: Molecules. 2023 Dec 21;29(1):52. doi: 10.3390/molecules29010052 (PMC10779519; doi:10.3390/molecules29010052)
Supplement: Supplementary file 1 [file molecules-29-00052-s001.zip › supplement1.pdf]

Supplementary Materials: Structural Outlier Detection and Zernike–Canterakis Moments for Molecular Surface Meshes—Fast Implementation in Python

Mateusz Banach

S1. The BioZernike Assembly Subset

Table S1. PDB codes and biological assembly numbers of the 450 base structures extracted from the BioZernike "assemblies" validation suite. The original suite has 500 models.

1ais_1	1am9_1	1b0n_5	1bih_1	1cfr_1	1dp7_1	1esc_1	1ex4_5	1f2i_2	1foe_3
1fpo_4	1fwy_2	1g8p_1	1gqe_1	1ik9_1	1j1e_2	1joc_1	1kb6_1	1kb9_1	1mc0_1
1mg7_1	1n7v_1	1nkz_2	1no4_3	1orq_1	1ov3_2	1p7h_2	1ppr_1	1q90_1	1qbz_2
1qg3_1	1qo0_1	1quu_1	1r4a_1	1r71_2	1rfx_3	1sf8_9	1v8d_3	1v9d_1	1vf6_3
1w26_1	1wio_1	1wwl_1	1xfx_1	1yo8_1	1yvl_2	1z56_1	1zme_1	2ayu_1	2bap_2
2c2v_1	2c9o_1	2d4c_1	2dfs_1	2dgj_2	2dyb_2	2e31_1	2e7s_2	2exd_1	2f1m_1
2fhd_2	2fhd_4	2gww_2	2h88_3	2hsn_1	2i2r_2	2icw_2	2ju4_1	2jwh_1	2kr0_1
2kyh_1	2lp1_1	2m0q_1	2m1s_1	2m6i_1	2m74_1	2mar_1	2mzw_1	2n2a_1	2n73_1
2nae_1	2noq_1	2nsa_2	2nwa_3	2nzi_1	2oar_1	2odm_1	2okq_2	2p0k_2	2p22_6
2pr1_1	2q9q_1	2qg7_1	2qj6_2	2qx5_1	2r32_1	2r9r_7	2rax_2	2vz9_1	2w4u_1
2we8_1	2xig_1	2xnx_1	2xzz_1	2y25_2	2ygg_1	2ylm_1	2ynq_3	2zdi_1	2zi0_2
2zqm_2	2zxx_2	3a69_1	3ax1_1	3b43_1	3bes_2	3bes_3	3bk3_3	3bn3_1	3c0n_1
3c7j_1	3cqx_1	3cu7_3	3cw2_4	3cyg_1	3d55_4	3ddv_3	3dkw_1	3eq2_1	3esi_1
3ezj_3	3frw_1	3gge_2	3ghg_2	3gq8_2	3hyi_1	3hym_7	3i4r_1	3ibp_2	3ivf_1
3j0q_1	3j1r_1	3j8b_1	3jsz_1	3k66_1	3kdp_2	3kfd_3	3kxr_1	3l1e_1	3ljb_2
3lmn_3	3lou_3	3lue_1	3lz8_1	3mky_2	3mpx_1	3mq7_2	3msv_3	3muu_1	3mw6_3
3mzk_1	3n56_2	3n57_1	3nb0_1	3nb2_2	3nrg_3	3nrk_1	3o0z_1	3o4z_4	3oa7_1
3oky_1	3ov0_1	3pod_3	3prb_1	3psf_1	3pxi_1	3py9_1	3q4f_1	3qb2_2	3qcw_2
3qil_2	3qs7_1	3r2p_1	3r6n_1	3r9v_3	3rbz_1	3rip_2	3rv0_1	3sf4_2	3sja_2
3syy_1	3tkl_1	3tos_7	3tul_2	3tul_5	3u1w_1	3u24_1	3uau_2	3ubg_3	3uej_1
3ukm_2	3usw_1	3vp5_1	3vx8_2	3wbk_1	3wcy_1	3win_1	3wpa_1	3zbj_1	3ziw_2
3zms_1	4a2q_1	4aj5_1	4aqb_1	4b7l_2	4bjm_1	4bm5_1	4bpx_3	4c00_1	4c16_2
4cg4_3	4ci6_1	4crm_1	4dl0_1	4e0g_1	4eba_2	4eoz_2	4ezp_4	4f2m_1	4f7g_1
4fb3_1	4fcy_1	4fuv_1	4fzx_1	4g7n_2	4gc5_2	4gyv_1	4h63_1	4hyg_1	4i2z_1
4i8o_3	4igg_1	4ild_1	4j80_1	4jgw_2	4jio_1	4jle_2	4jvy_1	4jx0_3	4jza_2
4k3b_1	4k3c_1	4ke2_1	4kik_1	4kjm_2	4l6v_1	4lct_2	4lgd_6	4mbe_2	4mrs_1
4mte_1	4mu6_1	4mzu_2	4oci_1	4ofy_1	4om3_1	4or2_1	4ot9_2	4p6q_1	4pa0_1
4plm_1	4pnh_1	4q0w_1	4q66_2	4qcc_1	4ql6_1	4qlb_2	4qnd_2	4r7q_1	4rgu_1
4rsj_2	4s3o_2	4tko_1	4u0q_2	4u0s_1	4uf5_1	4w8c_1	4wid_1	4wij_1	4x01_1
4x0j_2	4x4w_1	4x8w_3	4xk8_2	4xl1_2	4xl1_3	4xng_1	4xz7_1	4y1l_1	4y66_1
4y99_1	4ycz_1	4yg8_1	4yh7_1	4yn0_1	4ypi_4	4yzf_1	4z6y_2	4z7f_3	4zlh_1
4zmm_1	4zu9_1	4zuz_2	5a20_1	5aef_1	5afr_1	5b0o_4	5b2g_2	5bo1_1	5c0r_1
5c22_3	5cbn_1	5csa_1	5csm_1	5cws_1	5dl2_2	5dqq_1	5e7g_1	5edv_3	5eyb_1
5f5p_1	5fcm_2	5fhy_2	5gzt_1	5h0s_1	5h1a_3	5h77_5	5h7a_1	5hb0_2	5hox_4
5hxy_5	5hzv_1	5i6f_1	5i6r_1	5i8g_1	5ica_1	5iit_1	5iw9_1	5iws_1	5j67_2
5j69_1	5j9u_2	5jh0_1	5jhf_1	5jne_1	5jtw_1	5jxl_1	5kcn_1	5kes_1	5ktf_1
5l0w_1	5l56_1	5lcb_1	5ljo_1	5lob_1	5lsj_1	5lsk_1	5ltw_1	5luq_2	5ly9_1
5m4y_3	5m88_1	5mg8_2	5mio_3	5mnt_3	5mzv_1	5n2b_1	5n7w_1	5nca_1	5nen_1
5nf8_1	5nl6_1	5ntu_1	5o32_1	5ods_4	5ojy_1	5ool_1	5oqq_2	5ot4_2	5owv_1
5sv1_1	5svk_2	5tsx_2	5tzs_1	5ucg_2	5uk5_1	5un6_2	5v3j_1	5vaz_3	5vfz_1
5vgz_1	5vhs_1	5vi4_2	5vj4_1	5vvr_1	5w5c_1	5we1_1	5wgb_1	5wql_2	5wtl_1
5xis_1	5xll_1	5xpy_1	5xyn_1	5yan_1	5yti_1	5z51_1	5zcs_1	5zyu_2	6ao5_1
6b0n_1	6b8f_1	6bfi_1	6bhf_1	6bn1_2	6bog_1	6bq1_1	6br9_1	6buh_2	6byl_2
6c9u_1	6ce7_1	6ces_1	6cfx_1	6cl6_2	6cxo_3	6d12_1	6duz_1	6ecm_1	6ek4_4
6emk_1	6epg_1	6f8l_1	6fes_2	6fj3_1	6fln_2	6gff_1	6gym_1	6hts_1	6ijz_1

The structures can be downloaded from https://github.com/rcsb/biozernike-validation.

S2. The Example Protein Database

The superposition of pairs of similar proteins was carried out with the CE algorithm implemented in PyMOL v. 2.5.0. The results are given in the text in the "RMSD / number of aligned residues" notation. The slash means "over". Due to the nature of the CE algorithm, the number of those residues may be smaller (possibly a lot) than the number of residues comprising the structures for which the RMSD was requested.

The multiple sequence alignment was performed with Clustal Omega v. 1.2.4.

S2.1. HIV-1 GP41 Subdomain (1AIK, 1SZT)

The HIV-1 GP41 subdomain (PDB codes: 1AIK and 1SZT, Figure S1) is a bundle of 6 helices that twist around the central axis of symmetry. Two helices comprise the asymmetric unit. They are assigned to one chain in 1SZT and to two chains in 1AIK (1AIK is a SCOP genetic domain). The G36–W41 loop between the helices is missing from 1AIK.

The assemblies have highly similar structures, but they do not superpose very well (Figure S2). To align with the sequence of 1SZT:A (86.8% identity), chain N in 1AIK must be placed before chain C (i.e., like it was placed in the PDB file by the depositors). An RMSD of 7.76 Å / 80 aa means that methods based on the direct atomic coordinate comparison may have trouble detecting the high structural similarity of these molecules. Conversely, the RMSD between the asymmetric units is only 2.52 Å / 56 aa.

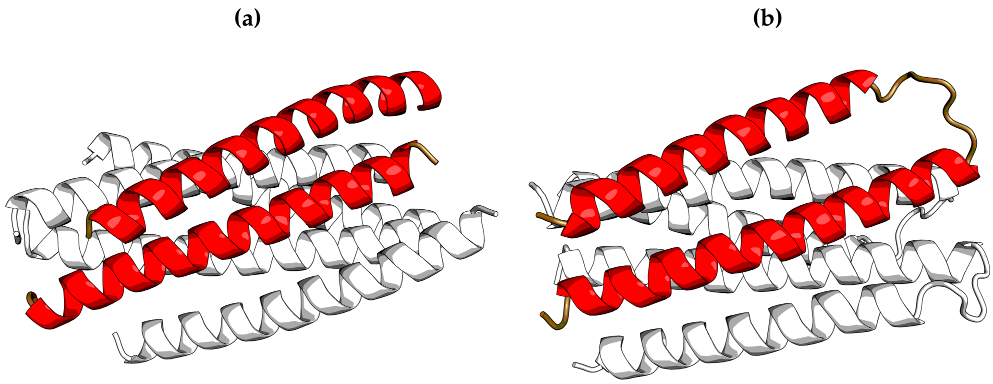

Figure S1. The structures of **(a)** 1AIK and **(b)** 1SZT. Colors distinguish 1AIK:N+C and 1SZT:A.

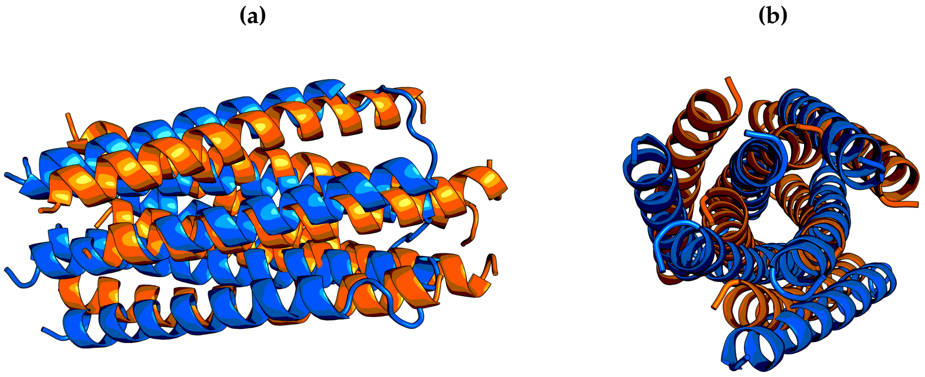

Figure S2. The superposition of 1AIK and 1SZT: **(a)** side view, **(b)** top view.

S2.2. Apolipoprotein A-I (1AV1)

The apolipoprotein A-I (PDB code: 1AV1, Figure S3) is a tetrameric coiled coil that significantly differs from the other example proteins. Each of its four chains assumes a horseshoe conformation with a vast void in the middle. The lack of structural similarity versus the other examples should be corroborated by the shape retrieval methods.

(a) **(b)**

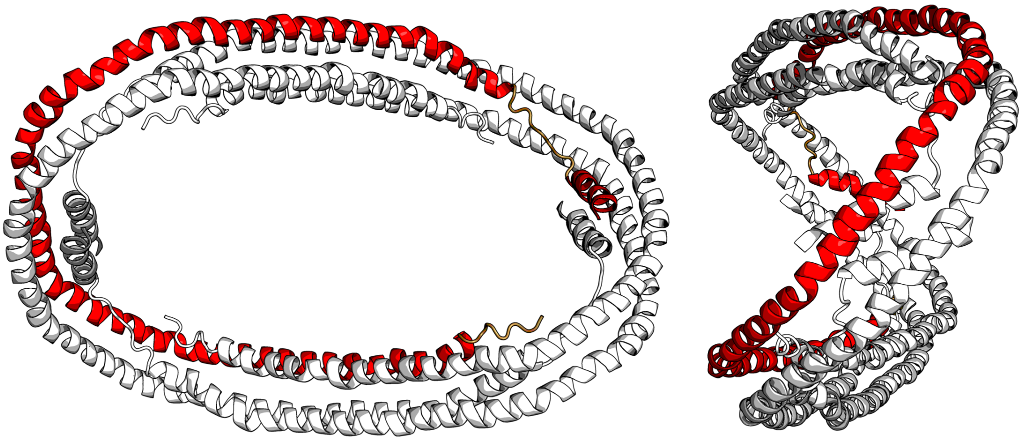

Figure S3. The structure of 1AV1: **(a)** top view, **(b)** side view. Colors distinguish 1AV1:A.

S2.3. Proteasome PA28 Activator (1AVO), Proteasome PA26+20S Complex (1YAR)

The proteasome-11S activator is a heptameric “cap” attached to each end of the “core” proteasome 20S chamber. The chamber is formed by four stacked rings of α and β subunits. The structure of a lone PA28 activator has PDB code 1AVO (Figure S4a). The structure of two PA26 activators in complex with 20S has PDB code 1YAR (Figure S4b).

The seven chains in 1YAR:O–U (i.e., in one of the PA26s) correspond to the seven pairs of chains in 1AVO (1AVO is a SCOP genetic domain). 1AVO and 1YAR:O–U have only 17% of sequence identity, but their structural similarity is very high, as corroborated by RMSD of 2.91 Å / 192 aa. This is, however, the outcome of the superposition of their asymmetric units: 1AVO:A+B and 1YAR:O. It is enough to produce a fitting structural overlap of the whole activators (Figure S5), although some local discrepancies are noticeable.

The T224–S231 C-terminal fragments that connect 1YAR:O–U with the rest of 1YAR are stretching away from the main body of the activator (Figure S5a) and its inner loops at G126–M142 are absent in 1AVO (Figure S5b). The entire 1YAR complex should be seen by the structural similarity metrics as completely different from the other example proteins. It is a large molecule with a length of almost 300 Å.

(a) **(b)**

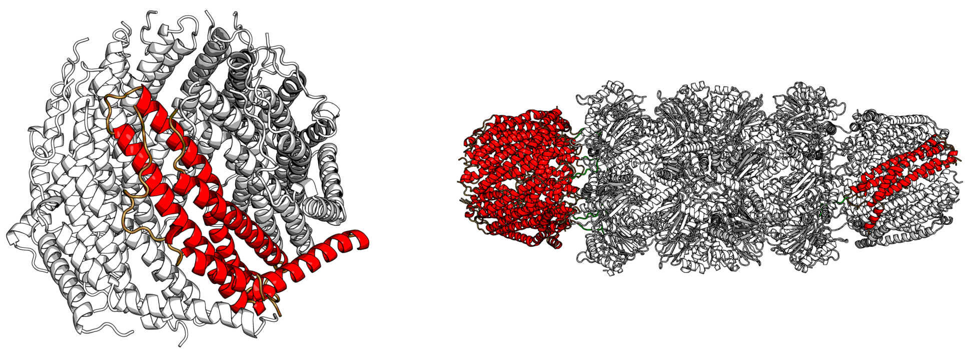

Figure S4. The structures of **(a)** 1AVO and **(b)** 1YAR. Colors distinguish 1AVO:A+B, 1YAR:O and the other PA26 that lies on the opposite end of 1YAR in respect to 1YAR:O–U. The T224–S231 fragments are marked on **(b)** in green.

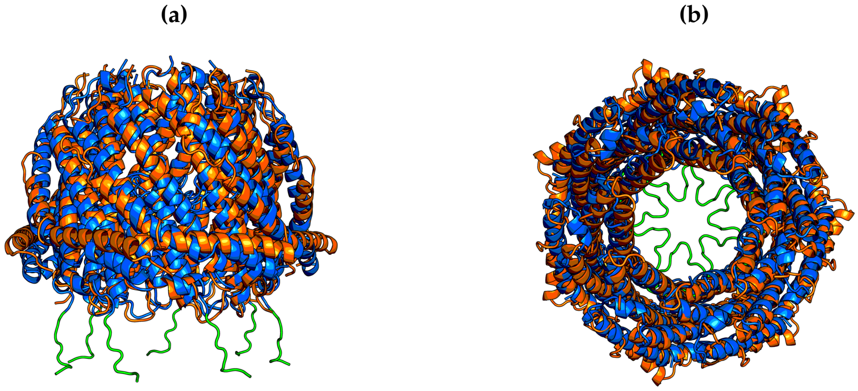

Figure S5. The superposition of 1AVO and 1YAR:O–U: **(a)** side view, **(b)** top view. Green color marks the T224–S231 and G126–M142 fragments in 1YAR:O–U.

S2.4. Ribosomal Protein L9 (1DIV)

The ribosomal Protein L9 (PDB code: 1DIV, Figure S6) is the only structure in this set with multiple SCOP domains per chain. These domains, M1–Q55 and R56–K149, are connected by the central helix. The dimeric assembly, recreated via the symmetry operators, has a Y-shaped conformation that does not resemble any other example protein.

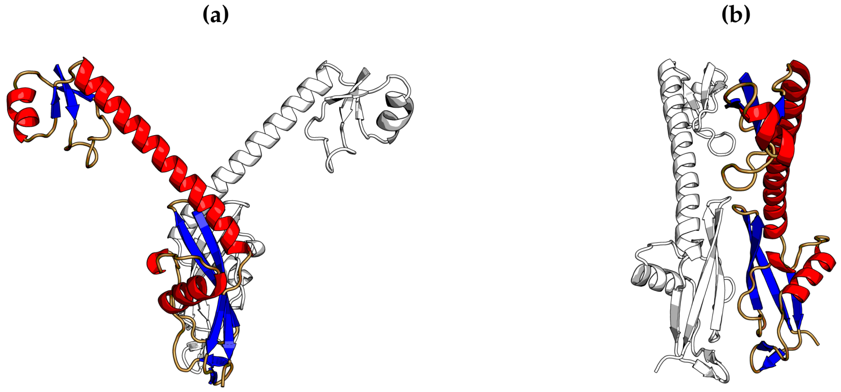

Figure S6. The structure of 1DIV: **(a)** front view, **(b)** side view. Colors distinguish 1DIV:A. The M1–Q55 domains are near the top of the figure. The R56–K149 domains are interacting.

S2.5. HLA-DR Invariant Chain (1IIE)

The HLA-DR invariant chain (PDB code: 1IIE, Figure S7) is a homotrimer obtained via solution NMR. Its chains are loose, but together form a dense ball-like assembly. The disordered C-termini (Q184–K192) protrude into the solvent, causing the apparent size of the molecule to increase. They should be found by an outlier detection subroutine.

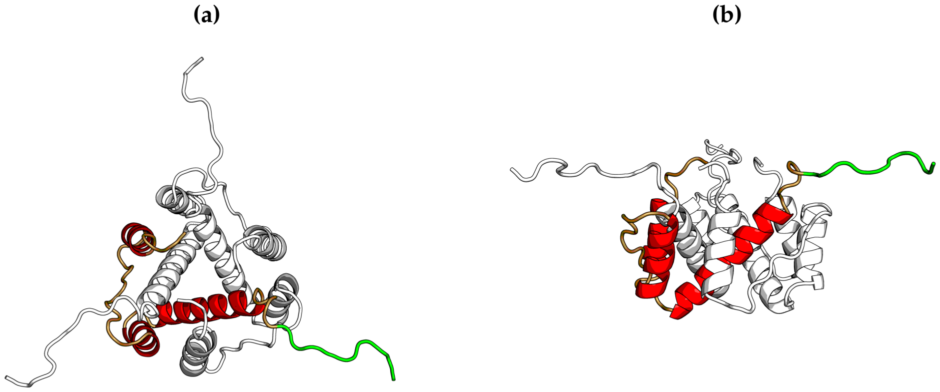

Figure S7. The structure of conformer 1/20 of 1IIE: **(a)** top view, **(b)** side view. Colors distinguish 1IIE:A. The Q184–K192 fragment is marked in green.

S2.6. Argininosuccinate Lyase (1TJ7), Phenylalanine Aminomutase (3NZ4)

The argininosuccinate lyase (PDB code: 1TJ7, Figure S8a) and the phenylalanine aminomutase (PDB code: 3NZ4, Figure S8b) belong to the same SCOP superfamily and are the only members of the same 3D Complex QS Family cluster. Their relatively large monomers have a very low sequence identity (12.3%) and a moderate structural similarity (Figure S9a). Over 50% more residues in 3NZ4 contributes to RMSD of 7.16 Å / 168 aa. The dimers have smaller RMSD over the same number of residues (6.20 Å, Figure S9b), but they also possess different complex symmetries.

(a) **(b)**

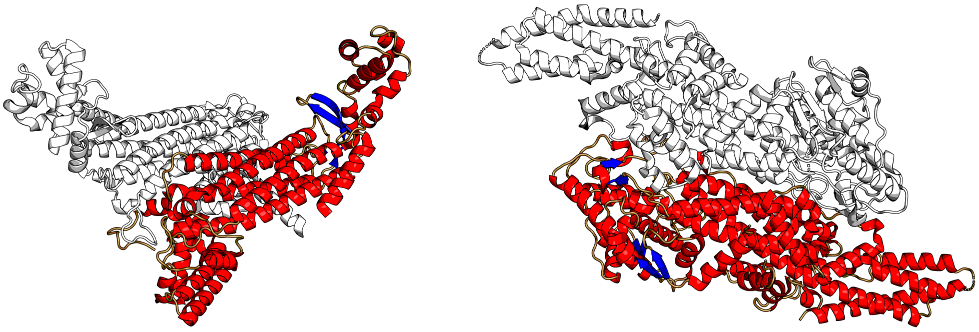

Figure S8. The structures of **(a)** 1TJ7 and **(b)** 3NZ4. Colors distinguish 1TJ7:A and 3NZ4:A.

(a) **(b)**

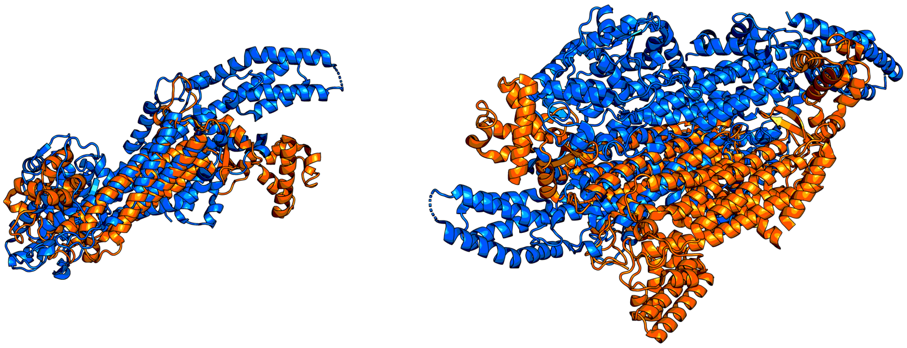

Figure S9. The superpositions of **(a)** 1TJ7:A and 3NZ4:A, and **(b)** 1TJ7:A+B and 3NZ4:A+B.

S2.7. Fructose-6-phosphate Aldolase (4RXF), HisA Enzyme (5AHE), Adenosine Deaminase (1UIP)

The asymmetric unit of fructose-6-phosphate aldolase (PDB code: 4RXF, Figure S10a) and the monomeric HisA enzyme (PDB code: 5AHE, Figure S10b) have similar shapes and sizes (RMSD 4.01 Å / 168 aa, Figure S11a), but the extended C-terminus in 4RXF at S196–I220 may confuse the structural similarity metrics. This "tail" should be removed via an outlier detection subroutine. The adenosine deaminase (PDB code: 1UIP, Figure S10c) is the larger cousin of 4RXF and 5AHE from SCOP c.1 TIM beta/alpha-barrel common fold, exhibiting versus them an RMSD of 5.39 Å / 152 aa and 5.89 Å / 176 aa (Figure S11b,c).

(a) **(b)** **(c)**

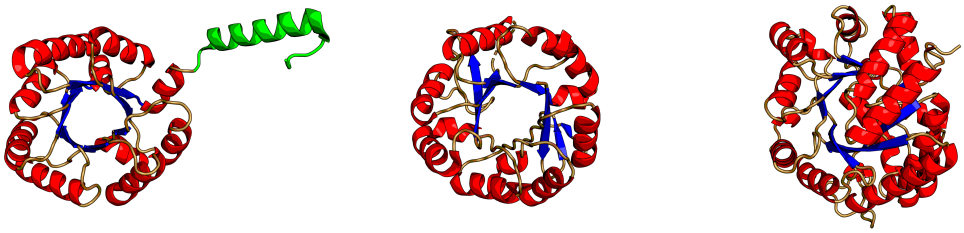

Figure S10. The structures of **(a)** 4RXF (S196–I220 fragment marked in green), **(b)** 5AHE and **(c)** 1UIP.

The assembly of 4RXF has a D5 symmetry. It was not included in this experiment.

(a) **(b)** **(c)**

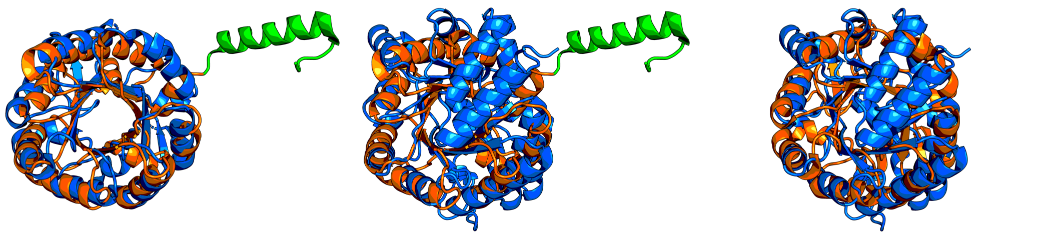

Figure S11. The superpositions of **(a)** 4RXF and 5AHE (12.8%), **(b)** 4RXF and 1UIP (13.3%), and **(c)** 5AHE and 1UIP (11.5%). The values in the parentheses are the percentages of sequence identity.

S2.8. dUTPase YncF (4B0H)

The dUTPase YncF (PDB code: 4B0H, Figure S12) is a homotrimer with long, disordered C-terminal fragments at P120–K144. These fragments protrude from the monomers, but are flush with the body of the complex. 4B0H:A is also truncated at G131, which pushes it away from the other two chains in terms of structural similarity metrics. This is another example of a protein that may benefit from an outlier detection subroutine.

(a) **(b)**

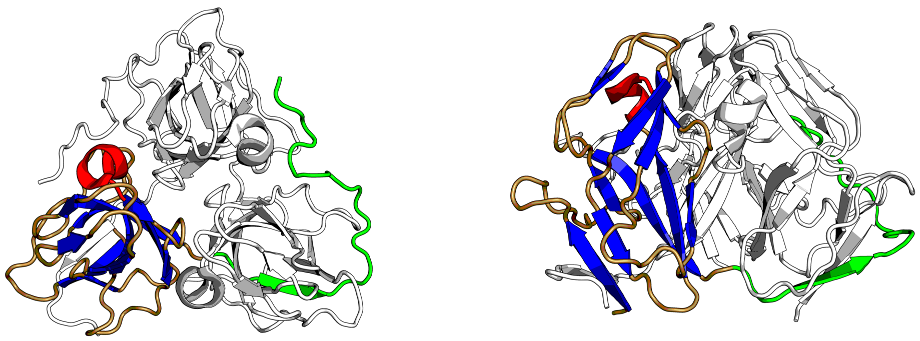

Figure S12. The structure of 4B0H: **(a)** top view, **(b)** side view. Colors distinguish 4B0H:B. The P120–K144 fragment is marked in green.

S2.9. DENV-1 Antibody E111 (4FFY) and ZIKV Antibody ZV-48 (5KVE)

The Dengue virus antibody E111 (PDB code: 4FFY, Figure S13a) and the Zika virus antibody ZV-48 (PDB code: 5KVE, Figure S13b) are house mouse proteins in complex with viral envelope chains. 4FFY:H+L and 5KVE:L are the antibodies. Their sequence identity is 34.3% and their RMSD is 3.27 Å / 216 aa (Figure S14a). The same RMSD is returned by the CE algorithm for the whole complexes. The viral proteins are also highly similar (Figure S14b). They exhibit sequence identity of 44.5% and RMSD of only 1.51 Å / 96 aa.

(a) **(b)**

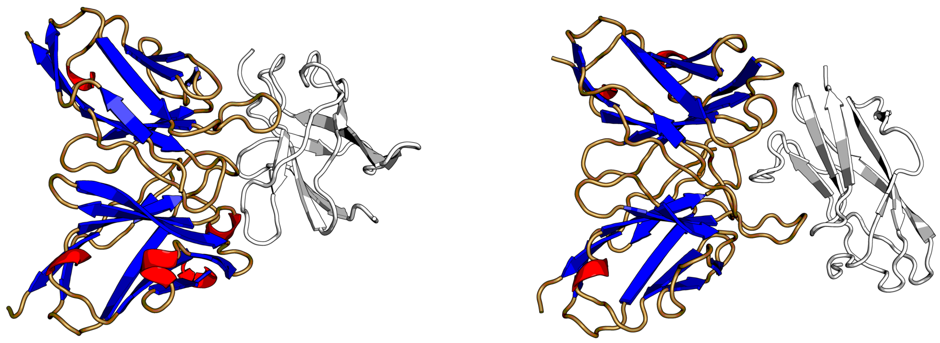

Figure S13. The structures of **(a)** 4FFY and **(b)** 5KVE. Colors distinguish 4FFY:H+L and 5KVE:L.

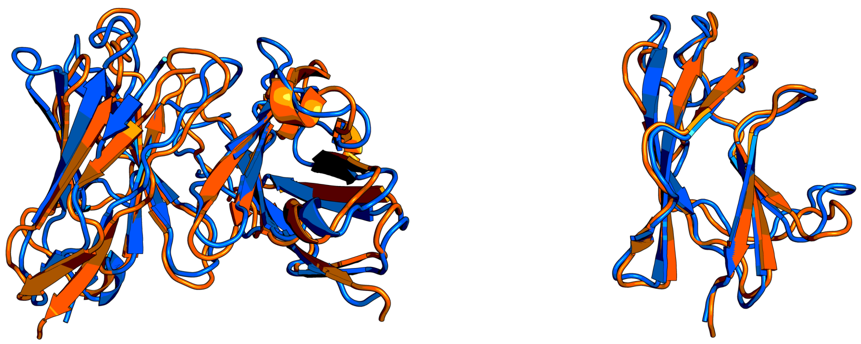

Figure S14. The superpositions of **(a)** 4FFY:H+L and 5KVE:L, and **(b)** 4FFY:A and 5KVE:E.

S3. The Detection of Outlier Residues

This is the PCA-based algorithm for the detection of structural outliers in proteins. It is used by M. Banach as an optional subroutine in the Ellipsoid Profile algorithm (EP). Its alignment step and W. Kabsch's superposition method are based on the same principle.

S3.1. The Input Parameters

Let P be a real array with size $n \times d$. Each row contains the coordinates of one of n points (e.g., atom or effective atoms) in d-dimensional space ($d \geq 1$).

Let $0 < p < 1$ be the confidence level chosen by the user. Its recommended default value for this algorithm is 0.9. In practice (i.e., when implemented on a computer), $p \leq 0$ should cause all points to be returned as outliers and $p \geq 1$ should cause all points to be returned as "guides" (i.e., not outliers). The value of p is ignored if $r \leq 0$ (see below).

Let $r \geq 0$ be the number of detection rounds (iterations). Its recommended default value is 3. If $r \leq 0$, the algorithm is bypassed. In practice, this should cause all points to be returned as guides since no outlier detection was carried out.

S3.2. The Axis Alignment

Shift P to the origin: calculate its centroid, $\overline{P}$ (i.e., the average position of all points) and subtract it from each point component-wise. Q is the array with the centered points:

$$Q = P - \overline{P}. \tag{1}$$

Rotate Q in alignment with the principal axes of the coordinate system. The optimal rotation matrix, V, is obtained via the singular value decomposition (SVD) of Q:

$$USV^T = \mathrm{SVD}(Q). \tag{2}$$

Array U ($n \times n$) holds the eigenvectors of QQ^T, array S ($n \times d$) holds the eigenvalues of Q and array V ($d \times d$) holds the eigenvectors of Q^TQ. U and V are orthonormal.

A compact type of SVD is preferred when $d \ll n$ or $n \ll d$. It is faster and avoids the $O(\max(n, d)^2)$ memory requirement of the full-size U and V arrays. If the compact SVD is unavailable and $d \ll n$, Q in Equation (2) can be replaced with Q^TQ. Its SVD produces the same rotation in V, but does it faster as long as d is low.

The multiplication of Q by V maximizes the variance of the points in each dimension and diagonalizes the cross-covariance matrix of their coordinates. The geometric interpretation of this procedure is the fitting of a d-dimensional ellipsoid to the data through minimization of the point distance to its radii. The axis-aligned points are in array R:

$$R = QV. \tag{3}$$

If the coordinates of the points were their quantitative features, this would transform them to a new coordinate system of new, uncorrelated features (i.e., as in actual PCA).

Depending on P, V may be a rotation-reflection matrix, causing R to become a mirror image of Q. It is a side effect of the SVD algorithm. To fix it, check the determinant of V (reflection matrices have det = -1) and flip the sign of one of the eigenvectors. A common choice is the last eigenvector (i.e., the last column of V), with the lowest eigenvalue:

$$R = QV \begin{pmatrix} 1 & 0 & \cdots & 0 \\ 0 & 1 & \cdots & 0 \\ \vdots & \vdots & \ddots & \vdots \\ 0 & 0 & \cdots & \mathrm{sign}(\det(V)) \end{pmatrix}. \tag{4}$$

Equation (4) appends another reflection to Equation (3) when $\det(V) < 0$. It corrects the transformation owing to the fact that an even number of reflections by the hyperplanes containing the origin results in a rotation. The new R is no longer a mirror image of Q.

S3.3. The Confidence Ellipsoid

Determine the lengths of the radii (i.e., half-axes) of an axis-aligned confidence ellipsoid of the points from array R. Without loss of generality, lets assume that $d = 3$.

Point $[x, y, z]$ lies on the surface of the confidence ellipsoid when:

$$\left(\frac{x}{\sigma_{\mathrm{x}}}\right)^2 + \left(\frac{y}{\sigma_{\mathrm{y}}}\right)^2 + \left(\frac{z}{\sigma_{\mathrm{z}}}\right)^2 = s = \chi^2_{(p,d=3)}. \tag{5}$$

Vectors $[x, 0, 0]$, $[0, y, 0]$ and $[0, 0, z]$ define the three radii of this ellipsoid. Passing them to Equation (5) yields their lengths: $\sigma_{\mathrm{x}}\sqrt{s}$, $\sigma_{\mathrm{y}}\sqrt{s}$ and $\sigma_{\mathrm{z}}\sqrt{s}$, where $\sigma_{\mathrm{x}}, \sigma_{\mathrm{y}}, \sigma_{\mathrm{z}}$ are the standard deviations of all points in each dimension. The value of the scale factor s is taken from the χ^2 distribution with d degrees of freedom. For $d = 3$ and $p = 0.9$, $\sqrt{s} \approx 2.500278$.

Point $[x, y, z]$ is within the range of the confidence ellipsoid when:

$$\left(\frac{x}{\sigma_{\mathrm{x}}\sqrt{s}}\right)^2 + \left(\frac{y}{\sigma_{\mathrm{y}}\sqrt{s}}\right)^2 + \left(\frac{z}{\sigma_{\mathrm{z}}\sqrt{s}}\right)^2 \leq 1. \tag{6}$$

All points from R for which Equation (6) is true are the guides. The other points, that lie outside the confidence ellipsoid, are the outliers.

S3.4. The Detection Rounds

There is no universal value of p that is robust against all distributions of points. For this reason, the above detection procedure is ran at most r times in succession. The current guide subset, selected with Equation (6), becomes the input (i.e., P) for the next round. The loop terminates after rth round, or when there are no new outliers, or when there are no more guides. This means that the convergence of the algorithm is guaranteed even when $r \to \infty$, but it may become then overzealous, carving deep into the input data set.

S3.5. The Implementation

The following Python code finds the outliers in a cloud of n points in d dimensions. It requires NumPy and SciPy. This is an implementation of a single detection round.

Listing 1. Detection of outliers in a cloud of points.

```
# Supply array P with n points (d columns)
P = numpy.atleast_2d(numpy.asarray(P, numpy.float64))
# Choose the confidence level (0.0 < p < 1.0)
p = 0.9
# ----------------------------------------------------------------------
# Translate P to the origin
Q = P - numpy.average(P, 0)
# Calculate the principal components of Q (compact SVD)
U, S, Vt = numpy.linalg.svd(Q, False)
# Check for reflection and fix the rotation matrix
```

```
Vt[-1] *= numpy.sign(numpy.linalg.det(Vt))
# Rotate Q in alignment with the principal axes
R = numpy.dot(Q, Vt.T)
# ---------------------------------------------------------------------------
# Get the scale factor from the chi square distribution
s = numpy.sqrt(scipy.stats.chi2.ppf(p, P.shape[1]))
# Calculate the radii of the confidence ellipsoid
r = numpy.std(R, 0) * s
# Get the value of the ellipsoid equation for all points
E = numpy.sum(numpy.power(R / r, 2), 1)
# Extract the outlier point subset (outside the ellipsoid)
O = numpy.compress(E > 1, R, 0)
# Extract the guide point subset (not outside the ellipsoid)
G = numpy.compress(E <= 1, R, 0)
```

S4. The Decimation of a Surface Mesh

The following Python code decimates an unstructured surface mesh stored in vertex and facet arrays. It requires NumPy and PyVista (and VTK as PyVista's dependency).

Listing 2. Decimation of a surface mesh.

```
# Supply array V with n vertices of the mesh (3 columns)
V = numpy.atleast_2d(numpy.asarray(V, numpy.float64))
# Supply array F with m facets of the mesh (3 columns)
F = numpy.atleast_2d(numpy.asarray(F, numpy.int64))
# Choose the decimation factor (0.0 <= d < 1.0)
d = 0.5
# Choose the decimation algorithm:
# False - uniform triangles / vtkQuadricDecimation
# True - adaptive triangles / vtkDecimatePro
pro = False
# ---------------------------------------------------------------------------
# Generate count array with 3 as the number of vertices in each facet
C = numpy.full((F.shape[0], 1), 3)
# Prepend C to F (column-wise) and flatten them into a list
CF = numpy.ravel(numpy.hstack((C, F)))
# Create the mesh PolyData object (the third argument is optional)
M = pyvista.PolyData(V, CF, F.shape[0]))
# Ensure that the mesh is free of artifacts (optional)
M = M.clean().triangulate()
# Apply the chosen mesh decimation algorithm
if pro:
  M = M.decimate_pro(d, preserve_topology=True)
else:
  M = M.decimate(d)
# ---------------------------------------------------------------------------
# Display the decimated mesh and show its volume (optional)
M.plot(show_edges=True, text='Volume: {0:0.1f}'.format(M.volume))
# Retrieve the vertex and facet arrays of the decimated mesh
V = numpy.asarray(M.points, numpy.float64)
F = numpy.reshape(M.faces, (M.n_faces, 4))[:, 1:]
```

S5. The Pozo–Koehl (PK) Algorithm

This is the exact Pozo–Koehl algorithm for the calculation of Zernike–Canterakis (ZC) moments from volume-like geometric moments of an unstructured surface mesh. It was introduced by J. Pozo, M.-C. Villa-Uriol and A. Frangi, and later optimized by P. Koehl.

S5.1. The Input Parameters

Let $M = \{F_1 \ldots F_m\}$ be an unstructured surface mesh made up from m triangular facets. Each facet F_f is defined by its vertices: $F_f = (u_f, v_f, w_f)$, where $u_f = [u_{f\mathrm{x}}, u_{f\mathrm{y}}, u_{f\mathrm{z}}]$, $v_f = [v_{f\mathrm{x}}, v_{f\mathrm{y}}, v_{f\mathrm{z}}]$, $w_f = [w_{f\mathrm{x}}, w_{f\mathrm{y}}, w_{f\mathrm{z}}]$ and $1 \leq f \leq m$. The vertices u_f, v_f, w_f must be ordered counter-clockwise when looking at F_f from the exterior of the mesh. In practice, u_f, v_f, w_f are indices of rows (i.e., integers) in a common vertex array of the mesh.

Let $N \geq 0$ be the moment order chosen by the user. $N = 20$ is the popular choice in structural bioinformatics. Moment orders above 35 lead to numerical instabilities.

S5.2. The Unit Ball Fitting

Shift M to the origin: calculate its centroid (i.e., the average position of all vertices) and subtract it from each vertex component-wise.

Scale down M by dividing each vertex (component-wise) by the unit ball scale factor. This factor, calculated automatically or supplied by the user, must be large enough so that none of the scaled vertices is outside the unit sphere. This means that its lower bound is the longest vertex distance to the origin (after the translation). There is no upper bound—the mesh can be scaled further down, away from the unit sphere.

S5.3. The Geometric Moments

Allocate real array G with size $(N+1) \times (N+1) \times (N+1)$. The geometric moments of M are stored in G at indices i, j, k, where $0 \leq i, j, k \leq N$, subject to $i + j + k \leq N$. The number of those moments is $(N+1)(N+2)(N+3)/6$, for example, 1771 at $N = 20$.

The value of $G_{i,j,k}(M)$ is given by the following formula:

$$G_{i,j,k}(M) = 6\frac{i!\,j!\,k!}{(i+j+k+3)!}\sum_{f=1}^{m}\left[\mathrm{vol}\left(F_f\right)S_{i,j,k}\left(F_f\right)\right], \tag{7}$$

where $\mathrm{vol}(F_f)$ is the volume coefficient of F_f, equal to the volume of an oriented tetrahedron formed by u_f, v_f, w_f and the origin. It is calculated via the determinant formula:

$$\mathrm{vol}\left(F_f\right) = \frac{1}{6}\det\begin{vmatrix} u_{f\mathrm{x}} & u_{f\mathrm{y}} & u_{f\mathrm{z}} \\ v_{f\mathrm{x}} & v_{f\mathrm{y}} & v_{f\mathrm{z}} \\ w_{f\mathrm{x}} & w_{f\mathrm{y}} & w_{f\mathrm{z}} \end{vmatrix}. \tag{8}$$

The term S_{ijk} needs three real arrays S, D and C with size $(N+1) \times (N+1) \times (N+1)$:

$$S_{i,j,k}\left(F_f\right) = u_{f\mathrm{x}}S_{i-1,j,k} + u_{f\mathrm{y}}S_{i,j-1,k} + u_{f\mathrm{z}}S_{i,j,k-1} + D_{i,j,k}\left(F_f\right), \tag{9a}$$

$$D_{i,j,k}\left(F_f\right) = v_{f\mathrm{x}}D_{i-1,j,k} + v_{f\mathrm{y}}D_{i,j-1,k} + v_{f\mathrm{z}}D_{i,j,k-1} + C_{i,j,k}\left(F_f\right), \tag{9b}$$

$$C_{i,j,k}\left(F_f\right) = w_{f\mathrm{x}}C_{i-1,j,k} + w_{f\mathrm{y}}C_{i,j-1,k} + w_{f\mathrm{z}}C_{i,j,k-1}, \tag{9c}$$

where $S_{0,0,0} = D_{0,0,0} = C_{0,0,0} = 1$ and $S_{i,j,k} = D_{i,j,k} = C_{i,j,k} = 0$ if $i < 0$ or $j < 0$ or $k < 0$.

S5.4. The Zernike–Canterakis Moments

Allocate five complex arrays V, W, X, Y, Z with size $(N+1) \times (N+1) \times (N+1)$. The first four arrays are intermediate, needed to calculate the ZC moments from the geometric moments from array G. The output of the algorithm is in array Z.

Populate arrays V, W and X at indices a, b, c using the following formulas:

$$V_{a,b,c}(G) = \sum_{j=0}^{a+c}\left[(i)^j\binom{a+c}{j}G_{2a+c-j,j,b}\right], \tag{10a}$$

$$W_{a,b,c}(G) = \sum_{j=0}^{a}\left[(-1)^j 2^{a-j}\binom{a}{j}V_{a-j,b,c+2j}\right], \tag{10b}$$

$$X_{a,b,c}(G) = \sum_{j=0}^{a}\left[\binom{a}{j}W_{a-j,b+2j,c}\right], \tag{10c}$$

where i is the imaginary unit and $0 \leq a, b, c \leq N$, subject to $2a + b + c \leq N$.

Populate array Y at indices l, v, m using the following formula:

$$Y_{l,v,m}(G) = \sum_{j=0}^{(l-m)/2} \left[(-1)^j \frac{\sqrt{2l+1}}{2^l} \frac{(m|j|l-m-2j)}{\sqrt{(m|m|l-m)}} \binom{2(l-j)}{l-j} X_{v+j,l-m-2j,m} \right], \tag{11}$$

where $0 \leq l, v, m \leq N$, subject to $0 \leq m \leq l \leq N$ and $0 \leq v \leq (N-l)/2$. Note: Equation (11) is a combination of Equations (12) and (13d) from the paper of Pozo et al.

$(a|b|c)$ is the trinomial coefficient. It can be calculated directly with factorials or via Pascal's pyramid (an extension of Pascal's triangle) by exploiting its recursion property:

$$(a|b|c) = \frac{(a+b+c)!}{a!\,b!\,c!} = (a-1|b|c) + (a|b-1|c) + (a|b|c-1), \tag{12}$$

where $(0|0|0) = 1$ and $(a|b|c) = 0$ if $a < 0$ or $b < 0$ or $c < 0$. Observe that the fraction in Equation (7) can also be written as $[(i|j|k)(n+1)(n+2)(n+3)]^{-1}$, where $n = i + j + k$.

Populate array Z at indices n, l, m using the following formula:

$$Z_{n,l,m}(G) = \frac{3}{4\pi} \sum_{v=0}^{k} \left[\frac{(-1)^{k+v}}{4^k} \frac{\sqrt{2l+4k+3}}{3} (v|k-v|l+v+1) \frac{\binom{2(l+v+1+k)}{l+v+1+k}}{\binom{2(l+v+1)}{l+v+1}} \overline{Y_{l,v,m}} \right], \tag{13}$$

where $k = (n-l)/2$ and $0 \leq n, l, m \leq N$, subject to $0 \leq m \leq l \leq n \leq N$ and $(n-l)\,\%\,2 = 0$. $\overline{Y_{l,v,m}}$ is the complex conjugate of $Y_{l,v,m}$ and % is the modulo operator. Note: the term $Q_{k,l,v}$ from the paper of Pozo et al. (unlabeled equation) is included directly in Equation (13).

S5.5. The Zernike–Canterakis Descriptors

The Zernike–Canterakis descriptors (ZCDs) are compact, low-dimensional representatives of the ZC moments. They are invariant to the rotations of the input shape. Note that the PK algorithm ends at Equation (13). What follows is the description of the so-called 3D Zernike descriptor (3DZD), a ZCD formula popularized by M. Novotni and R. Klein.

Iterate the n, l indices of array Z for which $0 \leq l \leq n \leq N$ and $(n-l)\,\%\,2 = 0$. Again, % is the modulo operator. Construct vector $F_{n,l}(Z)$ for each matching index pair:

$$F_{n,l}(Z) = [Z_{n,l,-l} \ldots Z_{n,l,0} \ldots Z_{n,l,+l}]. \tag{14}$$

The moments with the negative m are obtained via simple symmetric relation:

$$Z_{n,l,-m} = \overline{Z_{n,l,m}}(-1)^m. \tag{15}$$

The norms of the $F_{n,l}(Z)$ vectors, $|F_{n,l}(Z)|$, are the Zernike–Canterakis descriptors. Together they constitute $F_N(Z)$, the Zernike–Canterakis descriptor vector (ZCDV):

$$F_N(Z) = \Big[|F_{0,0}| \ldots |F_{N,N}| \Big]. \tag{16}$$

The number of ZCDs at moment order N—the number of components of $F_N(Z)$—is given by the following formula:

$$\#F_N(Z) = \left\lfloor \frac{(N+2)^2}{4} \right\rfloor. \tag{17}$$

For example, a ZCDV of M at $N = 20$ has 121 ZCDs. At $N = 30$ there is 256 of them.
