# Supplementary material for: Structural Outlier Detection and Zernike–Canterakis Moments for Molecular Surface Meshes—Fast Implementation in Python
Source: Molecules. 2023 Dec 21;29(1):52. doi: 10.3390/molecules29010052 (PMC10779519; doi:10.3390/molecules29010052)

Figure S15. BioZernike validation — all atoms mesh, r_{max}, outlier detection off

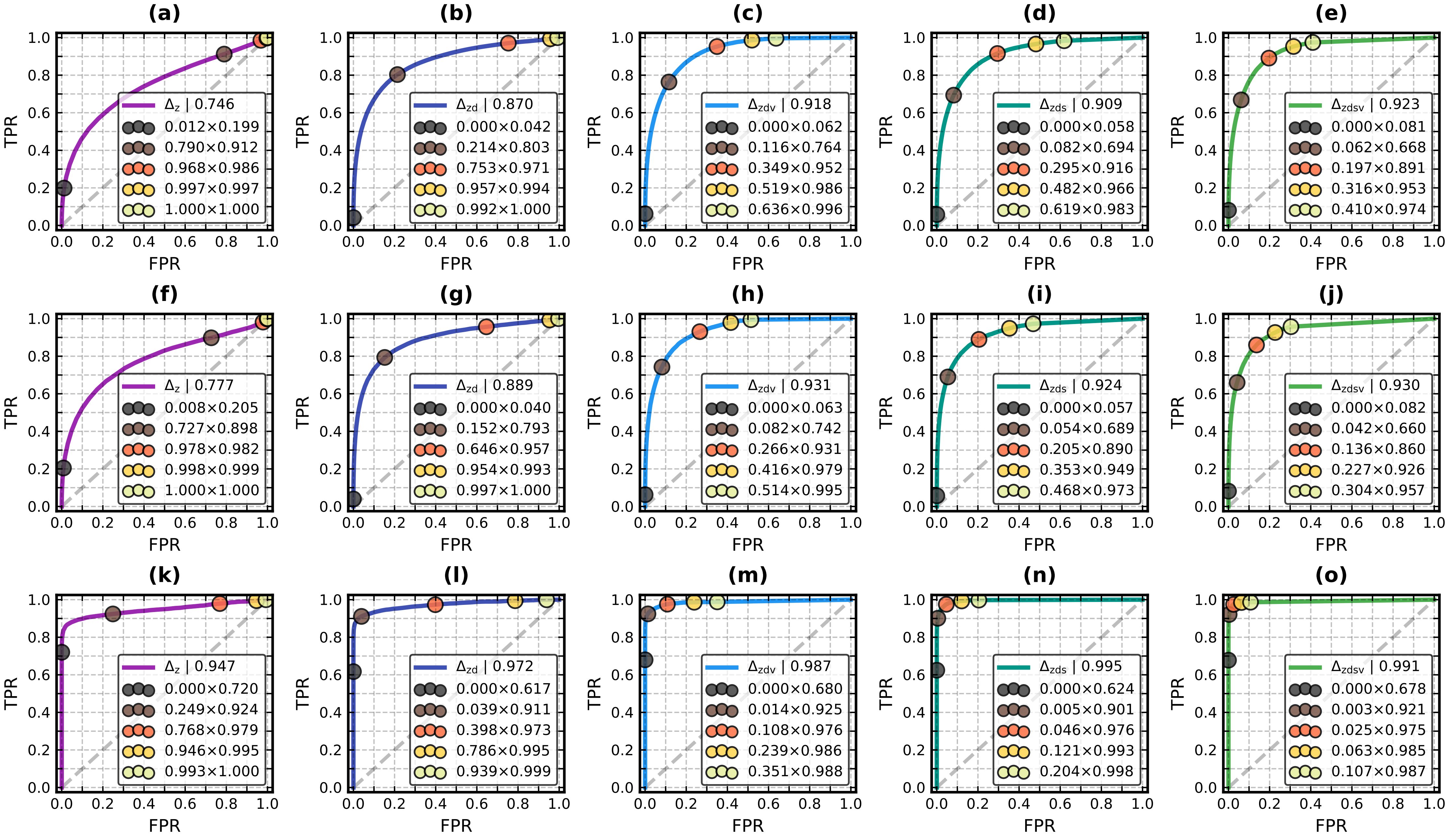

Figure S16. BioZernike validation — all atoms mesh, r_{max}, outlier detection on

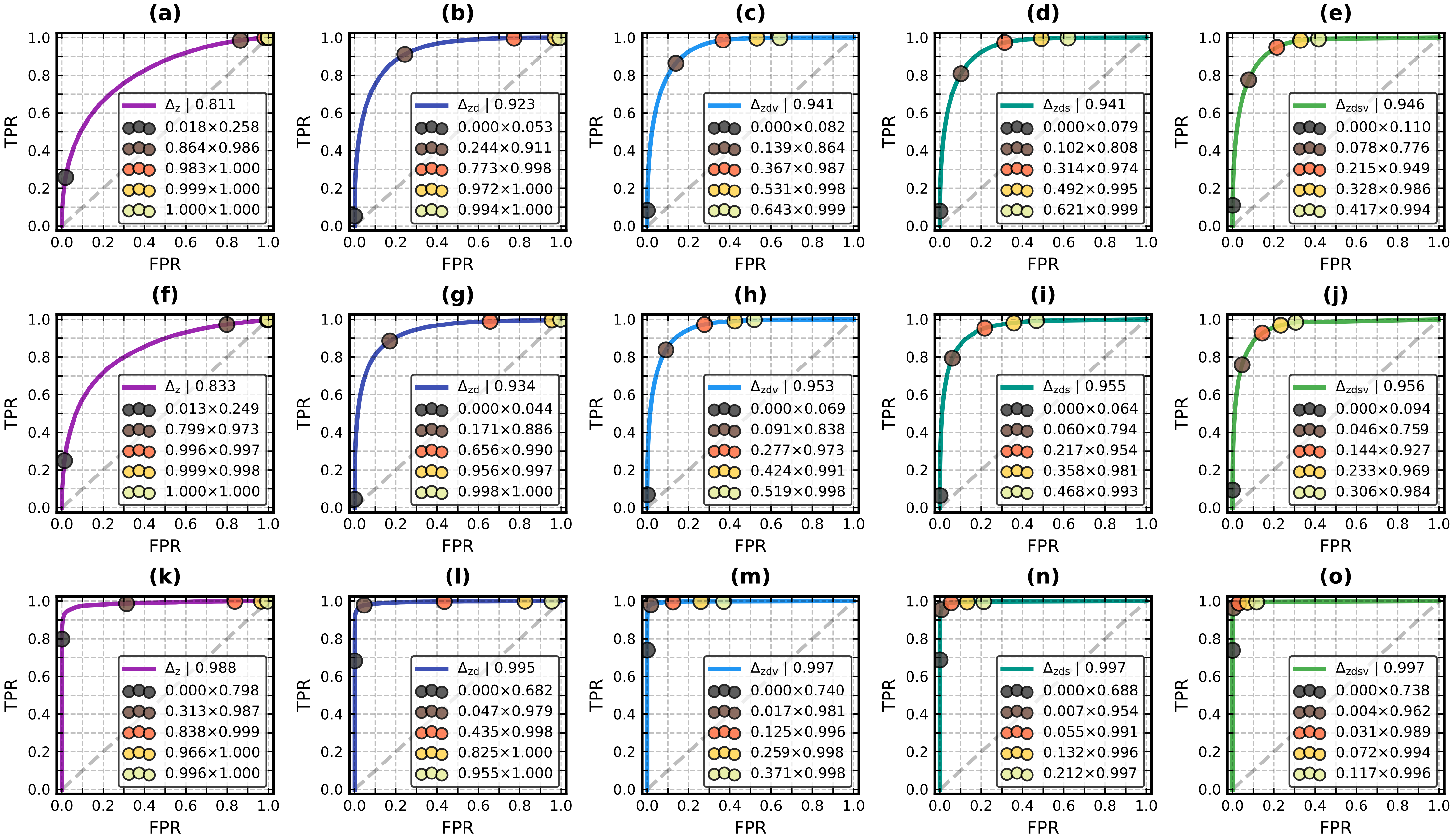

Figure S17. BioZernike validation — all atoms mesh, $r_{max}/0.7$, outlier detection off

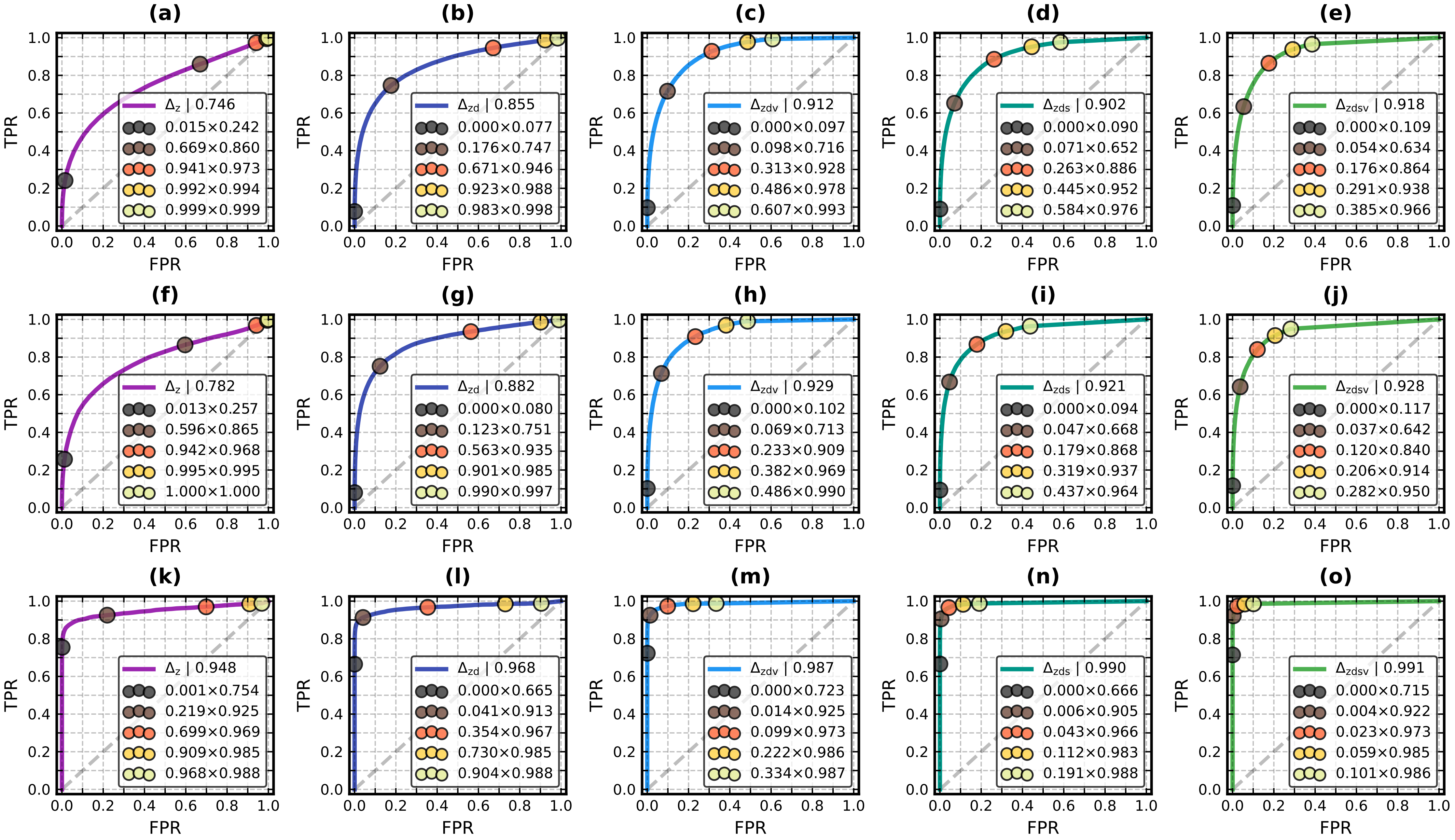

Figure S18. BioZernike validation — all atoms mesh, $r_{max}/0.7$, outlier detection on

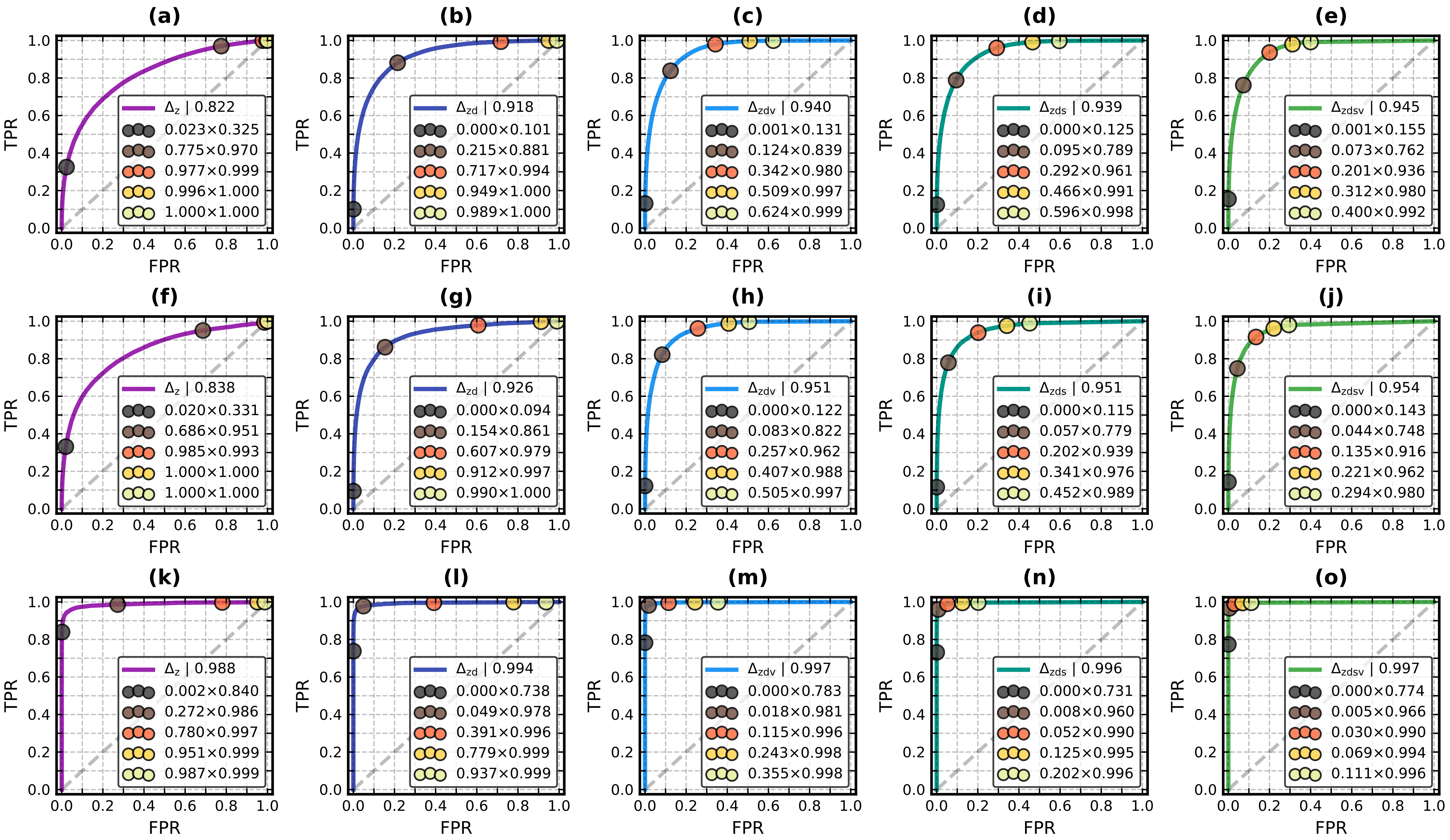

Figure S19. BioZernike validation — all atoms mesh, $2r_g$, outlier detection off

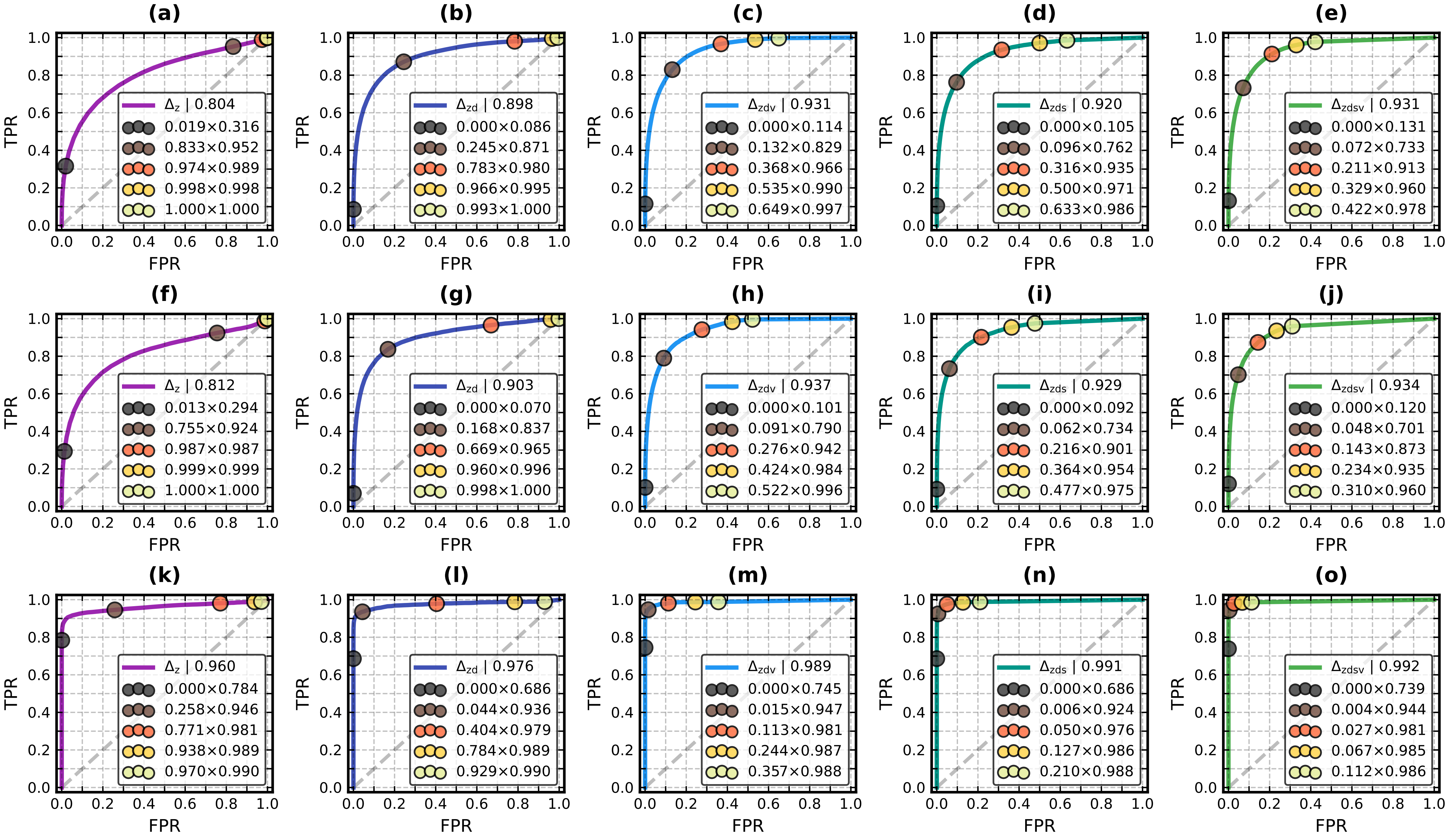

Figure S20. BioZernike validation — all atoms mesh, $2r_g$, outlier detection on

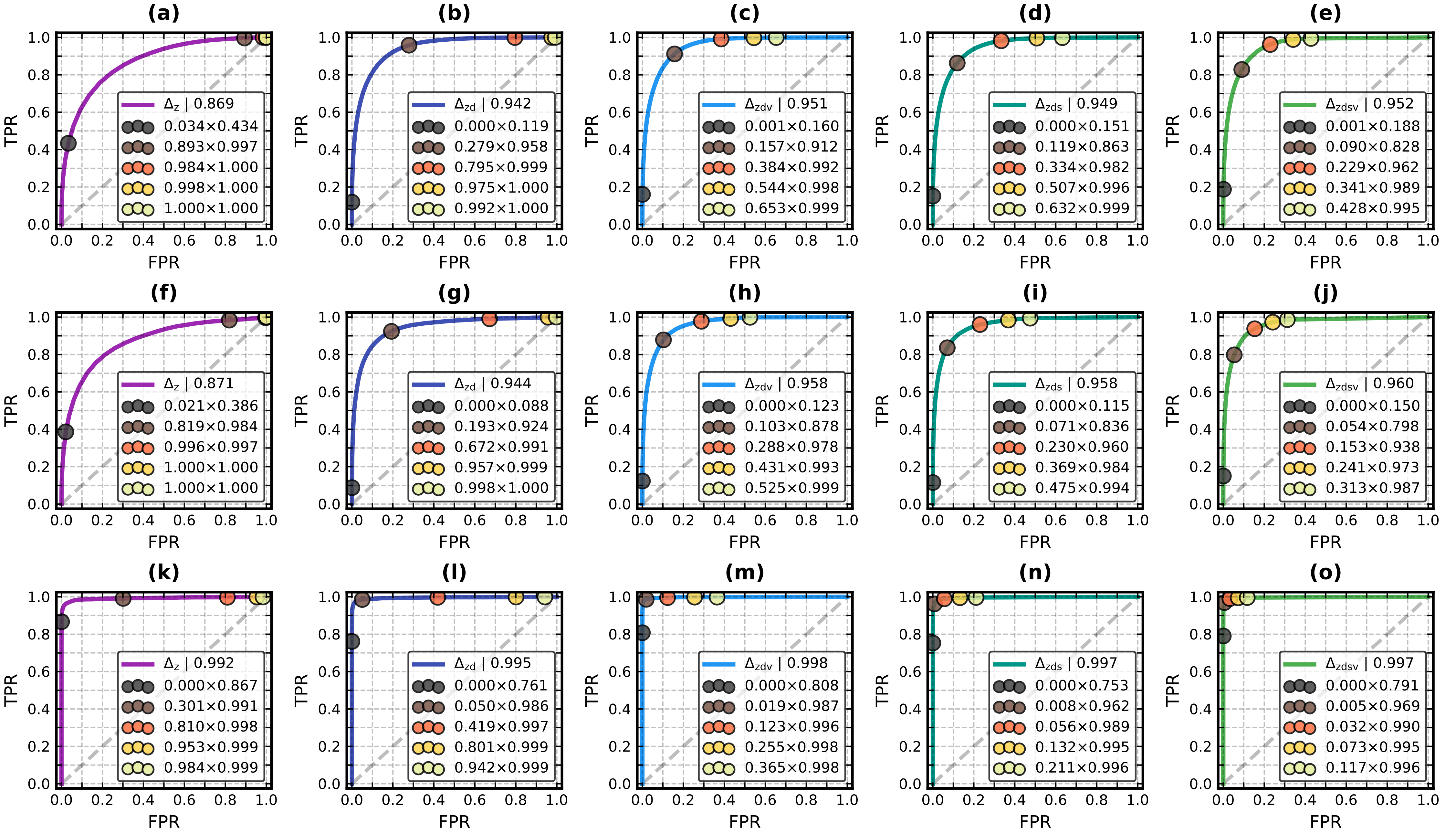

Figure S21. BioZernike validation — all atoms mesh, r_{PCA}, outlier detection off

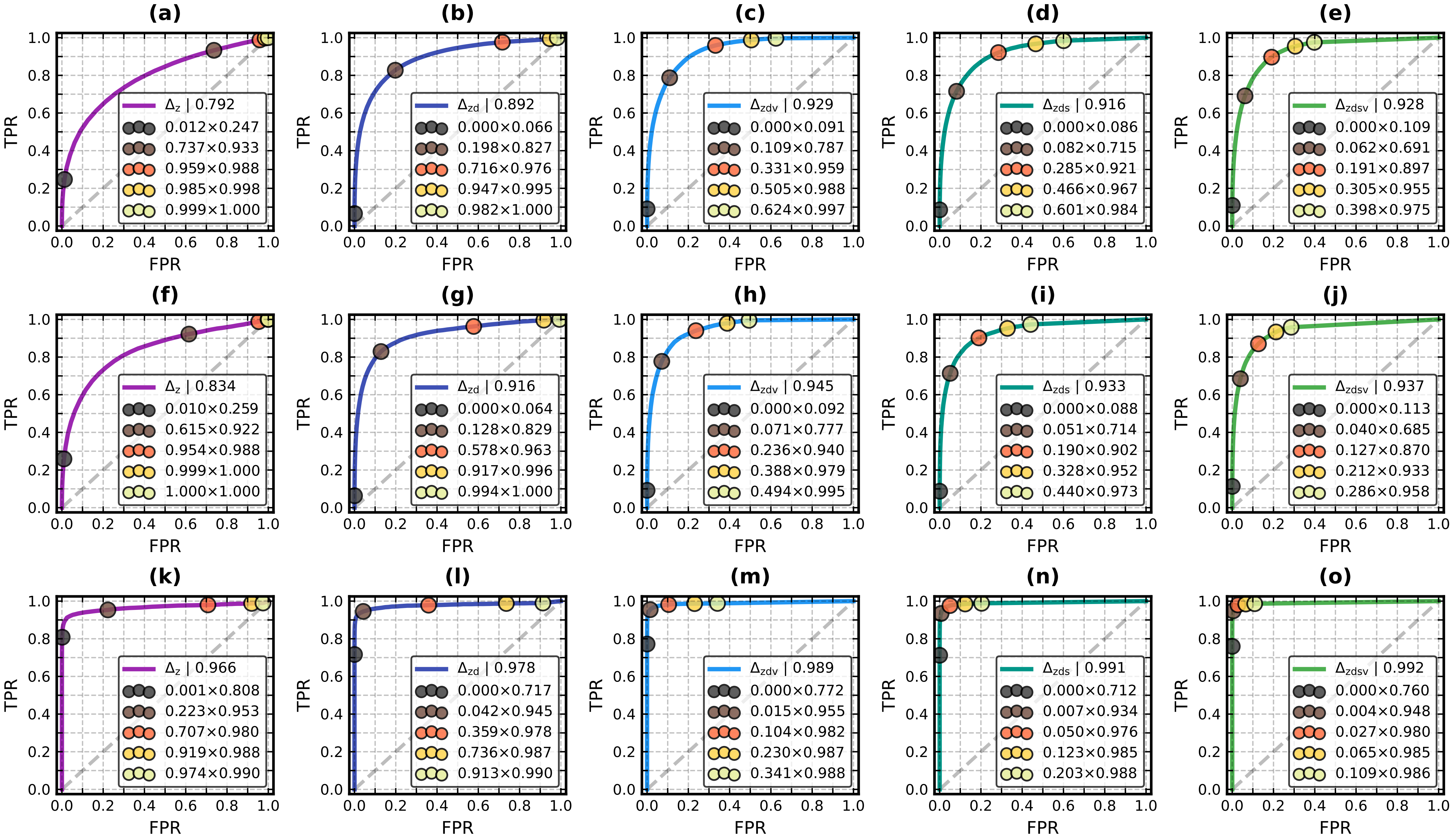

Figure S22. BioZernike validation — all atoms mesh, r_{PCA}, outlier detection on

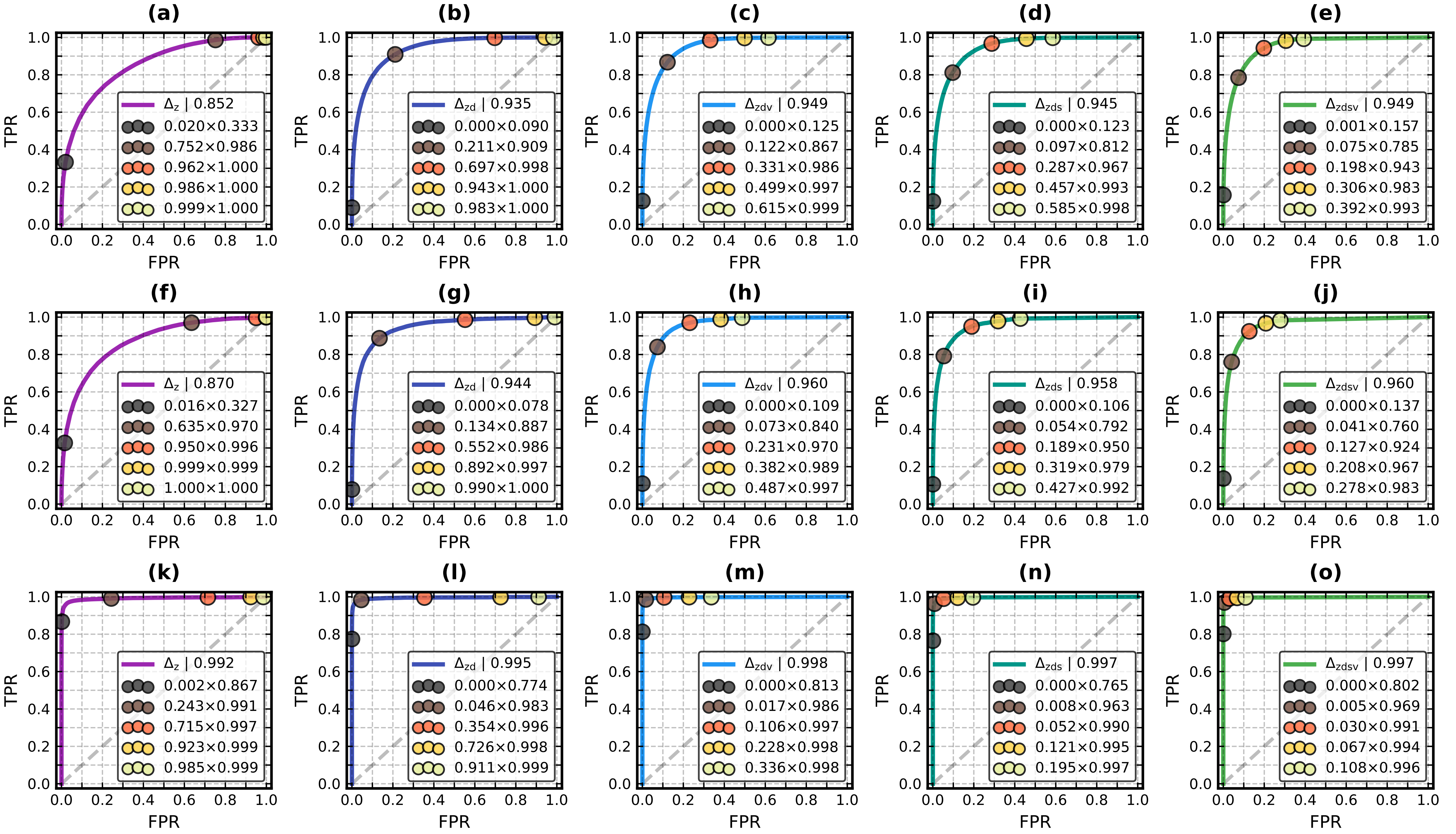

Figure S23. BioZernike validation — backbone atoms mesh, r_{max}, outlier detection off

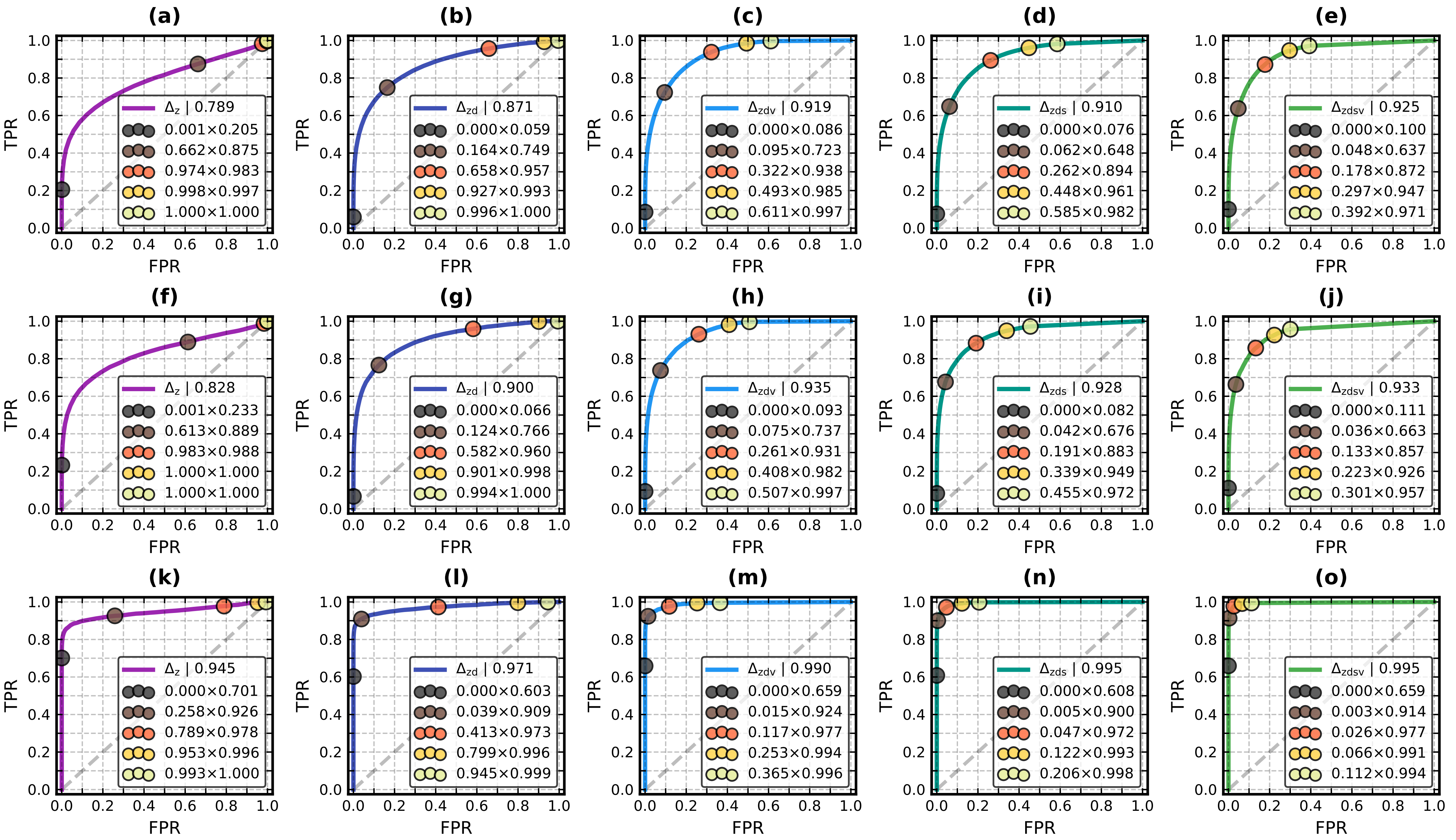

Figure S24. BioZernike validation — backbone atoms mesh, r_{max}, outlier detection on

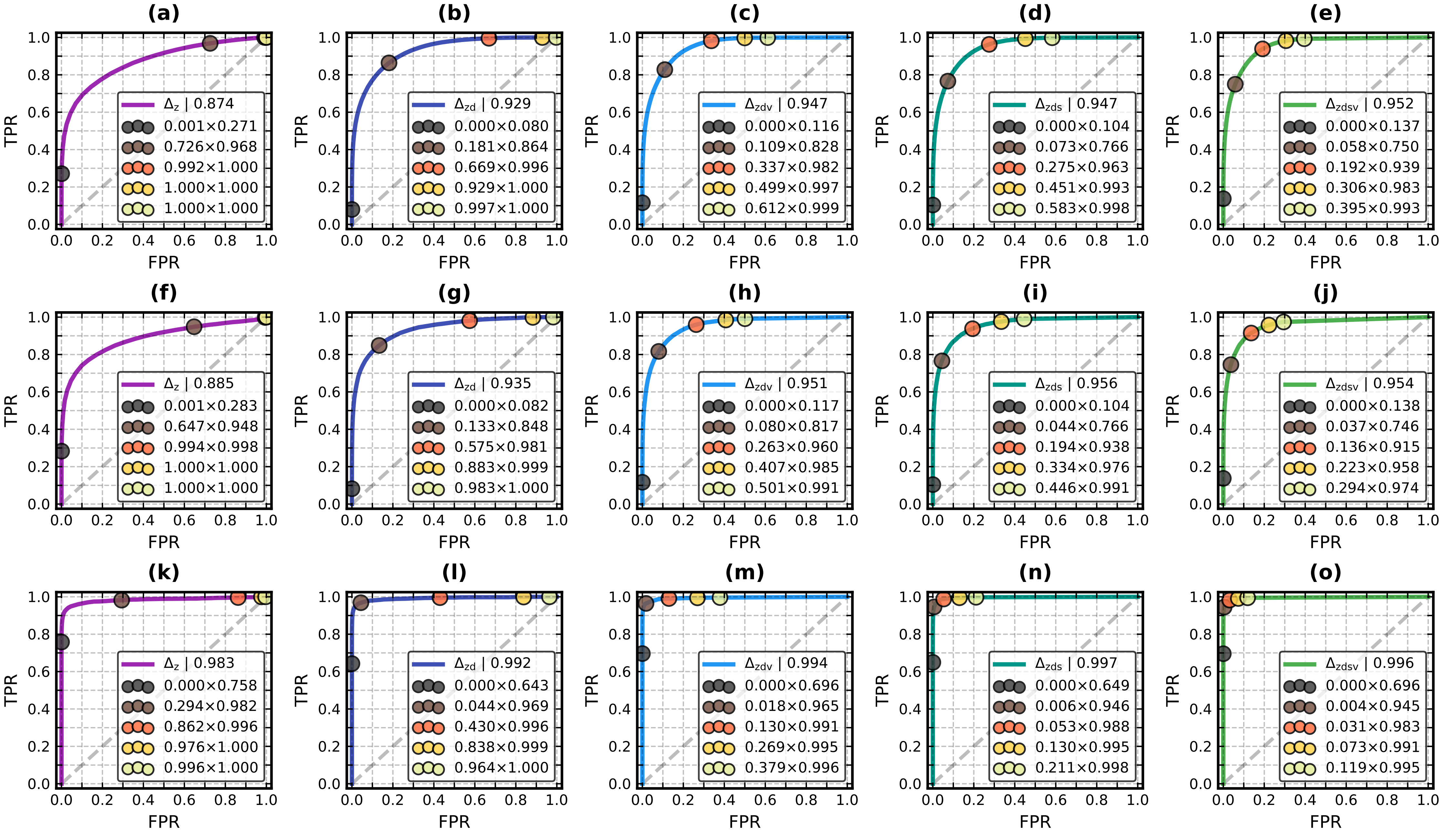

Figure S25. BioZernike validation — backbone atoms mesh, $r_{max}/0.7$, outlier detection off

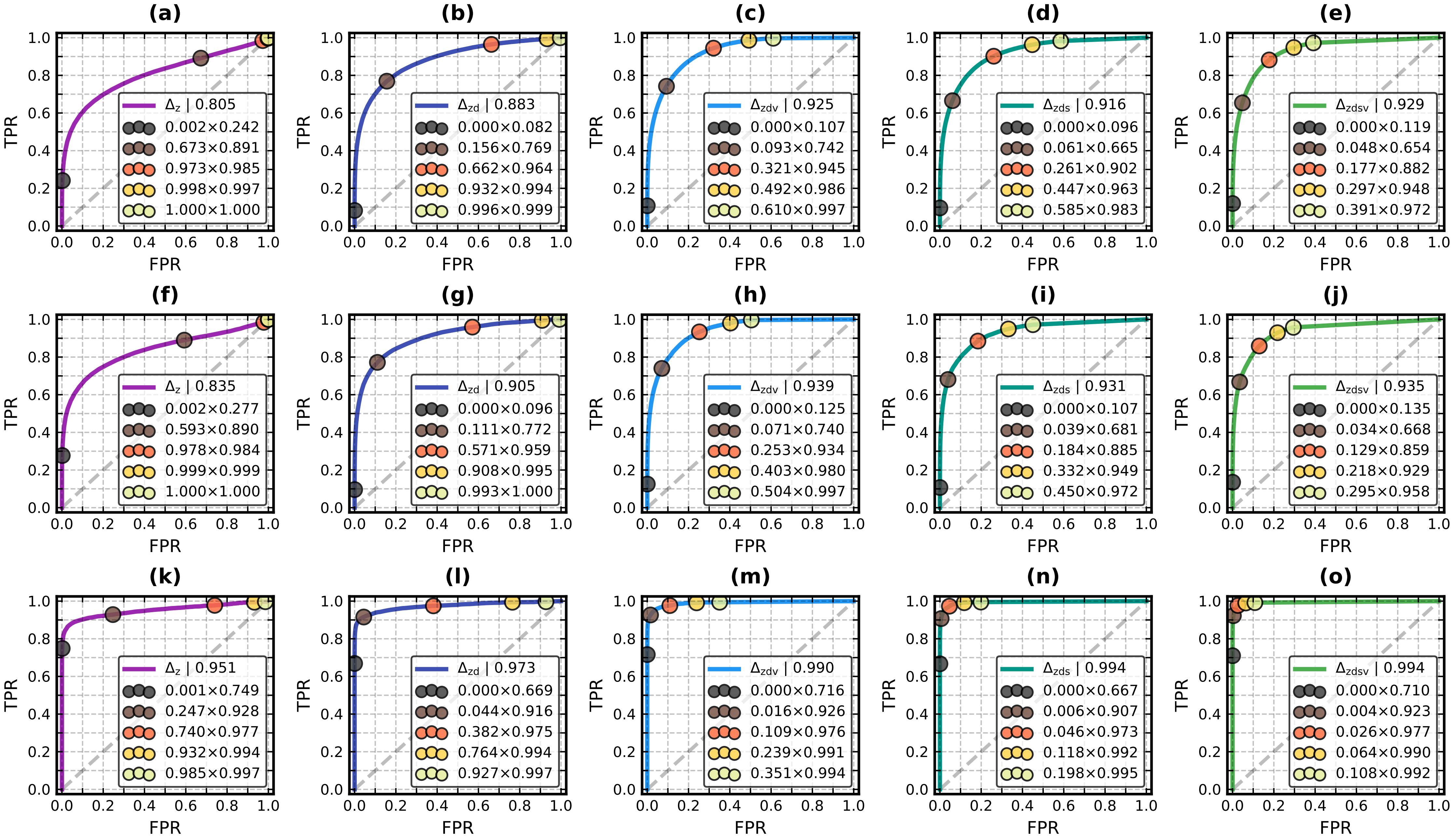

Figure S26. BioZernike validation — backbone atoms mesh, $r_{max}/0.7$, outlier detection on

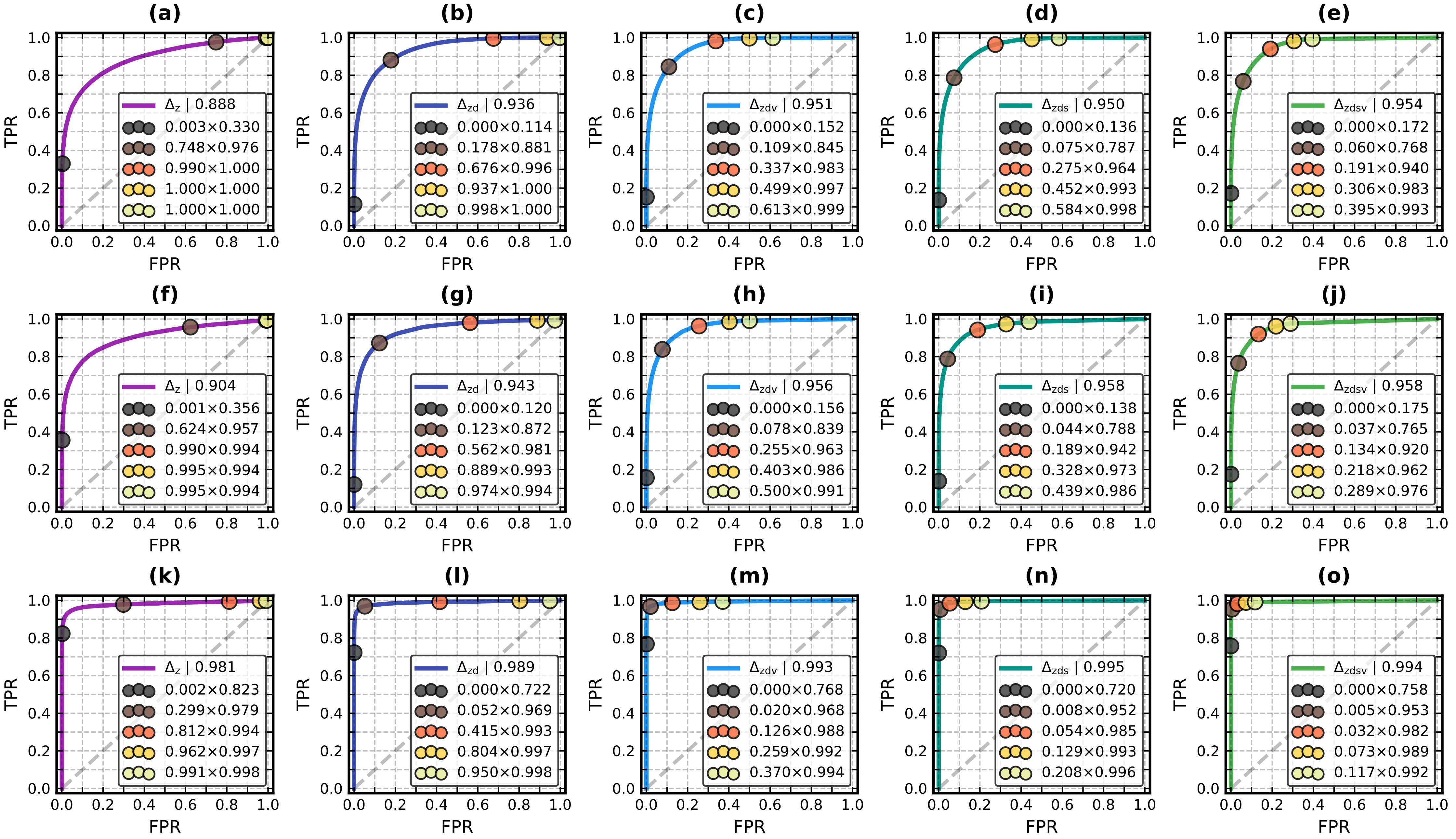

Figure S27. BioZernike validation — backbone atoms mesh, $2r_g$, outlier detection off

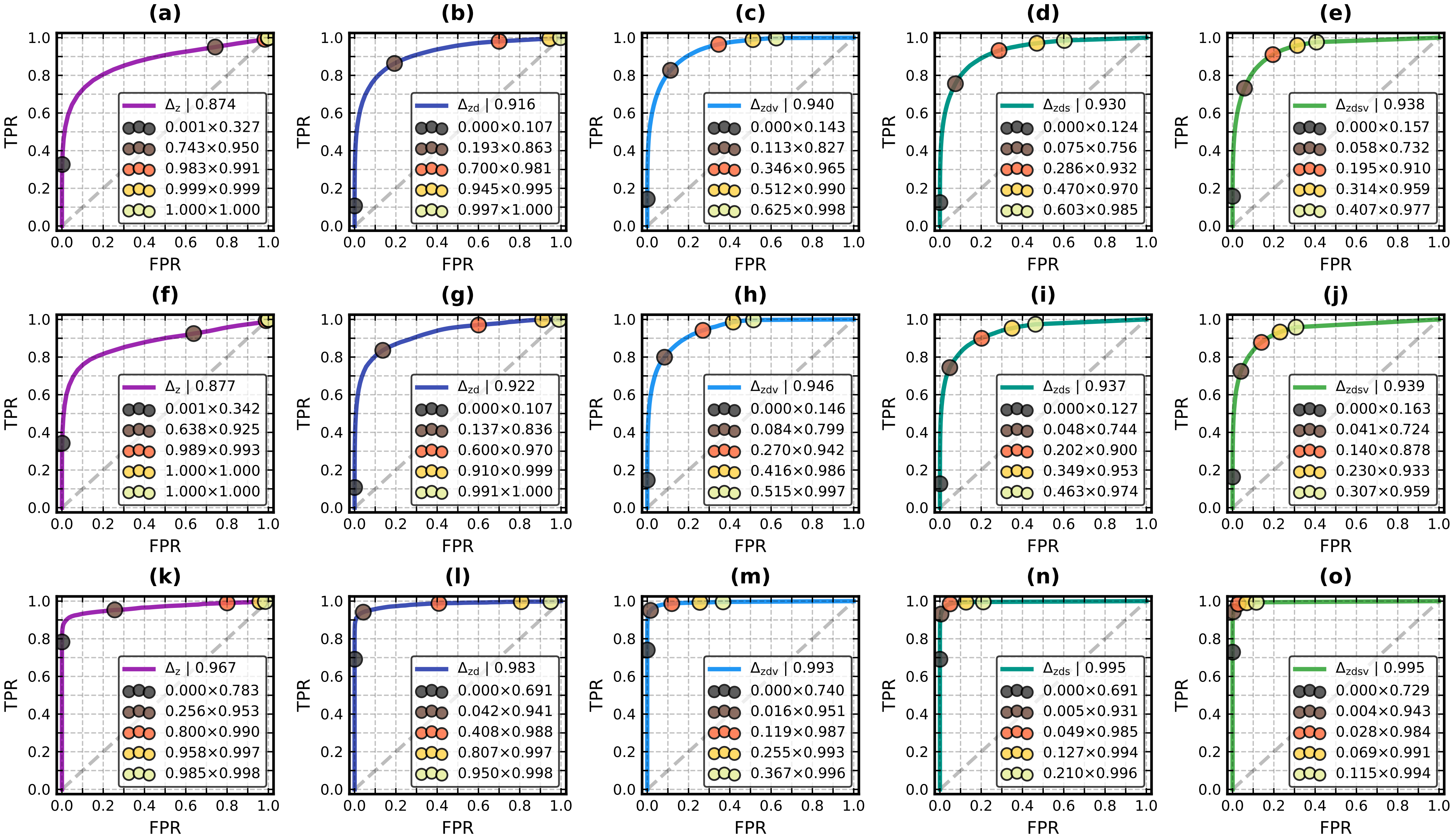

Figure S28. BioZernike validation — backbone atoms mesh, $2r_g$, outlier detection on

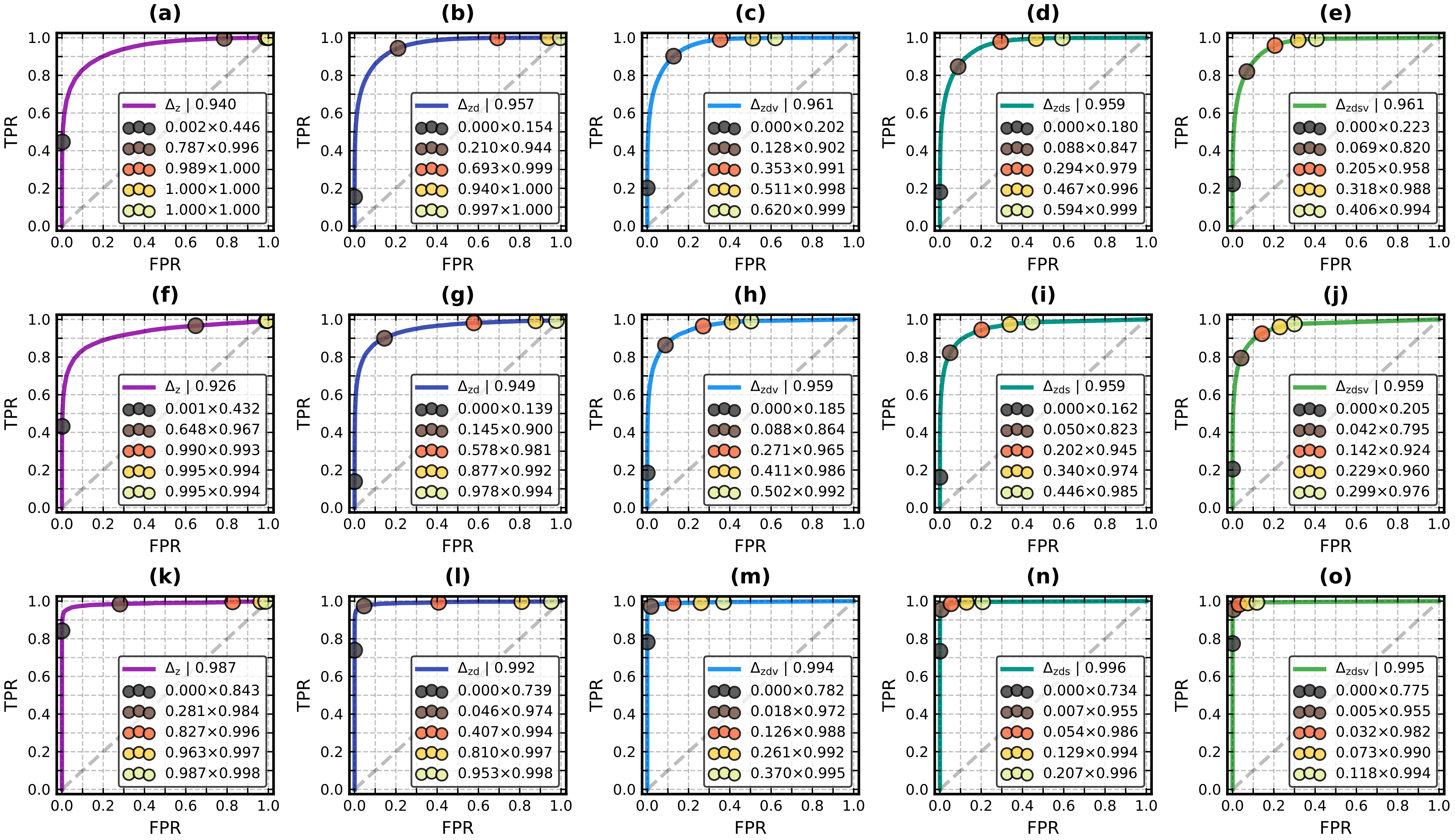

Figure S29. BioZernike validation — backbone atoms mesh, r_{PCA}, outlier detection off

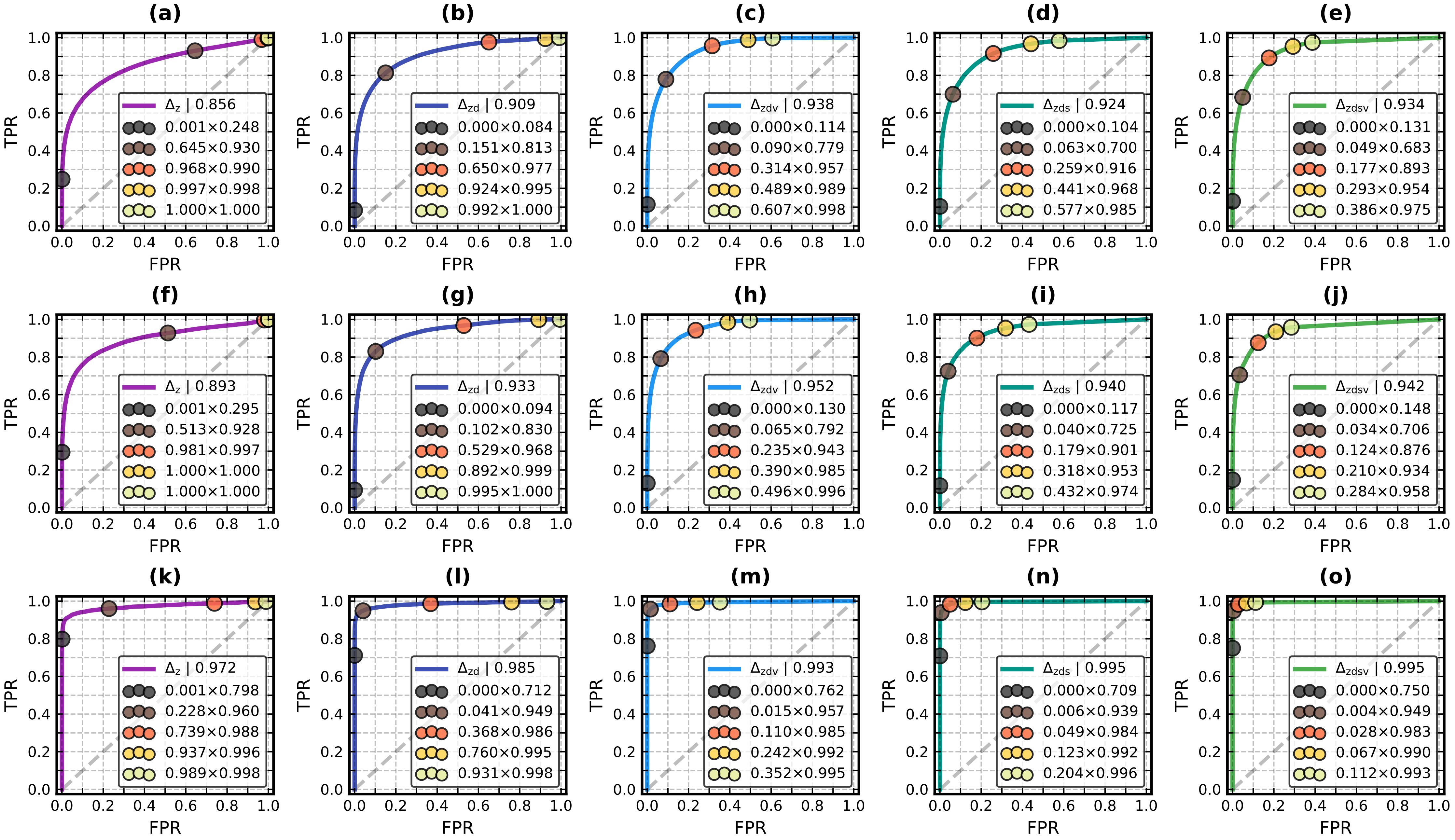

Figure S30. BioZernike validation — backbone atoms mesh, r_{PCA}, outlier detection on

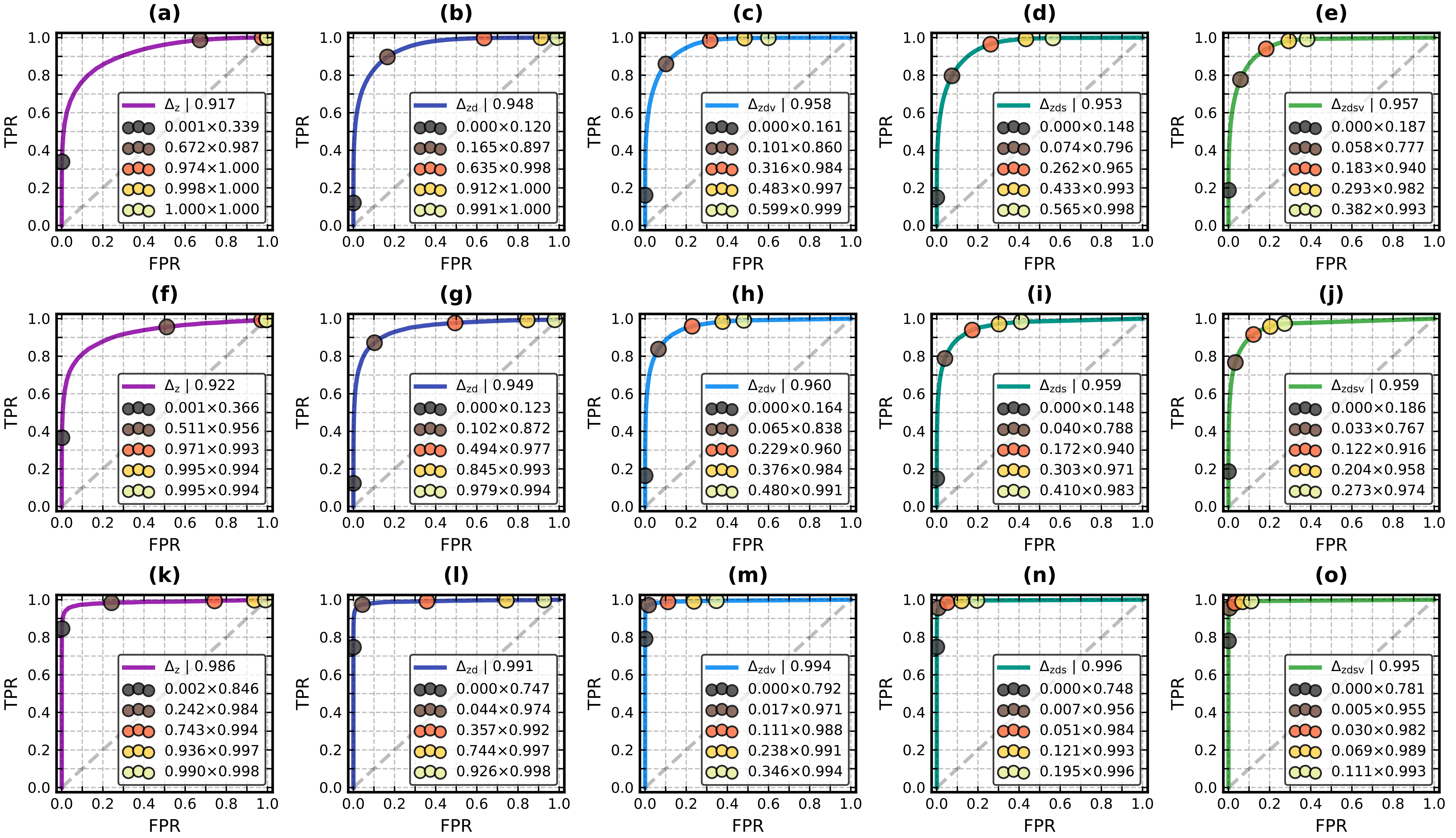

Supplement: Supplementary file 1 [file molecules-29-00052-s001.zip › supplement2.pdf]
