# Supplementary material for: Structural Outlier Detection and Zernike–Canterakis Moments for Molecular Surface Meshes—Fast Implementation in Python
Source: Molecules. 2023 Dec 21;29(1):52. doi: 10.3390/molecules29010052 (PMC10779519; doi:10.3390/molecules29010052)
Supplement: Supplementary file 1 [file molecules-29-00052-s001.zip › supplement3.pdf]

Figure S31. Example proteins — backbone atoms mesh, outlier detection on, r_{PCA}, Δ_z

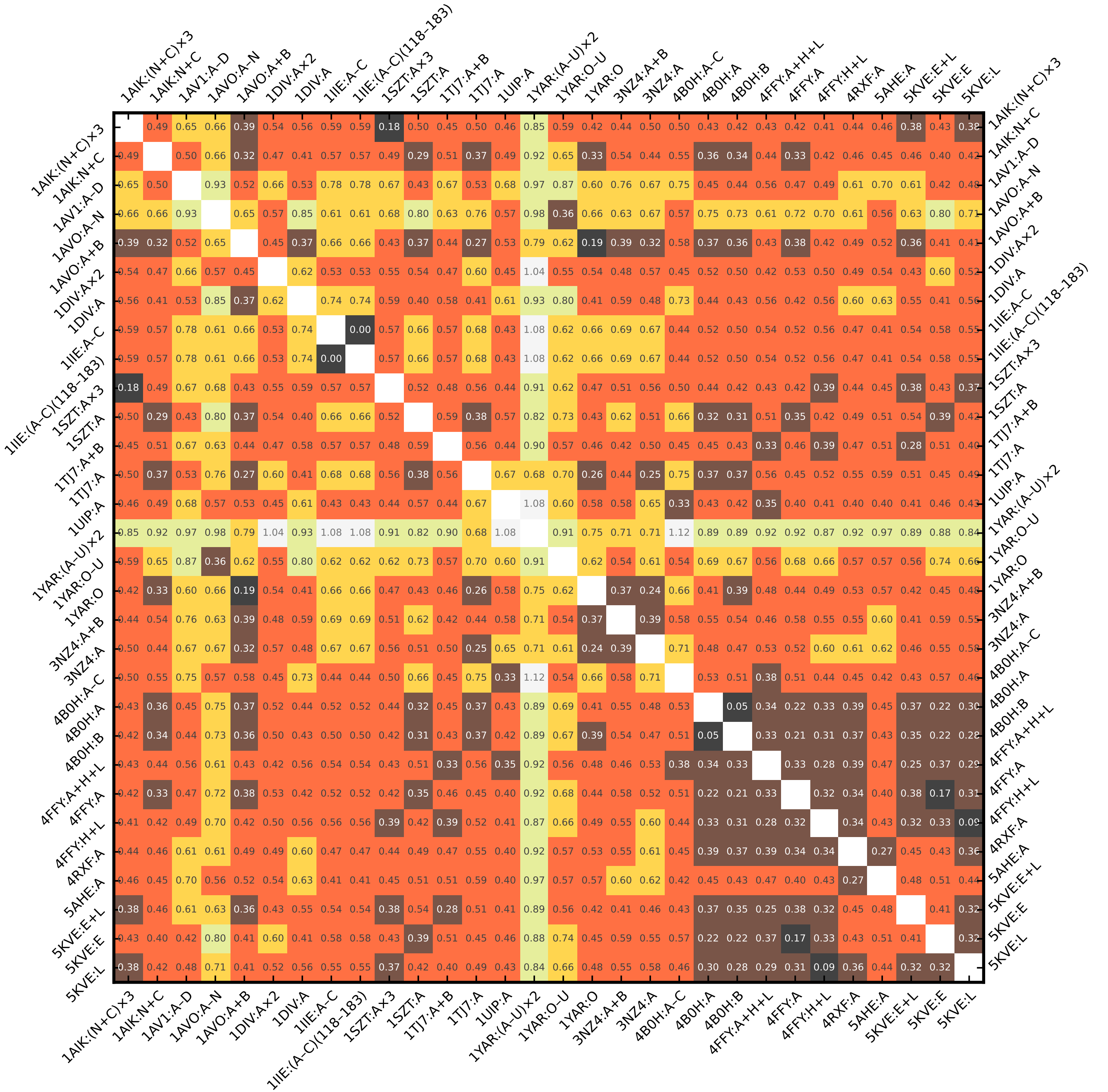

Figure S32. Example proteins — backbone atoms mesh, outlier detection on, r_{PCA}, Δ_{zd}

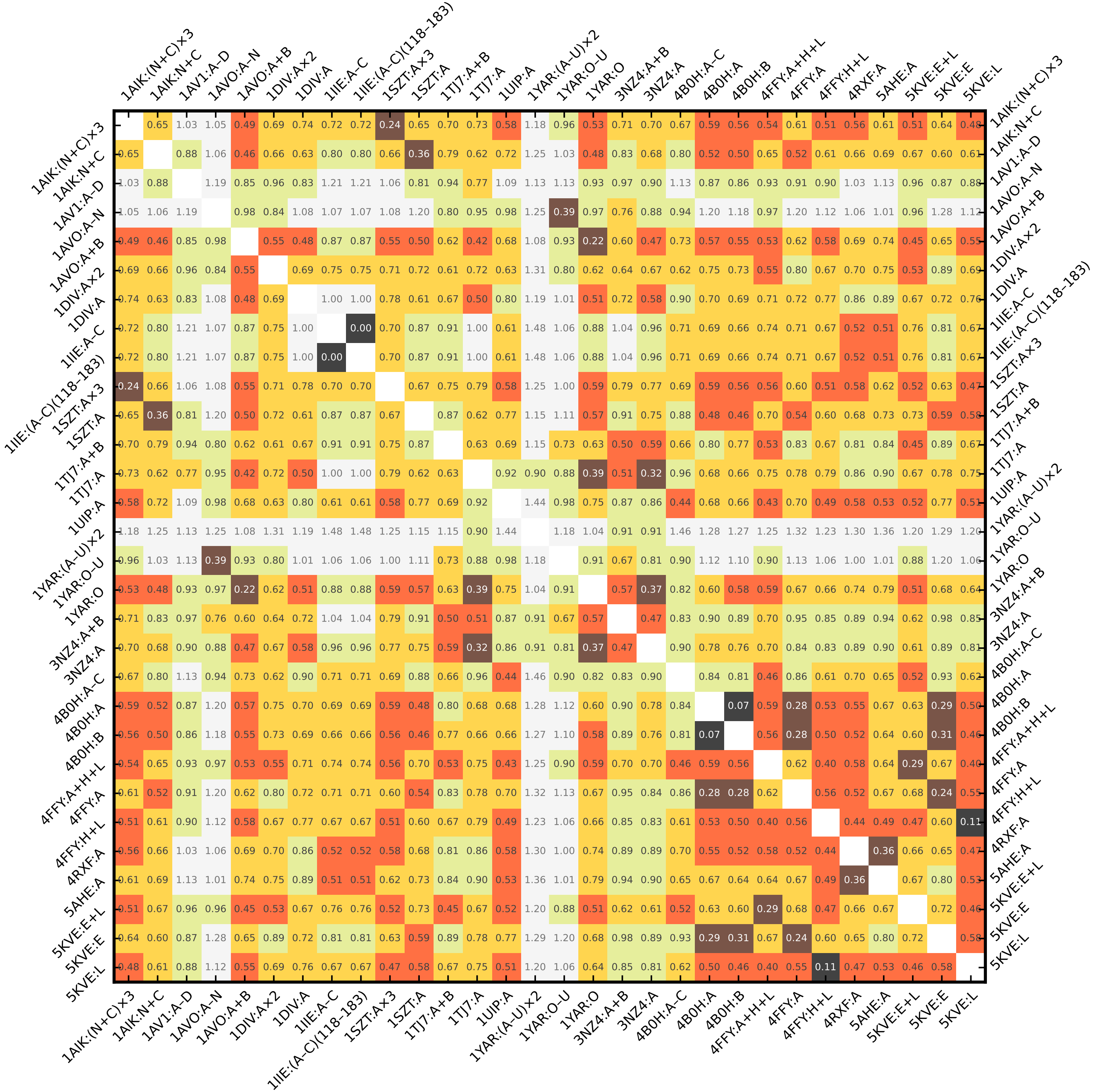

Figure S33. Example proteins — backbone atoms mesh, outlier detection on, r_{PCA}, Δ_{zdv}

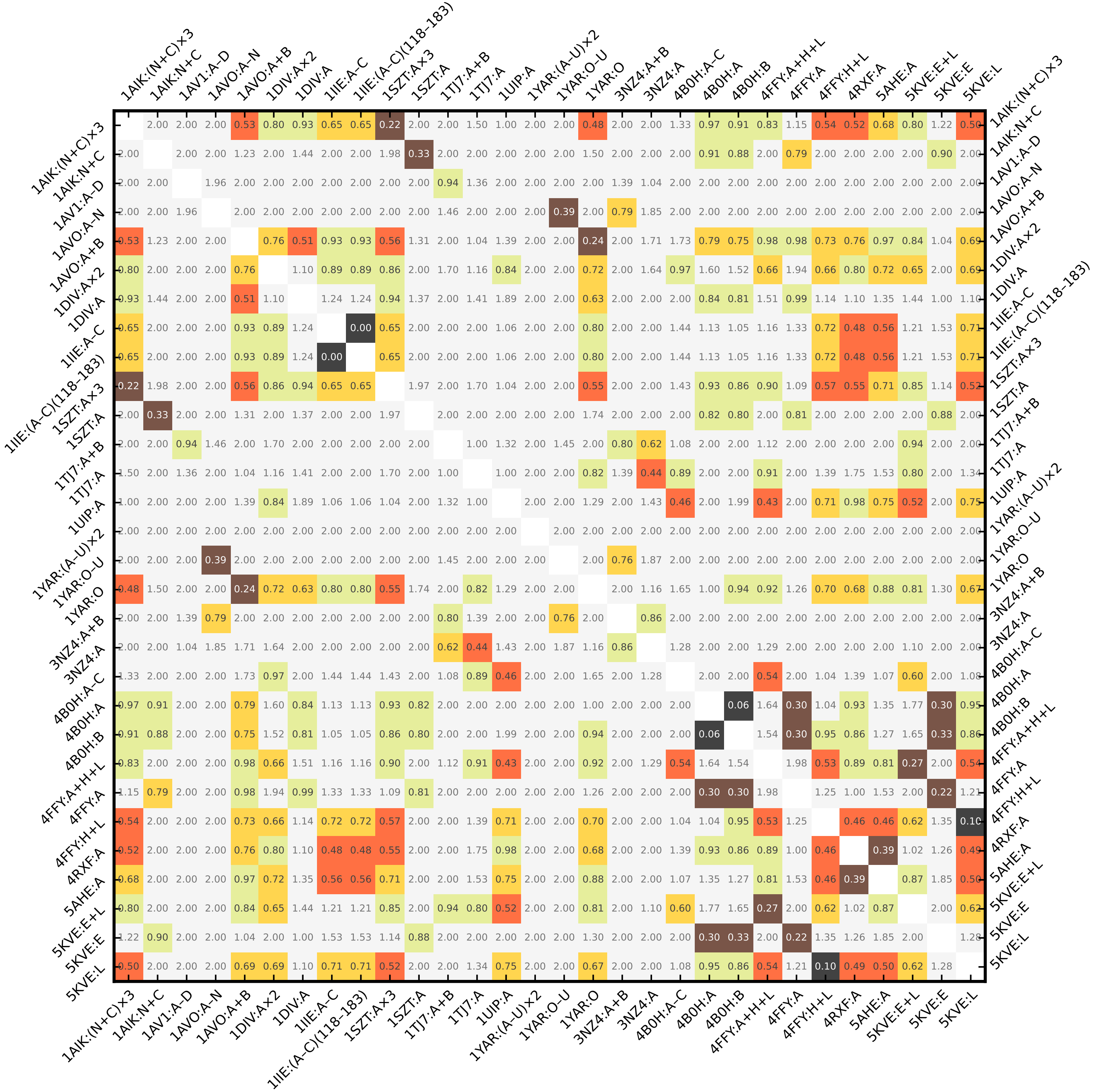

Figure S34. Example proteins — backbone atoms mesh, outlier detection on, r_{PCA}, Δ_{zds}

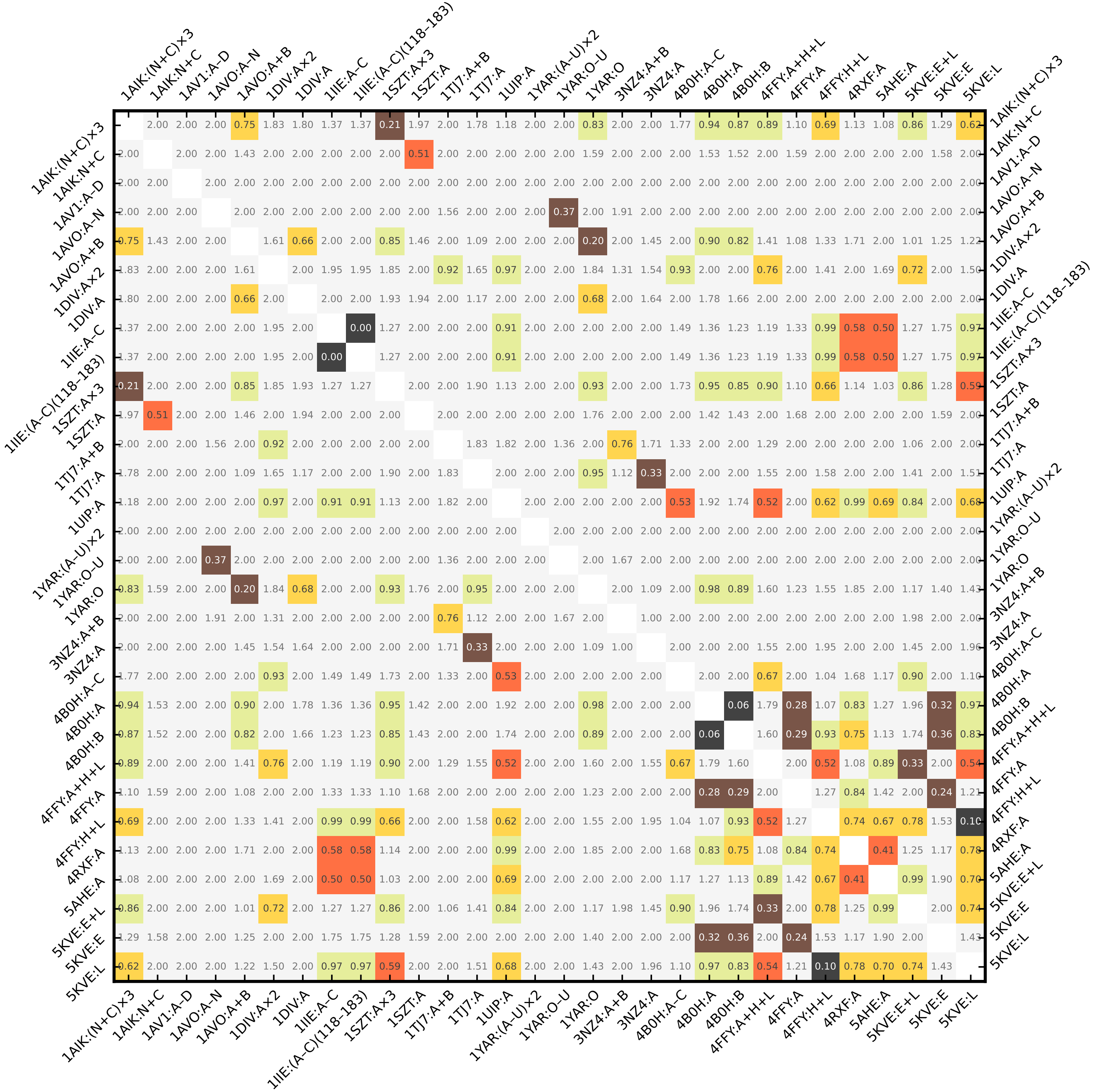

Figure S35. Example proteins — backbone atoms mesh, outlier detection on, r_{PCA}, Δ_{zdsv}

	1AIK:(N+C)x3	1AIK:N+C	1AV1:A–D	1AVO:A–N	1AVO:A+B	1DIV:Ax2	1DIV:A	1IIE:A–C	1IIE:(A–C)(118–183)	1SZT:Ax3	1SZT:A	1TJ7:A+B	1TJ7:A	1UIP:A	1YAR:(A–U)x2	1YAR:O–U	1YAR:O	3NZ4:A+B	3NZ4:A	4B0H:A–C	4B0H:A	4B0H:B	4FFY:A+H+L	4FFY:A	4FFY:H+L	4RXF:A	5AHE:A	5KVE:E+L	5KVE:E	5KVE:L
1AIK:(N+C)x3		2.00	2.00	2.00	0.81	2.00	2.00	1.25	1.25	0.20	2.00	2.00	2.00	2.00	2.00	2.00	0.75	2.00	2.00	2.00	1.55	1.41	1.38	2.00	0.74	1.04	1.19	1.35	2.00	0.65
1AIK:N+C	2.00		2.00	2.00	2.00	2.00	2.00	2.00	2.00	2.00	0.46	2.00	2.00	2.00	2.00	2.00	2.00	2.00	2.00	2.00	2.00	2.00	2.00	2.00	2.00	2.00	2.00	2.00	2.00	2.00
1AV1:A–D	2.00	2.00		2.00	2.00	2.00	2.00	2.00	2.00	2.00	2.00	2.00	2.00	2.00	2.00	2.00	2.00	2.00	2.00	2.00	2.00	2.00	2.00	2.00	2.00	2.00	2.00	2.00	2.00	2.00
1AVO:A–N	2.00	2.00	2.00		2.00	2.00	2.00	2.00	2.00	2.00	2.00	2.00	2.00	2.00	2.00	0.37	2.00	1.98	2.00	2.00	2.00	2.00	2.00	2.00	2.00	2.00	2.00	2.00	2.00	2.00
1AVO:A+B	0.81	2.00	2.00	2.00		2.00	0.70	2.00	2.00	0.87	2.00	2.00	2.00	2.00	2.00	2.00	0.22	2.00	2.00	2.00	1.26	1.12	2.00	1.72	1.70	1.87	2.00	1.88	2.00	1.53
1DIV:Ax2	2.00	2.00	2.00	2.00	2.00		2.00	2.00	2.00	2.00	2.00	2.00	2.00	1.29	2.00	2.00	2.00	2.00	2.00	1.44	2.00	2.00	0.91	2.00	1.39	2.00	1.61	0.87	2.00	1.49
1DIV:A	2.00	2.00	2.00	2.00	0.70	2.00		2.00	2.00	2.00	2.00	2.00	2.00	2.00	2.00	2.00	0.85	2.00	2.00	2.00	2.00	1.94	2.00	2.00	2.00	2.00	2.00	2.00	2.00	2.00
1IIE:A–C	1.25	2.00	2.00	2.00	2.00	2.00	2.00		0.00	1.18	2.00	2.00	2.00	1.58	2.00	2.00	2.00	2.00	2.00	2.00	2.00	1.96	1.87	2.00	1.07	0.54	0.56	2.00	2.00	1.03
1IIE:(A–C)(118–183)	1.25	2.00	2.00	2.00	2.00	2.00	2.00	0.00		1.18	2.00	2.00	2.00	1.58	2.00	2.00	2.00	2.00	2.00	2.00	2.00	1.96	1.87	2.00	1.07	0.54	0.56	2.00	2.00	1.03
1SZT:Ax3	0.20	2.00	2.00	2.00	0.87	2.00	2.00	1.18	1.18		2.00	2.00	2.00	2.00	2.00	2.00	0.88	2.00	2.00	2.00	1.50	1.31	1.46	1.99	0.74	1.09	1.19	1.41	2.00	0.64
1SZT:A	2.00	0.46	2.00	2.00	2.00	2.00	2.00	2.00	2.00	2.00		2.00	2.00	2.00	2.00	2.00	2.00	2.00	2.00	2.00	2.00	2.00	2.00	2.00	2.00	2.00	2.00	2.00	2.00	2.00
1TJ7:A+B	2.00	2.00	2.00	2.00	2.00	2.00	2.00	2.00	2.00	2.00	2.00		2.00	2.00	2.00	2.00	2.00	1.21	1.79	2.00	2.00	2.00	2.00	2.00	2.00	2.00	2.00	2.00	2.00	2.00
1TJ7:A	2.00	2.00	2.00	2.00	2.00	2.00	2.00	2.00	2.00	2.00	2.00	2.00		2.00	2.00	2.00	1.97	2.00	0.45	2.00	2.00	2.00	1.89	2.00	2.00	2.00	2.00	1.70	2.00	2.00
1UIP:A	2.00	2.00	2.00	2.00	2.00	1.29	2.00	1.58	1.58	2.00	2.00	2.00	2.00		2.00	2.00	2.00	2.00	2.00	0.56	2.00	2.00	0.52	2.00	0.91	1.67	0.97	0.83	2.00	1.01
1YAR:(A–U)x2	2.00	2.00	2.00	2.00	2.00	2.00	2.00	2.00	2.00	2.00	2.00	2.00	2.00	2.00		2.00	2.00	2.00	2.00	2.00	2.00	2.00	2.00	2.00	2.00	2.00	2.00	2.00	2.00	2.00
1YAR:O–U	2.00	2.00	2.00	0.37	2.00	2.00	2.00	2.00	2.00	2.00	2.00	2.00	2.00	2.00	2.00		2.00	1.90	2.00	2.00	2.00	2.00	2.00	2.00	2.00	2.00	2.00	2.00	2.00	2.00
1YAR:O	0.75	2.00	2.00	2.00	0.22	2.00	0.85	2.00	2.00	0.88	2.00	2.00	1.97	2.00	2.00	2.00		2.00	2.00	2.00	1.62	1.44	2.00	2.00	1.66	1.70	2.00	1.84	2.00	1.51
3NZ4:A+B	2.00	2.00	2.00	1.98	2.00	2.00	2.00	2.00	2.00	2.00	2.00	1.21	2.00	2.00	2.00	1.90	2.00		1.84	2.00	2.00	2.00	2.00	2.00	2.00	2.00	2.00	2.00	2.00	2.00
3NZ4:A	2.00	2.00	2.00	2.00	2.00	2.00	2.00	2.00	2.00	2.00	2.00	1.79	0.45	2.00	2.00	2.00	2.00	1.84		2.00	2.00	2.00	2.00	2.00	2.00	2.00	2.00	2.00	2.00	2.00
4B0H:A–C	2.00	2.00	2.00	2.00	2.00	1.44	2.00	2.00	2.00	2.00	2.00	2.00	2.00	0.56	2.00	2.00	2.00	2.00	2.00		2.00	2.00	0.79	2.00	1.77	2.00	1.93	1.04	2.00	1.89
4B0H:A	1.55	2.00	2.00	2.00	1.26	2.00	2.00	2.00	2.00	1.50	2.00	2.00	2.00	2.00	2.00	2.00	1.62	2.00	2.00	2.00		0.06	2.00	0.29	2.00	1.40	2.00	2.00	0.33	1.86
4B0H:B	1.41	2.00	2.00	2.00	1.12	2.00	1.94	1.96	1.96	1.31	2.00	2.00	2.00	2.00	2.00	2.00	1.44	2.00	2.00	2.00	0.06		2.00	0.31	1.78	1.23	2.00	2.00	0.38	1.56
4FFY:A+H+L	1.38	2.00	2.00	2.00	2.00	0.91	2.00	1.87	1.87	1.46	2.00	2.00	1.89	0.52	2.00	2.00	2.00	2.00	2.00	0.79	2.00	2.00		2.00	0.69	1.65	1.13	0.30	2.00	0.72
4FFY:A	2.00	2.00	2.00	2.00	1.72	2.00	2.00	2.00	2.00	1.99	2.00	2.00	2.00	2.00	2.00	2.00	2.00	2.00	2.00	2.00	0.29	0.31	2.00		2.00	1.61	2.00	2.00	0.21	2.00
4FFY:H+L	0.74	2.00	2.00	2.00	1.70	1.39	2.00	1.07	1.07	0.74	2.00	2.00	2.00	0.91	2.00	2.00	1.66	2.00	2.00	1.77	2.00	1.78	0.69	2.00		0.78	0.62	1.04	2.00	0.09
4RXF:A	1.04	2.00	2.00	2.00	1.87	2.00	2.00	0.54	0.54	1.09	2.00	2.00	2.00	1.67	2.00	2.00	1.70	2.00	2.00	2.00	1.40	1.23	1.65	1.61	0.78		0.44	1.93	2.00	0.80
5AHE:A	1.19	2.00	2.00	2.00	2.00	1.61	2.00	0.56	0.56	1.19	2.00	2.00	2.00	0.97	2.00	2.00	2.00	2.00	2.00	1.93	2.00	2.00	1.13	2.00	0.62	0.44		1.28	2.00	0.66
5KVE:E+L	1.35	2.00	2.00	2.00	1.88	0.87	2.00	2.00	2.00	1.41	2.00	2.00	1.70	0.83	2.00	2.00	1.84	2.00	2.00	1.04	2.00	2.00	0.30	2.00	1.04	1.93	1.28		2.00	1.00
5KVE:E	2.00	2.00	2.00	2.00	2.00	2.00	2.00	2.00	2.00	2.00	2.00	2.00	2.00	2.00	2.00	2.00	2.00	2.00	2.00	2.00	0.33	0.38	2.00	0.21	2.00	2.00	2.00	2.00		2.00
5KVE:L	0.65	2.00	2.00	2.00	1.53	1.49	2.00	1.03	1.03	0.64	2.00	2.00	2.00	1.01	2.00	2.00	1.51	2.00	2.00	1.89	1.86	1.56	0.72	2.00	0.09	0.80	0.66	1.00	2.00	
