# Supplementary material for: Structural Outlier Detection and Zernike–Canterakis Moments for Molecular Surface Meshes—Fast Implementation in Python
Source: Molecules. 2023 Dec 21;29(1):52. doi: 10.3390/molecules29010052 (PMC10779519; doi:10.3390/molecules29010052)
Supplement: Supplementary file 1 [file molecules-29-00052-s001.zip › supplement4.pdf]

Figure S36. 3D Surfer comparison — backbone atoms mesh, outlier detection on, $2r_g$, Δ_z

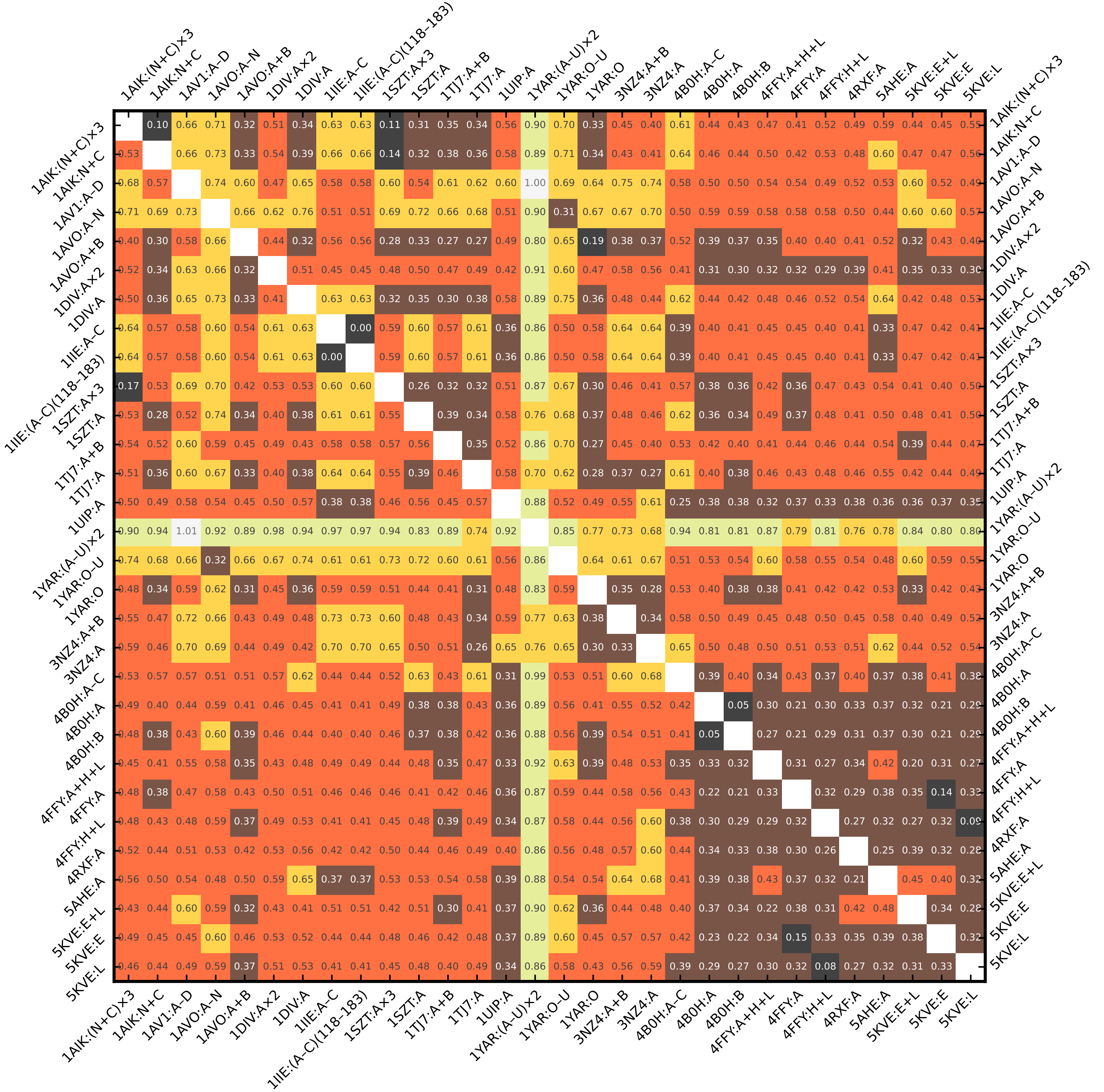

Figure S37. 3D Surfer comparison — backbone atoms mesh, outlier detection on, $2r_g$, Δ_{zd}

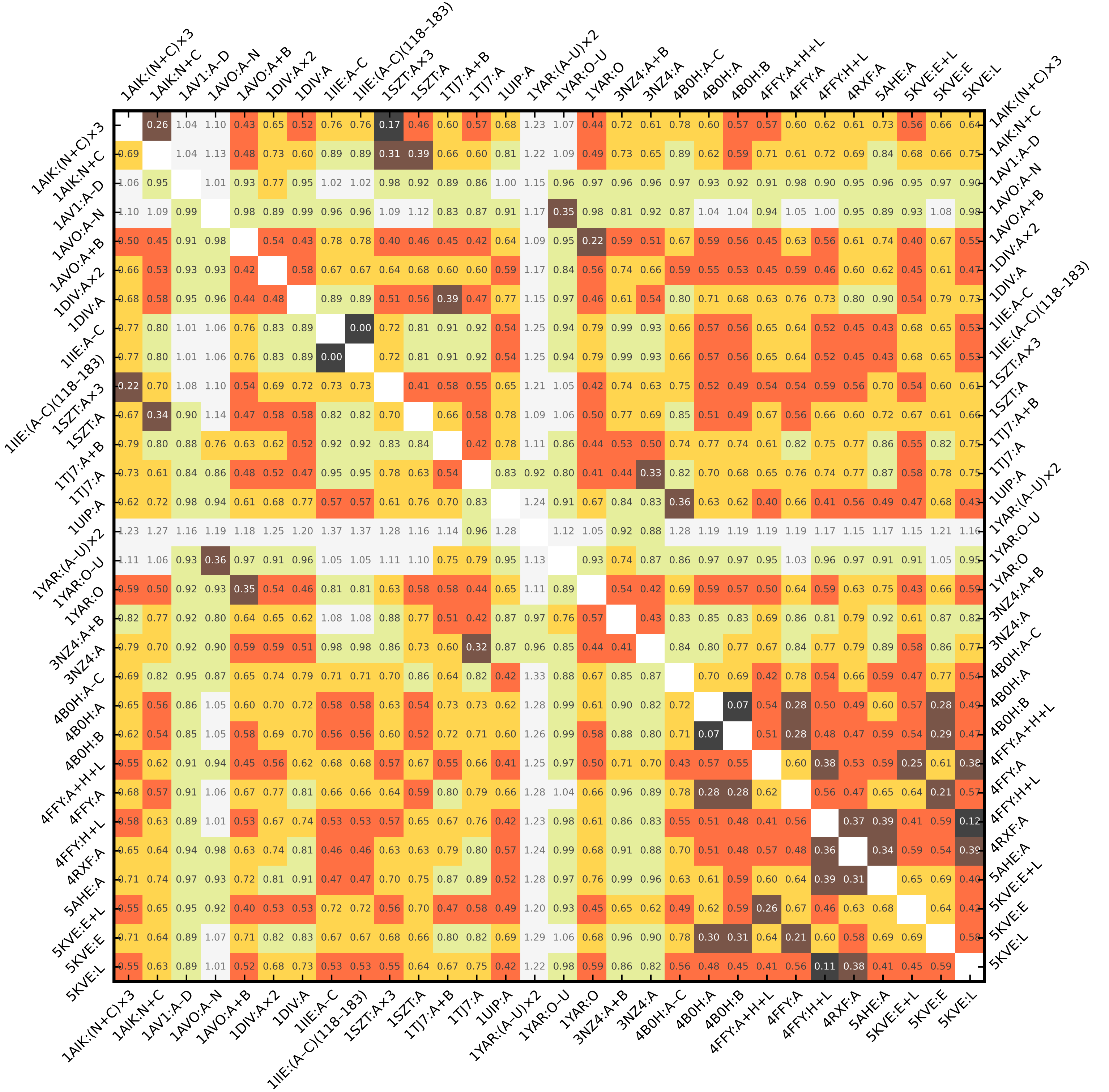

Figure S38. 3D Surfer comparison — backbone atoms mesh, outlier detection on, $2r_g$, Δ_{zdv}

	1AIK:(N+C)x3	1AIK:N+C	1AV1:A–D	1AVO:A–N	1AVO:A+B	1DIV:Ax2	1DIV:A	1IIE:A–C	1IIE:(A–C)(118–183)	1SZT:Ax3	1SZT:A	1TJ7:A+B	1TJ7:A	1UIP:A	1YAR:(A–U)x2	1YAR:O–U	1YAR:O	3NZ4:A+B	3NZ4:A	4B0H:A–C	4B0H:A	4B0H:B	4FFY:A+H+L	4FFY:A	4FFY:H+L	4RXF:A	5AHE:A	5KVE:E+L	5KVE:E	5KVE:L
1AIK:(N+C)x3		0.81	2.00	2.00	0.46	0.76	0.65	0.70	0.70	0.16	1.42	2.00	1.17	1.17	2.00	2.00	0.39	2.00	1.88	1.55	0.99	0.92	0.88	1.14	0.66	0.56	0.81	0.88	1.26	0.68
1AIK:N+C	2.00		2.00	2.00	1.27	2.00	1.38	2.00	2.00	0.94	0.36	2.00	2.00	2.00	2.00	2.00	1.53	2.00	2.00	2.00	1.09	1.06	2.00	0.93	2.00	2.00	2.00	2.00	1.00	2.00
1AV1:A–D	2.00	2.00		1.66	2.00	2.00	2.00	2.00	2.00	2.00	2.00	0.89	1.51	2.00	2.00	1.72	2.00	1.38	1.12	1.76	2.00	2.00	2.00	2.00	2.00	2.00	2.00	2.00	2.00	2.00
1AVO:A–N	2.00	2.00	1.63		2.00	2.00	2.00	2.00	2.00	2.00	2.00	1.52	2.00	2.00	2.00	0.34	2.00	0.84	1.93	2.00	2.00	2.00	2.00	2.00	2.00	2.00	2.00	2.00	2.00	2.00
1AVO:A+B	0.54	1.19	2.00	2.00		0.75	0.45	0.83	0.83	0.41	1.19	1.89	1.03	1.31	2.00	2.00	0.23	2.00	1.88	1.58	0.82	0.76	0.83	1.01	0.71	0.67	0.97	0.75	1.07	0.69
1DIV:Ax2	0.77	2.00	2.00	2.00	0.58		0.93	0.79	0.79	0.77	2.00	1.69	0.98	0.79	2.00	2.00	0.65	2.00	1.61	0.92	1.16	1.09	0.54	1.43	0.46	0.69	0.59	0.54	1.49	0.47
1DIV:A	0.85	1.33	2.00	2.00	0.46	0.77		1.10	1.10	0.61	1.25	1.92	1.33	1.82	2.00	2.00	0.57	2.00	2.00	2.00	0.85	0.80	1.33	1.05	1.08	1.02	1.37	1.16	1.10	1.07
1IIE:A–C	0.70	2.00	2.00	2.00	0.81	0.98	1.10		0.00	0.66	2.00	2.00	1.93	0.93	2.00	2.00	0.72	2.00	2.00	1.33	0.93	0.90	1.03	1.20	0.56	0.42	0.48	1.09	1.22	0.56
1IIE:(A–C)(118–183)	0.70	2.00	2.00	2.00	0.81	0.98	1.10	0.00		0.66	2.00	2.00	1.93	0.93	2.00	2.00	0.72	2.00	2.00	1.33	0.93	0.90	1.03	1.20	0.56	0.42	0.48	1.09	1.22	0.56
1SZT:Ax3	0.21	2.00	2.00	2.00	0.55	0.83	0.86	0.68	0.68		1.20	2.00	1.19	1.16	2.00	2.00	0.40	2.00	2.00	1.57	0.82	0.76	0.88	0.97	0.66	0.54	0.81	0.89	1.10	0.67
1SZT:A	2.00	0.31	2.00	2.00	1.22	2.00	1.31	2.00	2.00	2.00		2.00	2.00	2.00	2.00	2.00	1.54	2.00	2.00	2.00	0.88	0.85	2.00	0.84	2.00	1.87	2.00	2.00	0.90	2.00
1TJ7:A+B	2.00	2.00	0.88	1.38	2.00	1.74	2.00	2.00	2.00	2.00	2.00		0.67	1.49	2.00	1.71	1.58	0.85	0.52	1.21	2.00	2.00	1.30	2.00	2.00	2.00	2.00	1.16	2.00	2.00
1TJ7:A	1.52	2.00	1.48	2.00	1.17	0.83	1.34	1.99	1.99	1.69	2.00	0.85		0.91	2.00	2.00	0.86	1.22	0.46	0.77	2.00	2.00	0.80	2.00	1.31	1.56	1.48	0.69	2.00	1.34
1UIP:A	1.07	2.00	2.00	2.00	1.24	0.90	1.81	0.98	0.98	1.08	2.00	1.35	0.90		2.00	2.00	1.14	2.00	1.37	0.38	1.95	1.88	0.40	2.00	0.60	0.95	0.70	0.46	2.00	0.63
1YAR:(A–U)x2	2.00	2.00	2.00	2.00	2.00	2.00	2.00	2.00	2.00	2.00	2.00	2.00	2.00	2.00		2.00	2.00	2.00	2.00	2.00	2.00	2.00	2.00	2.00	2.00	2.00	2.00	2.00	2.00	2.00
1YAR:O–U	2.00	2.00	1.67	0.35	2.00	2.00	2.00	2.00	2.00	2.00	2.00	1.50	2.00	2.00	2.00		2.00	0.84	2.00	2.00	2.00	2.00	2.00	2.00	2.00	2.00	2.00	2.00	2.00	2.00
1YAR:O	0.53	1.55	2.00	2.00	0.37	0.62	0.58	0.73	0.73	0.59	1.77	2.00	0.92	1.12	2.00	2.00		2.00	1.30	1.39	0.97	0.92	0.78	1.21	0.63	0.57	0.83	0.68	1.25	0.62
3NZ4:A+B	2.00	2.00	1.33	0.83	2.00	2.00	2.00	2.00	2.00	2.00	2.00	0.80	1.14	2.00	2.00	0.86	2.00		0.78	2.00	2.00	2.00	2.00	2.00	2.00	2.00	2.00	2.00	2.00	2.00
3NZ4:A	2.00	2.00	1.07	1.90	2.00	1.44	2.00	2.00	2.00	2.00	2.00	0.63	0.44	1.45	2.00	1.96	1.36	0.75		1.19	2.00	2.00	1.23	2.00	2.00	2.00	2.00	1.06	2.00	2.00
4B0H:A–C	1.39	2.00	1.74	2.00	1.54	1.16	2.00	1.43	1.43	1.47	2.00	1.05	0.77	0.44	2.00	2.00	1.35	2.00	1.24		2.00	2.00	0.49	2.00	0.92	1.30	0.97	0.54	2.00	0.93
4B0H:A	1.07	0.97	2.00	2.00	0.84	1.49	0.86	0.94	0.94	0.99	0.92	2.00	2.00	1.90	2.00	2.00	1.00	2.00	2.00	2.00		0.06	1.51	0.29	0.97	0.83	1.20	1.62	0.29	0.93
4B0H:B	1.01	0.96	2.00	2.00	0.79	1.43	0.82	0.90	0.90	0.92	0.90	2.00	2.00	1.80	2.00	2.00	0.93	2.00	2.00	2.00	0.06		1.39	0.30	0.91	0.76	1.15	1.51	0.31	0.87
4FFY:A+H+L	0.86	2.00	2.00	2.00	0.82	0.68	1.33	1.07	1.07	0.92	2.00	1.16	0.81	0.41	2.00	2.00	0.78	2.00	1.29	0.51	1.60	1.50		1.91	0.50	0.81	0.75	0.23	1.96	0.50
4FFY:A	1.28	0.87	2.00	2.00	1.06	1.86	1.11	1.23	1.23	1.16	0.89	2.00	2.00	2.00	2.00	2.00	1.25	2.00	2.00	2.00	0.30	0.30	1.97		1.25	0.91	1.49	2.00	0.19	1.24
4FFY:H+L	0.61	2.00	2.00	2.00	0.67	0.65	1.09	0.57	0.57	0.64	2.00	2.00	1.34	0.61	2.00	2.00	0.65	2.00	2.00	0.94	0.98	0.91	0.54	1.25		0.39	0.36	0.55	1.31	0.11
4RXF:A	0.59	2.00	2.00	2.00	0.68	0.85	1.04	0.43	0.43	0.60	1.98	2.00	1.63	0.97	2.00	2.00	0.62	2.00	2.00	1.37	0.85	0.78	0.87	0.93	0.38		0.37	0.92	1.05	0.40
5AHE:A	0.78	2.00	2.00	2.00	0.94	0.77	1.38	0.53	0.53	0.80	2.00	2.00	1.52	0.74	2.00	2.00	0.84	2.00	2.00	1.04	1.23	1.16	0.77	1.47	0.37	0.33		0.83	1.60	0.38
5KVE:E+L	0.87	2.00	2.00	2.00	0.75	0.65	1.13	1.15	1.15	0.92	2.00	0.98	0.69	0.48	2.00	2.00	0.71	2.00	1.13	0.57	1.76	1.63	0.24	2.00	0.61	0.98	0.87		2.00	0.57
5KVE:E	1.35	0.97	2.00	2.00	1.14	1.99	1.14	1.26	1.26	1.23	0.97	2.00	2.00	2.00	2.00	2.00	1.29	2.00	2.00	2.00	0.32	0.33	2.00	0.19	1.34	1.11	1.59	2.00		1.28
5KVE:L	0.58	2.00	2.00	2.00	0.65	0.68	1.07	0.56	0.56	0.61	2.00	2.00	1.34	0.62	2.00	2.00	0.62	2.00	2.00	0.96	0.92	0.84	0.54	1.22	0.10	0.40	0.39	0.61	1.30	

Figure S39. 3D Surfer comparison — backbone atoms mesh, outlier detection on, $2r_g$, Δ_{zds}

	1AIK:(N+C)x3	1AIK:N+C	1AV1:A–D	1AVO:A–N	1AVO:A+B	1DIV:Ax2	1DIV:A	1IIE:A–C	1IIE:(A–C)(118–183)	1SZT:Ax3	1SZT:A	1TJ7:A+B	1TJ7:A	1UIP:A	1YAR:(A–U)x2	1YAR:O–U	1YAR:O	3NZ4:A+B	3NZ4:A	4B0H:A–C	4B0H:A	4B0H:B	4FFY:A+H+L	4FFY:A	4FFY:H+L	4RXF:A	5AHE:A	5KVE:E+L	5KVE:E	5KVE:L
1AIK:(N+C)x3		0.83	2.00	2.00	0.65	1.73	1.27	1.45	1.45	0.15	1.40	2.00	1.39	1.38	2.00	2.00	0.68	2.00	1.80	2.00	0.96	0.88	0.94	1.08	0.84	1.23	1.30	0.95	1.33	0.84
1AIK:N+C	2.00		2.00	2.00	1.48	2.00	2.00	2.00	2.00	1.02	0.55	2.00	2.00	2.00	2.00	2.00	1.62	2.00	2.00	2.00	1.82	1.83	2.00	1.87	2.00	2.00	2.00	2.00	1.77	2.00
1AV1:A–D	2.00	2.00		2.00	2.00	2.00	2.00	2.00	2.00	2.00	2.00	2.00	2.00	2.00	2.00	2.00	2.00	2.00	2.00	2.00	2.00	2.00	2.00	2.00	2.00	2.00	2.00	2.00	2.00	2.00
1AVO:A–N	2.00	2.00	2.00		2.00	2.00	2.00	2.00	2.00	2.00	2.00	1.63	2.00	2.00	2.00	0.32	2.00	2.00	2.00	2.00	2.00	2.00	2.00	2.00	2.00	2.00	2.00	2.00	2.00	2.00
1AVO:A+B	0.77	1.39	2.00	2.00		1.57	0.60	2.00	2.00	0.61	1.33	2.00	1.08	2.00	2.00	2.00	0.20	2.00	1.60	2.00	0.93	0.84	1.19	1.11	1.29	1.52	2.00	0.90	1.29	1.22
1DIV:Ax2	1.75	2.00	2.00	2.00	1.22		2.00	1.74	1.74	1.66	2.00	0.91	1.38	0.91	2.00	2.00	1.66	1.52	1.51	0.87	2.00	2.00	0.62	2.00	0.98	1.79	1.40	0.60	2.00	1.02
1DIV:A	1.65	1.97	2.00	2.00	0.61	2.00		2.00	2.00	1.26	1.77	2.00	1.10	2.00	2.00	2.00	0.61	2.00	1.53	2.00	1.80	1.63	2.00	2.00	2.00	2.00	2.00	1.87	2.00	2.00
1IIE:A–C	1.46	2.00	2.00	2.00	2.00	2.00	2.00		0.00	1.30	2.00	2.00	2.00	0.80	2.00	2.00	2.00	2.00	2.00	1.38	1.12	1.05	1.06	1.20	0.76	0.51	0.43	1.14	1.40	0.76
1IIE:(A–C)(118–183)	1.46	2.00	2.00	2.00	2.00	2.00	2.00	0.00		1.30	2.00	2.00	2.00	0.80	2.00	2.00	2.00	2.00	2.00	1.38	1.12	1.05	1.06	1.20	0.76	0.51	0.43	1.14	1.40	0.76
1SZT:Ax3	0.20	2.00	2.00	2.00	0.83	1.79	1.78	1.33	1.33		1.30	2.00	1.33	1.25	2.00	2.00	0.67	2.00	1.83	1.90	0.84	0.74	0.88	0.98	0.77	1.11	1.18	0.90	1.23	0.75
1SZT:A	2.00	0.48	2.00	2.00	1.36	2.00	1.84	2.00	2.00	2.00		2.00	2.00	2.00	2.00	2.00	1.56	2.00	2.00	2.00	1.52	1.53	2.00	1.74	2.00	2.00	2.00	2.00	1.65	2.00
1TJ7:A+B	2.00	2.00	2.00	1.49	2.00	0.94	2.00	2.00	2.00	2.00	2.00		1.22	2.00	2.00	1.60	1.99	0.81	1.44	1.48	2.00	2.00	1.49	2.00	2.00	2.00	2.00	1.31	2.00	2.00
1TJ7:A	1.80	2.00	2.00	2.00	1.22	1.18	1.12	2.00	2.00	1.89	2.00	1.55		2.00	2.00	2.00	0.99	0.98	0.35	2.00	2.00	2.00	1.35	2.00	1.49	2.00	2.00	1.23	2.00	1.50
1UIP:A	1.26	2.00	2.00	2.00	2.00	1.04	2.00	0.84	0.84	1.17	2.00	1.85	2.00		2.00	2.00	2.00	2.00	2.00	0.43	1.78	1.63	0.49	2.00	0.53	0.95	0.63	0.76	2.00	0.57
1YAR:(A–U)x2	2.00	2.00	2.00	2.00	2.00	2.00	2.00	2.00	2.00	2.00	2.00	2.00	2.00	2.00		2.00	2.00	2.00	2.00	2.00	2.00	2.00	2.00	2.00	2.00	2.00	2.00	2.00	2.00	2.00
1YAR:O–U	2.00	2.00	2.00	0.34	2.00	2.00	2.00	2.00	2.00	2.00	2.00	1.40	2.00	2.00	2.00		2.00	1.85	2.00	2.00	2.00	2.00	2.00	2.00	2.00	2.00	2.00	2.00	2.00	2.00
1YAR:O	0.92	1.64	2.00	2.00	0.32	1.59	0.62	2.00	2.00	0.99	1.79	2.00	1.07	2.00	2.00	2.00		2.00	1.22	2.00	0.96	0.87	1.34	1.18	1.38	1.58	2.00	0.98	1.36	1.32
3NZ4:A+B	2.00	2.00	2.00	2.00	2.00	1.32	2.00	2.00	2.00	2.00	2.00	0.77	0.92	2.00	2.00	1.89	2.00		0.91	2.00	2.00	2.00	2.00	2.00	2.00	2.00	2.00	1.97	2.00	2.00
3NZ4:A	2.00	2.00	2.00	2.00	1.83	1.35	1.45	2.00	2.00	2.00	2.00	1.73	0.33	2.00	2.00	2.00	1.27	0.87		2.00	2.00	2.00	1.47	2.00	1.80	2.00	2.00	1.38	2.00	1.87
4B0H:A–C	1.84	2.00	2.00	2.00	2.00	1.10	2.00	1.48	1.48	1.77	2.00	1.28	2.00	0.51	2.00	2.00	2.00	2.00	2.00		2.00	2.00	0.62	2.00	0.92	1.58	1.07	0.81	2.00	0.95
4B0H:A	1.03	1.63	2.00	2.00	0.96	2.00	1.81	1.13	1.13	1.02	1.60	2.00	2.00	1.74	2.00	2.00	0.99	2.00	2.00	2.00		0.06	1.66	0.27	1.01	0.74	1.12	1.80	0.30	0.95
4B0H:B	0.96	1.65	2.00	2.00	0.87	2.00	1.68	1.05	1.05	0.91	1.61	2.00	2.00	1.57	2.00	2.00	0.89	2.00	2.00	2.00	0.06		1.45	0.29	0.90	0.66	1.03	1.59	0.34	0.85
4FFY:A+H+L	0.91	2.00	2.00	2.00	1.18	0.78	2.00	1.11	1.11	0.92	2.00	1.33	1.37	0.50	2.00	2.00	1.36	2.00	1.55	0.63	1.75	1.57		2.00	0.50	0.98	0.82	0.28	2.00	0.51
4FFY:A	1.22	1.75	2.00	2.00	1.17	2.00	2.00	1.23	1.23	1.18	1.84	2.00	2.00	2.00	2.00	2.00	1.22	2.00	2.00	2.00	0.28	0.30	2.00		1.27	0.76	1.39	2.00	0.21	1.25
4FFY:H+L	0.79	2.00	2.00	2.00	1.22	1.40	2.00	0.77	0.77	0.75	2.00	2.00	1.52	0.53	2.00	2.00	1.43	2.00	1.95	0.94	1.02	0.90	0.53	1.28		0.63	0.52	0.69	1.49	0.11
4RXF:A	1.30	2.00	2.00	2.00	1.55	2.00	2.00	0.52	0.52	1.24	2.00	2.00	2.00	0.98	2.00	2.00	1.70	2.00	2.00	1.66	0.76	0.68	1.06	0.78	0.61		0.39	1.12	0.97	0.64
5AHE:A	1.25	2.00	2.00	2.00	2.00	1.81	2.00	0.47	0.47	1.17	2.00	2.00	2.00	0.67	2.00	2.00	2.00	2.00	2.00	1.14	1.16	1.04	0.84	1.36	0.53	0.35		0.95	1.65	0.53
5KVE:E+L	0.94	2.00	2.00	2.00	0.91	0.72	1.83	1.21	1.21	0.93	2.00	1.11	1.22	0.78	2.00	2.00	1.03	2.00	1.49	0.85	1.95	1.72	0.29	2.00	0.76	1.20	0.99		2.00	0.67
5KVE:E	1.42	1.70	2.00	2.00	1.36	2.00	2.00	1.44	1.44	1.39	1.77	2.00	2.00	2.00	2.00	2.00	1.40	2.00	2.00	2.00	0.33	0.36	2.00	0.21	1.52	1.03	1.64	2.00		1.43
5KVE:L	0.72	2.00	2.00	2.00	1.15	1.47	2.00	0.76	0.76	0.68	2.00	2.00	1.51	0.56	2.00	2.00	1.33	2.00	1.98	0.98	0.93	0.81	0.55	1.23	0.10	0.63	0.54	0.73	1.45	

Figure S40. 3D Surfer comparison — backbone atoms mesh, outlier detection on, $2r_g$, Δ_{zdsv}

	1AIK:(N+C)x3	1AIK:N+C	1AV1:A–D	1AVO:A–N	1AVO:A+B	1DIV:Ax2	1DIV:A	1IIE:A–C	1IIE:(A–C)(118–183)	1SZT:Ax3	1SZT:A	1TJ7:A+B	1TJ7:A	1UIP:A	1YAR:(A–U)x2	1YAR:O–U	1YAR:O	3NZ4:A+B	3NZ4:A	4B0H:A–C	4B0H:A	4B0H:B	4FFY:A+H+L	4FFY:A	4FFY:H+L	4RXF:A	5AHE:A	5KVE:E+L	5KVE:E	5KVE:L
1AIK:(N+C)x3		2.00	2.00	2.00	0.70	2.00	1.59	1.33	1.33	0.14	2.00	2.00	2.00	2.00	2.00	2.00	0.62	2.00	2.00	2.00	1.58	1.43	1.46	2.00	0.90	1.12	1.43	1.49	2.00	0.88
1AIK:N+C	2.00		2.00	2.00	2.00	2.00	2.00	2.00	2.00	2.00	0.51	2.00	2.00	2.00	2.00	2.00	2.00	2.00	2.00	2.00	2.00	2.00	2.00	2.00	2.00	2.00	2.00	2.00	2.00	2.00
1AV1:A–D	2.00	2.00		2.00	2.00	2.00	2.00	2.00	2.00	2.00	2.00	2.00	2.00	2.00	2.00	2.00	2.00	2.00	2.00	2.00	2.00	2.00	2.00	2.00	2.00	2.00	2.00	2.00	2.00	2.00
1AVO:A–N	2.00	2.00	2.00		2.00	2.00	2.00	2.00	2.00	2.00	2.00	2.00	2.00	2.00	2.00	0.32	2.00	2.00	2.00	2.00	2.00	2.00	2.00	2.00	2.00	2.00	2.00	2.00	2.00	2.00
1AVO:A+B	0.82	2.00	2.00	2.00		2.00	0.62	2.00	2.00	0.63	2.00	2.00	2.00	2.00	2.00	2.00	0.22	2.00	2.00	2.00	1.30	1.14	2.00	1.76	1.64	1.66	2.00	1.69	2.00	1.53
1DIV:Ax2	2.00	2.00	2.00	2.00	1.69		2.00	2.00	2.00	2.00	2.00	2.00	2.00	1.22	2.00	2.00	1.94	2.00	2.00	1.36	2.00	2.00	0.75	2.00	0.96	2.00	1.33	0.74	2.00	1.02
1DIV:A	2.00	2.00	2.00	2.00	0.64	2.00		2.00	2.00	1.50	2.00	2.00	2.00	2.00	2.00	2.00	0.77	2.00	2.00	2.00	2.00	1.91	2.00	2.00	2.00	2.00	2.00	2.00	2.00	2.00
1IIE:A–C	1.33	2.00	2.00	2.00	2.00	2.00	2.00		0.00	1.21	2.00	2.00	2.00	1.39	2.00	2.00	2.00	2.00	2.00	2.00	1.83	1.68	1.66	2.00	0.82	0.47	0.48	1.82	2.00	0.81
1IIE:(A–C)(118–183)	1.33	2.00	2.00	2.00	2.00	2.00	2.00	0.00		1.21	2.00	2.00	2.00	1.39	2.00	2.00	2.00	2.00	2.00	2.00	1.83	1.68	1.66	2.00	0.82	0.47	0.48	1.82	2.00	0.81
1SZT:Ax3	0.19	2.00	2.00	2.00	0.85	2.00	2.00	1.23	1.23		2.00	2.00	2.00	2.00	2.00	2.00	0.63	2.00	2.00	2.00	1.33	1.15	1.42	1.78	0.85	1.06	1.36	1.48	2.00	0.83
1SZT:A	2.00	0.44	2.00	2.00	2.00	2.00	2.00	2.00	2.00	2.00		2.00	2.00	2.00	2.00	2.00	2.00	2.00	2.00	2.00	2.00	2.00	2.00	2.00	2.00	2.00	2.00	2.00	2.00	2.00
1TJ7:A+B	2.00	2.00	2.00	2.00	2.00	2.00	2.00	2.00	2.00	2.00	2.00		1.94	2.00	2.00	2.00	2.00	1.28	1.50	2.00	2.00	2.00	2.00	2.00	2.00	2.00	2.00	2.00	2.00	2.00
1TJ7:A	2.00	2.00	2.00	2.00	2.00	1.91	2.00	2.00	2.00	2.00	2.00	2.00		2.00	2.00	2.00	2.00	2.00	0.48	2.00	2.00	2.00	1.64	2.00	2.00	2.00	2.00	1.47	2.00	2.00
1UIP:A	2.00	2.00	2.00	2.00	2.00	1.39	2.00	1.46	1.46	2.00	2.00	2.00	2.00		2.00	2.00	2.00	2.00	2.00	0.46	2.00	2.00	0.49	2.00	0.77	1.61	0.89	0.75	2.00	0.85
1YAR:(A–U)x2	2.00	2.00	2.00	2.00	2.00	2.00	2.00	2.00	2.00	2.00	2.00	2.00	2.00	2.00		2.00	2.00	2.00	2.00	2.00	2.00	2.00	2.00	2.00	2.00	2.00	2.00	2.00	2.00	2.00
1YAR:O–U	2.00	2.00	2.00	0.33	2.00	2.00	2.00	2.00	2.00	2.00	2.00	2.00	2.00	2.00	2.00		2.00	2.00	2.00	2.00	2.00	2.00	2.00	2.00	2.00	2.00	2.00	2.00	2.00	2.00
1YAR:O	0.83	2.00	2.00	2.00	0.34	1.86	0.77	2.00	2.00	0.93	2.00	2.00	2.00	2.00	2.00	2.00		2.00	2.00	2.00	1.59	1.41	2.00	2.00	1.48	1.45	2.00	1.54	2.00	1.39
3NZ4:A+B	2.00	2.00	2.00	2.00	2.00	2.00	2.00	2.00	2.00	2.00	2.00	1.22	2.00	2.00	2.00	2.00	2.00		1.67	2.00	2.00	2.00	2.00	2.00	2.00	2.00	2.00	2.00	2.00	2.00
3NZ4:A	2.00	2.00	2.00	2.00	2.00	2.00	2.00	2.00	2.00	2.00	2.00	1.81	0.46	2.00	2.00	2.00	2.00	1.60		2.00	2.00	2.00	2.00	2.00	2.00	2.00	2.00	2.00	2.00	2.00
4B0H:A–C	2.00	2.00	2.00	2.00	2.00	1.71	2.00	2.00	2.00	2.00	2.00	2.00	2.00	0.54	2.00	2.00	2.00	2.00	2.00		2.00	2.00	0.72	2.00	1.56	2.00	1.76	0.93	2.00	1.64
4B0H:A	1.71	2.00	2.00	2.00	1.33	2.00	2.00	1.85	1.85	1.61	2.00	2.00	2.00	2.00	2.00	2.00	1.63	2.00	2.00	2.00		0.06	2.00	0.28	1.96	1.24	2.00	2.00	0.32	1.82
4B0H:B	1.56	2.00	2.00	2.00	1.18	2.00	1.97	1.67	1.67	1.41	2.00	2.00	2.00	2.00	2.00	2.00	1.43	2.00	2.00	2.00	0.06		2.00	0.31	1.71	1.09	2.00	2.00	0.37	1.59
4FFY:A+H+L	1.41	2.00	2.00	2.00	2.00	0.94	2.00	1.74	1.74	1.49	2.00	2.00	1.67	0.50	2.00	2.00	2.00	2.00	2.00	0.74	2.00	2.00		2.00	0.66	1.50	1.04	0.26	2.00	0.68
4FFY:A	2.00	2.00	2.00	2.00	1.86	2.00	2.00	2.00	2.00	2.00	2.00	2.00	2.00	2.00	2.00	2.00	2.00	2.00	2.00	2.00	0.29	0.31	2.00		2.00	1.47	2.00	2.00	0.19	2.00
4FFY:H+L	0.84	2.00	2.00	2.00	1.55	1.38	2.00	0.84	0.84	0.83	2.00	2.00	2.00	0.77	2.00	2.00	1.53	2.00	2.00	1.60	1.98	1.70	0.70	2.00		0.66	0.49	0.92	2.00	0.10
4RXF:A	1.19	2.00	2.00	2.00	1.69	2.00	2.00	0.48	0.48	1.19	2.00	2.00	2.00	1.65	2.00	2.00	1.56	2.00	2.00	2.00	1.28	1.11	1.62	1.50	0.64		0.42	1.74	1.88	0.66
5AHE:A	1.38	2.00	2.00	2.00	2.00	1.73	2.00	0.52	0.52	1.34	2.00	2.00	2.00	0.95	2.00	2.00	2.00	2.00	2.00	1.88	2.00	2.00	1.07	2.00	0.49	0.38		1.22	2.00	0.50
5KVE:E+L	1.47	2.00	2.00	2.00	1.69	0.88	2.00	1.93	1.93	1.53	2.00	2.00	1.47	0.77	2.00	2.00	1.62	2.00	2.00	0.98	2.00	2.00	0.27	2.00	1.02	1.85	1.28		2.00	0.91
5KVE:E	2.00	2.00	2.00	2.00	2.00	2.00	2.00	2.00	2.00	2.00	2.00	2.00	2.00	2.00	2.00	2.00	2.00	2.00	2.00	2.00	0.34	0.39	2.00	0.19	2.00	2.00	2.00	2.00		2.00
5KVE:L	0.76	2.00	2.00	2.00	1.44	1.47	2.00	0.81	0.81	0.75	2.00	2.00	2.00	0.83	2.00	2.00	1.40	2.00	2.00	1.69	1.79	1.53	0.73	2.00	0.09	0.66	0.51	0.98	2.00	
